# Deactivating siRNA activity with bioorthogonal inverse-electron-demand Diels–Alder chemistry

**DOI:** 10.1039/d6cb00284f

**Published:** 2026-10-12

**Authors:** Kaifeng Zhao, Kaleena Basran, Loïc Courville-Fortin, Nathan W. Luedtke

**Affiliations:** a Department of Chemistry, McGill University Montréal H3A 0B8 Canada nathan.luedtke@mcgill.ca; b Department of Pharmacology and Therapeutics, McGill University Montréal H3G 1Y6 Canada

## Abstract

Small interfering RNAs (siRNAs) were prepared with vinyl nucleobases and imperfect duplex reaction sites that enable selective inverse electron-demand Diels–Alder (IEDDA) reactions with tetrazines in living cells. These bioorthogonal reactions attenuate gene silencing of both reporter and endogenous genes, establishing a small-molecule strategy for deactivating siRNA activity.

## Abstract 2

Vinyl-modified siRNAs bearing reaction sites within imperfectly paired duplex regions undergo selective IEDDA reactions with tetrazines in live cells, attenuating silencing of reporter and endogenous genes and enabling small-molecule control of RNAi.

## Graphical abstract

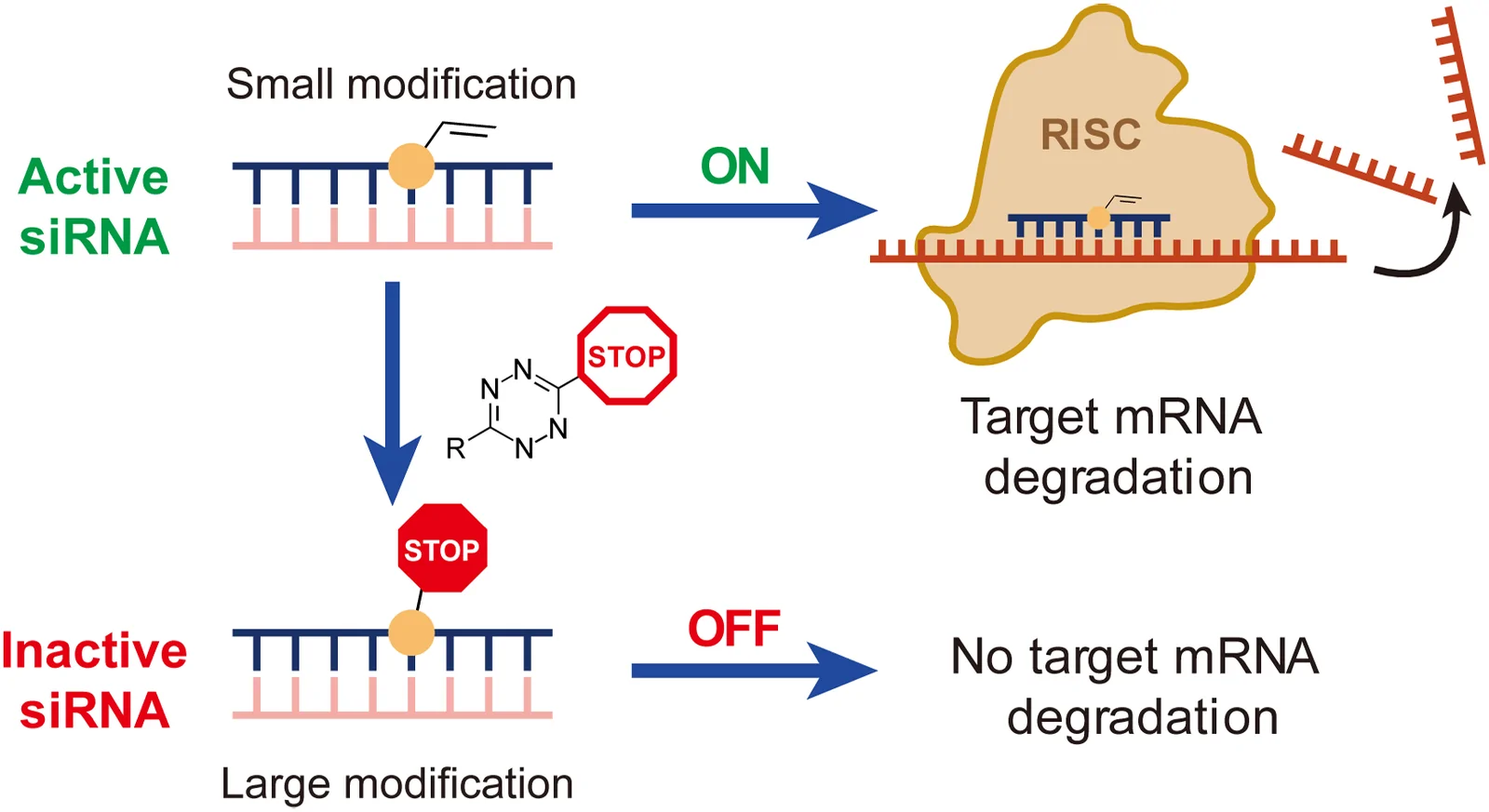

RNA interference (RNAi) therapeutics have benefited from advanced chemical modifications of oligonucleotides^1–3^ that enable durable silencing effects for up to 2 years.^4^ However, the extended duration of many small interfering RNA (siRNA) therapeutics creates a need for strategies that allow post-administration control of RNAi activity.^5^ For example, antidotes for long-acting angiotensinogen-targeted siRNA (Zilebesiran) might be required for rapid restoration of renin–angiotensin system function to maintain blood pressure during acute clinical situations such as sepsis and hemorrhage.^6^ Complementary oligonucleotides targeting siRNA or microRNA guide strands have been widely explored as antidotes.^7,8^ Small-molecule approaches could offer attractive pharmacological advantages over oligonucleotide antidotes, including flexible routes of administration.^1^

Chemical modification of siRNAs can potentially enhance their pharmacological properties while simultaneously introducing bioorthogonal reactivity. Bioorthogonal activation strategies for therapeutic oligonucleotides have previously been explored across multiple modalities including siRNAs,^9^ antisense oligonucleotides (ASOs),^10^ CRISPR-Cas single-guide RNAs (sgRNAs),^11^ mRNAs,^12^ and aptamers.^13^ Comparatively fewer deactivation strategies have been reported,^14^ and selective chemical deactivation of siRNAs remains challenging because siRNA duplexes can sterically restrict access to reactive groups within the double helix.^15^ In previous work, we developed the acridine–tetrazine probe PINK (Probe for Imaging Nucleosidic alKene groups), which undergoes intercalation-enhanced inverse electron-demand Diels–Alder (IEDDA) reactions within duplex nucleic acids.^16,17^ As compared to strain-promoted azide–alkyne click and other catalyst-free bioorthogonal chemical reactions, IEDDA reactions exhibit faster reaction kinetics,^18^ as well as excellent compatibility with nucleic acids.^17,19,20^ IEDDA reactions have even progressed into clinical trials for pre-targeted molecular imaging and click-to-release activation of therapeutics.^21^ We therefore investigated whether engineered siRNA duplexes could support selective tetrazine ligation reactions for small-molecule-mediated siRNA deactivation (Scheme 1).

**Scheme 1:**
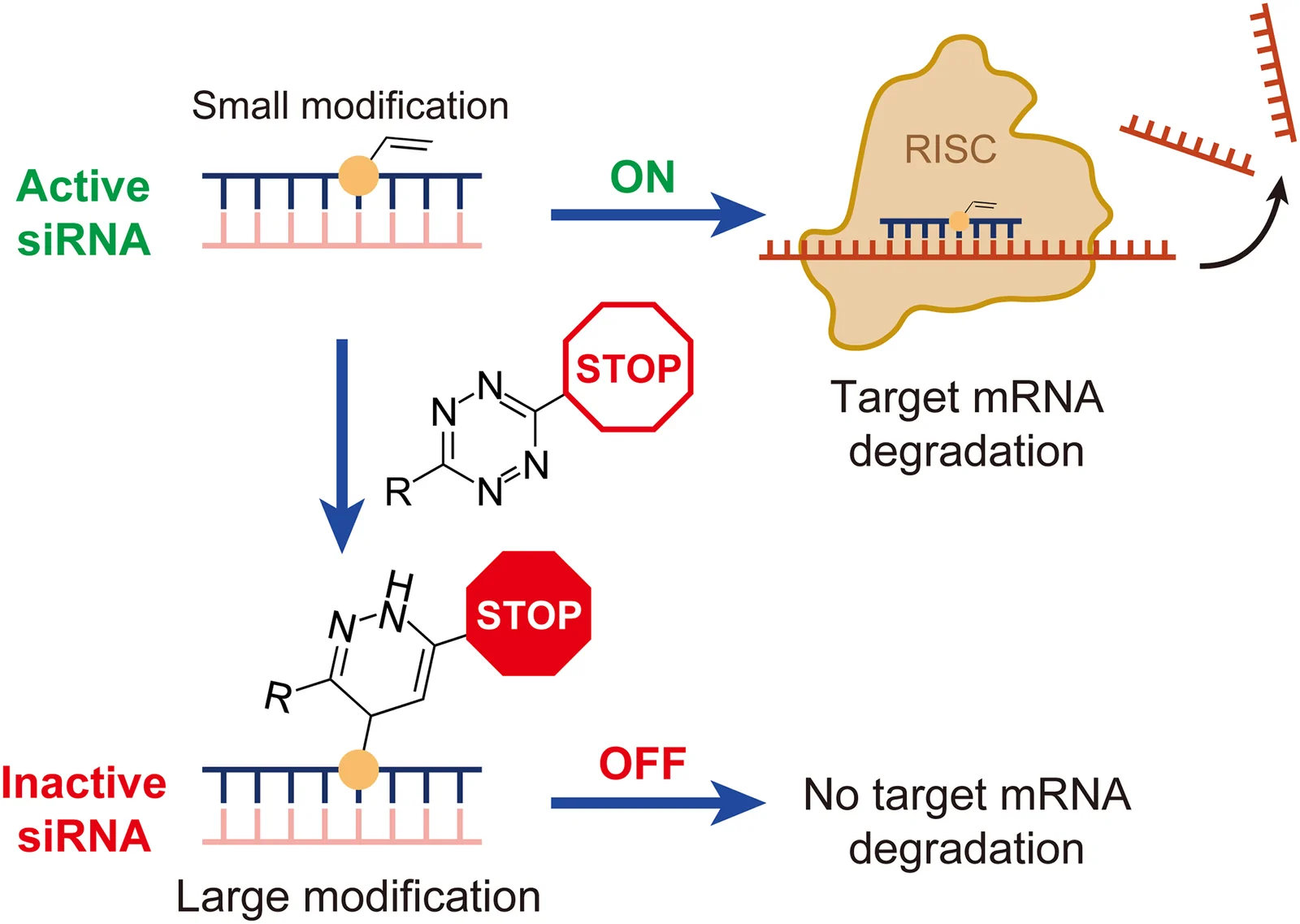
Schematic illustration of deactivating siRNA with bioorthogonal IEDDA reactions.

To introduce minimally perturbing IEDDA-reactive groups into siRNA duplexes,^3,22^ we used standard solid-phase oligonucleotide synthesis conditions to incorporate 5-vinyl-2′-deoxyuridine (VdU) and 7-vinyl-7-deaza-2′-deoxyadenosine (VdA) into siRNAs targeting the firefly luciferase gene (Fig. 1A and B and Tables S1 and S2).^23–25^ VdU was incorporated within the seed region at positions g2, g5, or g6, which is essential for target mRNA recognition, or at position g14, which is critical to RISC (RNA-induced silencing complex) loading and catalytic activity.^23–25^ VdA was incorporated at neighbouring positions g3, g4, or g7 to compare the deactivation effects with those of the VdU substitutions. Upon transfection into HeLa X1/5 cells, a HeLa-derived cell line that stably expresses firefly luciferase,^26^ all VdU- and VdA-modified siRNAs exhibited approximately 95% knockdown (Fig. 1C), similar to that of the parent siRNA lacking any vinyl modifications (d1.6). These results demonstrate that VdU and VdA are compatible with potent RNAi activity, consistent with previous reports that deoxynucleotide substitutions are well tolerated in siRNAs.^27^

**Fig. 1:**
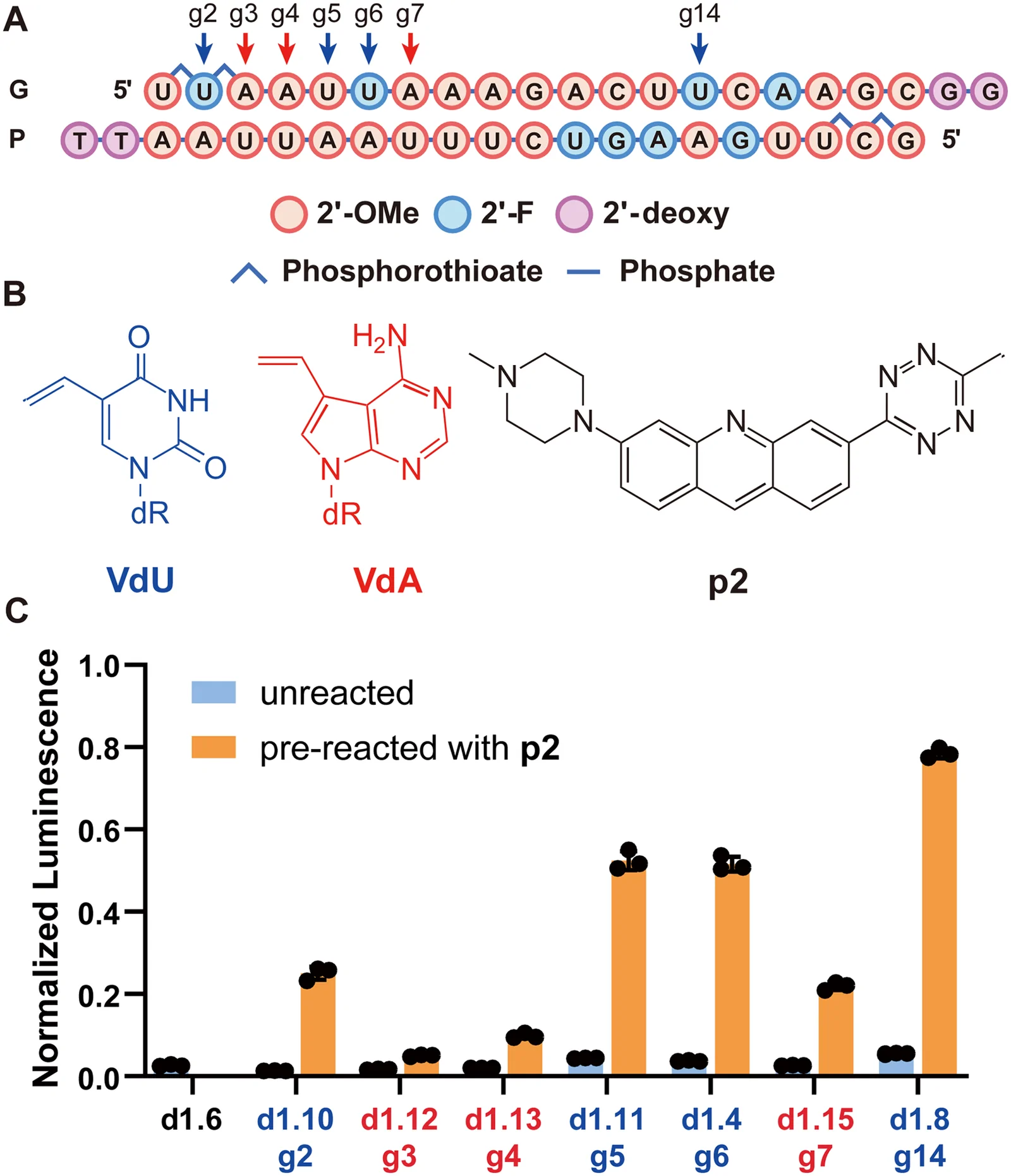
(A) Sequences and chemical modifications of firefly luciferase-targeting siRNAs. Arrows indicate VdU (blue) and VdA (red) substitution sites. G: guide strand, P: passenger strand. (B) Structures of VdU, VdA, and p2. dR = 2′-deoxyribose. See Fig. S1 for the synthesis of p2. (C) Silencing activities of VdU (blue duplex names) or VdA (red duplex names) siRNA duplexes before and after reaction with p2. Substitution positions are indicated below the name of each duplex. Luminescence signals were normalized to cells treated with scrambled siRNA control **d0.2**. Data represent means ± SD (*n* = 3).

Next, we evaluated how bulky IEDDA-derived nucleobase modifications can impact the silencing activity of siRNA duplexes. Because pre-reacted duplexes can mimic the products of intracellular IEDDA ligation, they served as positive controls for siRNA deactivation and enabled us to identify the optimal position for installing the bioorthogonal handle. A new water-soluble PINK derivative, p2 (Fig. 1B, see Fig. S2 for intercalation activity), was reacted with each vinyl-modified guide strand. The major 1 : 1 p2 adduct from each reaction was isolated and annealed with the passenger strand (see Table S1 and Fig. S15–S21 for analytical data). The modified duplexes were transfected into HeLa X1/5 cells, and their silencing efficiencies were evaluated using luminescence (Fig. 1C). To our surprise, some of the bulky IEDDA p2 adducts on VdU and VdA siRNAs were compatible with RNAi at certain positions. In general, the p2-VdU-modified sites (g2, g5, g6, and g14) were much more sensitive to loss of silencing activity as compared to the p2-VdA adducts at g3, g4, and g7. Among all tested VdU positions, the p2-VdU reaction product at position g14 (d1.8) located outside the seed region exhibited the largest decrease in siRNA activity, suggesting that the IEDDA product primarily disrupts siRNA loading or catalytic functions rather than mRNA recognition. We therefore selected VdU substitution at position g14 to investigate chemically induced siRNA deactivation by p2 in living cells. HeLa X1/5 cells were transfected with 2 nM of duplex siRNA d1.8 for 18 h, followed by incubation with 0.75 µM of p2 for 4 days. However, no significant differences in luminescence between p2-treated and untreated cells were observed (Fig. S3). Higher doses of p2 caused cell death under these conditions (Fig. S4), likely due to its ability to enter cells and non-specifically intercalate into genomic DNA (Fig. S2). Taken together, these results highlight the limitations of intercalator-based siRNA antidote strategies. We therefore engineered siRNA duplexes containing reactive sites designed to enable ligation with non-toxic, non-intercalating tetrazines.

Imperfect duplex features such as mismatches and bulges can enable site-specific small-molecule binding interactions.^28,29^ Mismatches and bulges are also known to be compatible with siRNA activity.^30,31^ To test this possibility for VdU-containing siRNAs, we introduced a mismatch (d1.1, A–A mismatch), an abasic substitution (d2.1, containing a C3 linker) or an abasic bulge (d4.1, also containing a C3 linker) on the passenger strand of the siRNA (Fig. 2A). In parallel, we made a small library of tetrazines (Fig. 2B) including quinoline-linked tetrazines (tz6, tz7, tz8, and tz16),^32^ motivated by reports of weak nucleic-acid binding by the malaria drug hydroxychloroquine.^33,34^ The reactivities of the tetrazines toward the engineered siRNA duplexes, dsDNA, and the VdU nucleoside were evaluated using HPLC (Fig. S5–S11). Percent conversion values for the VdU nucleoside and VdU-containing duplexes with excess tetrazine are summarized in Fig. 2A. Consistent with our previous studies, simple tetrazines such as tz1 and tz2 reacted with the VdU nucleoside, but not with the VdU-containing duplexes.^35^ In contrast, the intercalating tetrazine p2 was significantly more reactive towards all of the VdU-containing duplexes as compared to VdU itself. Consistent with our design strategy, the quinoline-modified tetrazines showed little or no reactivity toward the perfectly matched siRNA or DNA duplexes but exhibited selectivity for engineered siRNAs containing A–A mismatches or abasic C3 spacers. Consistent with these results, neither tz6 nor tz8 exhibited evidence of dsDNA intercalation (Fig. S2), and both compounds were significantly less toxic to cells than p2 (Fig. S4).

**Fig. 2:**
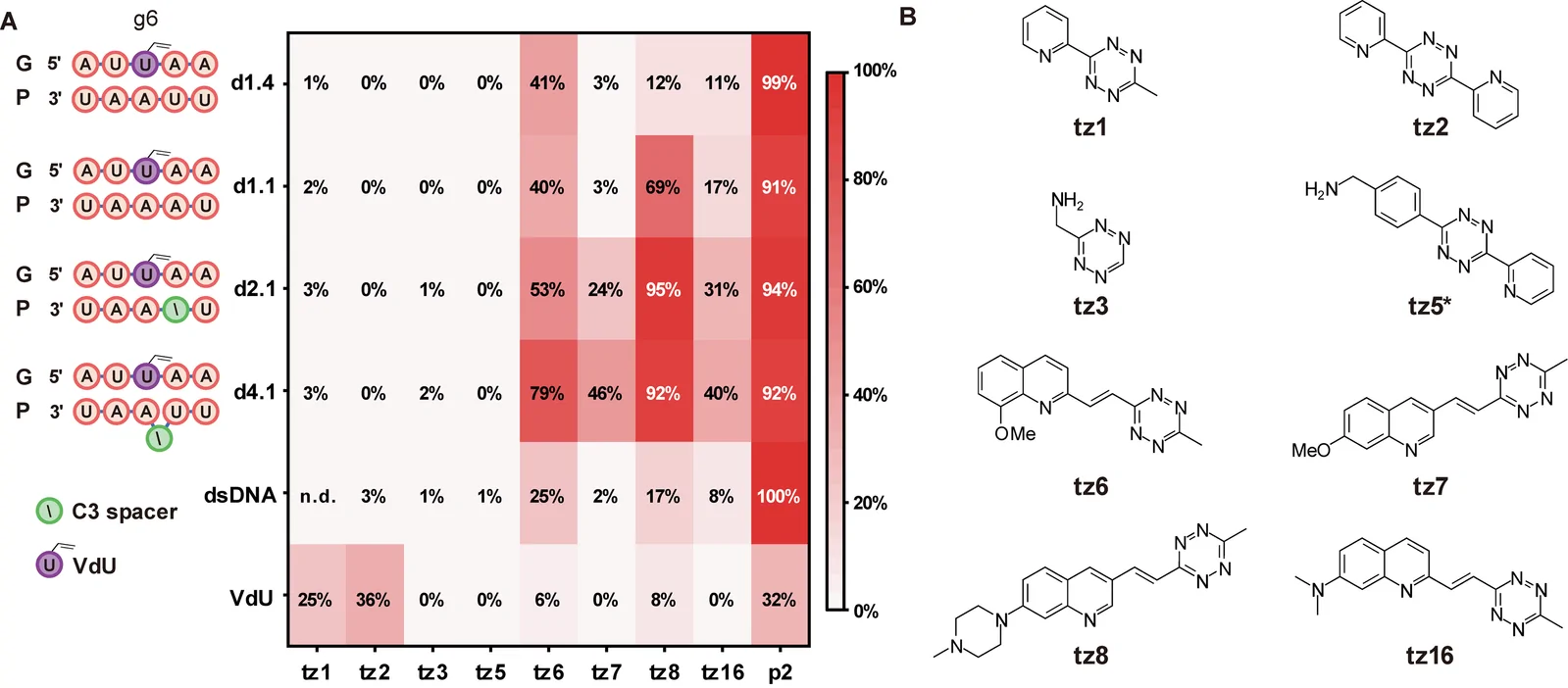
Reactivity of tetrazines toward engineered siRNA duplexes. (A) Partial structures of engineered siRNA duplexes and corresponding conversion values for VdU-containing strands or the VdU nucleoside following IEDDA reactions with tetrazines for 6 h (duplexes) or 24 h (VdU nucleoside) at 37 °C. Raw HPLC traces are provided in Fig. S5–S11. G, guide strand; P, passenger strand; n.d., not determined. (B) Structures of tetrazines investigated in this study, * indicates low water solubility of tz5.

To evaluate the non-covalent binding interactions of tz6/tz8 with the siRNA duplexes, we performed a competitive displacement assay using Neo-BODIPY, a known RNA major-groove-binding probe.^36^ Both tetrazines were able to displace Neo-BODIPY from the siRNA duplexes, with tz8 showing enhanced displacement for the siRNA containing the C3 abasic bulge (Fig. S12) as compared to the perfect duplex. Taken together with the positioning of the VdU vinyl group within the duplex major groove, these results suggest that tz6 and tz8 have non-covalent binding interactions in the major groove of RNA that promote selective IEDDA reactions with VdU-containing siRNAs.

Based on the positional scanning results (Fig. 1) and reaction-site engineering studies (Fig. 2), we designed siRNA duplex d4.11, which contains a VdU substitution at guide position g14 and an abasic bulge on the passenger strand (Fig. 3A). Duplex d4.12 contains an additional VdU substitution at guide position g13. The lower toxicity of the non-intercalating tetrazines allowed us to evaluate the siRNAs in cellular deactivation assays at a higher tetrazine concentration (7.5 µM) than that used for p2. As shown in Fig. 3A, tz6 significantly reduced the silencing activity of both d4.11 and d4.12 after 4 days. In contrast, tz8 caused an unexpected nonspecific increase in luminescence across all samples, including the scrambled siRNA control (Fig. S13 and S14). Importantly, neither tz6 nor tz8 significantly altered the silencing activity of the perfectly matched duplex d1.8, supporting selective ligation within engineered siRNA duplexes containing structurally accessible reaction sites. These results suggest that IEDDA reactions occur predominantly prior to RISC loading, while the siRNA remains in a duplex form.

**Fig. 3:**
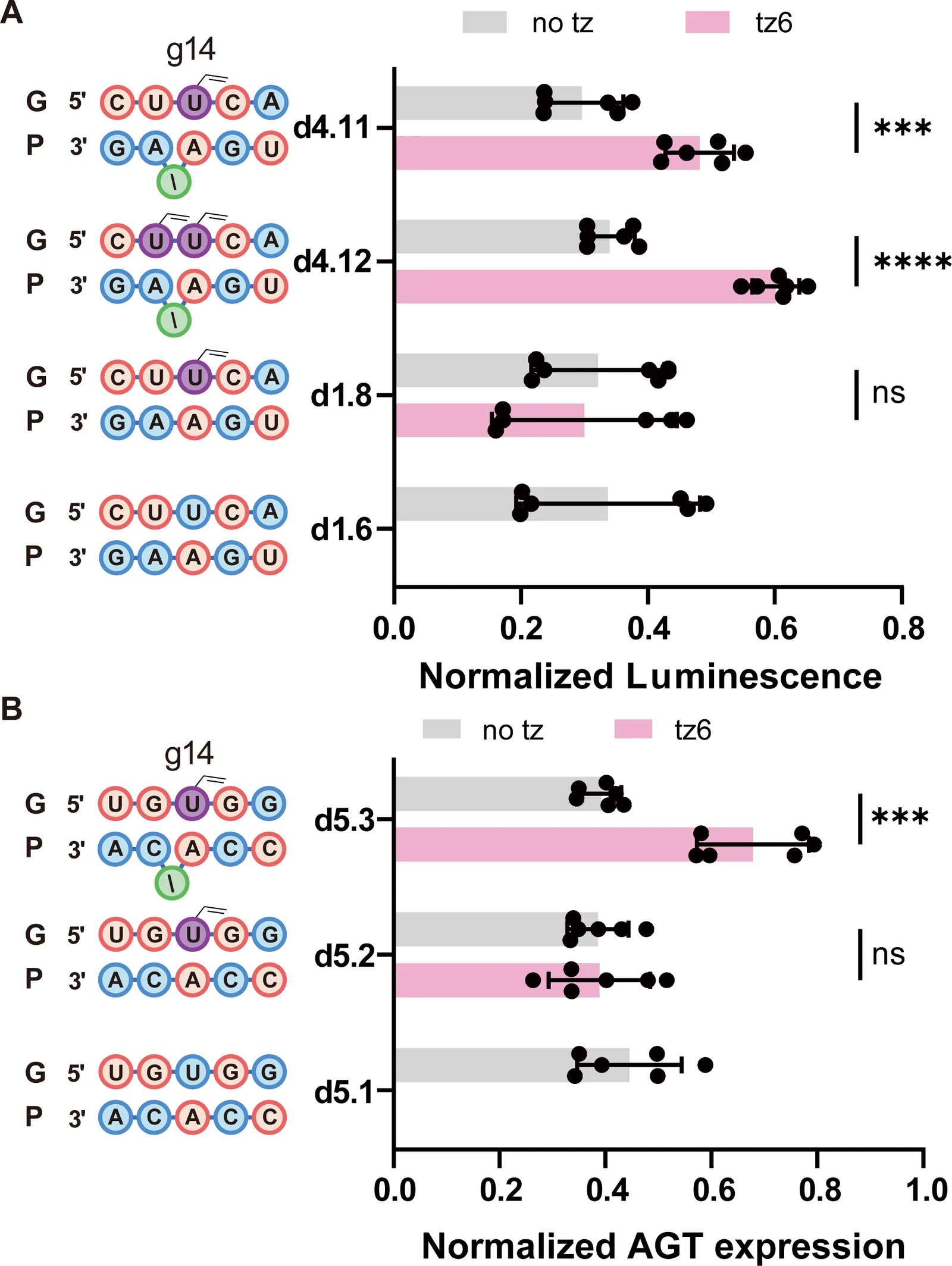
Small-molecule deactivation of modified siRNAs in living cells. (A) Normalized luminescence signals from HeLa X1/5 cell lysates treated with 2 nM of each siRNA followed by tz6. (B) Normalized AGT expression levels in HepG2 cells following treatment with each siRNA (2 nM) and with or without tz6 (7.5 µM). Structures of the corresponding siRNA duplexes are shown at the left. G, guide strand; P, passenger strand. Data represent means ± SD (*n* = 6). ns, *p* > 0.05; ***, *p* < 0.001; ****, *p* < 0.0001 (Student's *t*-test). No significant differences were observed among the no-tetrazine control groups (one-way ANOVA, *p* = 0.8542 for panel A, and *p* = 0.3004 for panel (B)).

To evaluate silencing reversal of an endogenous gene, we applied our design strategy to Zilebesiran, an investigational RNAi therapeutic targeting angiotensinogen (AGT) for the treatment of hypertension.^5,37,38^ AGT is the upstream precursor of the renin–angiotensin system (RAS),^39^ and complete AGT knockdown has been shown to cause hypotension.^40^ Strategies that enable post-administration control of AGT-targeted siRNAs could improve the safety and reversibility of this therapeutic modality.^6^

Using the chemical modification pattern of Zilebesiran as a template, we introduced VdU at guide position g14 together with an abasic bulge analogous to d4.11 to give the engineered siRNA duplex d5.3 (Fig. 3B; full sequences and modifications are provided in Tables S1 and S2). HepG2 cells were transfected with 2 nM of d5.3 for 18 h, followed by incubation with tz6 (7.5 µM) for 7 days. The longer 7-day incubation period was chosen for HepG2 cells because they proliferate more slowly than HeLa X1/5 cells. RT-qPCR analysis revealed substantial reversal of AGT silencing following tz6 treatment (Fig. 3B). In contrast, tz6 did not significantly alter the activity of the perfectly matched duplex d5.2. Importantly, the silencing activities of d5.2 and d5.3 were comparable to those of the parent Zilebesiran-derived duplex d5.1, demonstrating that VdU incorporation and reaction-site engineering are well tolerated in therapeutically relevant siRNA architectures. Collectively, these results demonstrate selective small-molecule-mediated deactivation of engineered siRNAs targeted to both reporter and endogenous genes in living cells.

In summary, we developed the first small-molecule strategy for bioorthogonal siRNA deactivation in living cells. By combining vinyl-modified nucleobases with engineered siRNA duplex architectures, selective IEDDA reactions within double-stranded siRNA structures were achieved by weakly interacting, non-intercalating tetrazines. This duplex-engineering strategy permitted efficient attenuation of RNAi against both reporter and endogenous gene targets while preserving the intrinsic silencing activity of the modified siRNAs prior to ligation. Mechanistically, these studies demonstrate that weak major-groove interactions can direct selective bioorthogonal reactions within modified duplexes. Beyond establishing a strategy for chemically controlled modulation of RNAi, this work expands the scope of bioorthogonal chemistry and provides a general framework for engineering chemically addressable oligonucleotide therapeutics.

## Conflicts of interest

There are no conflicts to declare.

## Supplementary Material

CB-OLF-D6CB00284F-s001

## Data Availability

The data supporting this article have been included as part of the supplementary information (SI). Supplementary information is available. See DOI: https://doi.org/10.1039/d6cb00284f.
