# Supplementary material for: Deactivating siRNA activity with bioorthogonal inverse-electron-demand Diels–Alder chemistry
Source: RSC Chem Biol. 2026 Oct 12. Online ahead of print. doi: 10.1039/d6cb00284f (PMC13725365; doi:10.1039/d6cb00284f)
Supplement: Supplementary file 1 [file CB-OLF-D6CB00284F-s001.pdf]

Supplementary Information (SI) for RSC Chemical Biology.
This journal is © The Royal Society of Chemistry

Deactivating siRNA Activity with Bioorthogonal Inverse-electron-demand Diels–Alder Chemistry

Kaifeng Zhao, Kaleena Basran, Loïc Courville-Fortin, and Nathan W. Luedtke*

Table of Contents

Methods and Materials 3

Supplementary Tables 7

Supplementary figures and discussions 9

LC-MS data for p2-modified guide strands 19

Synthesis procedures 26

NMR spectra 37

References 60

Methods and Materials

General synthesis methods and materials

All reagents were obtained from Sigma-Aldrich, Fisher Scientific, Oakwood or Aaron Chemicals without further purification. **tz4**, **VdU** and its phosphoramidite were synthesized according to references.[1, 2] Reactions that are sensitive to air or water were performed under Ar atmosphere using anhydrous solvents and standard Schlenk techniques. Anhydrous DCM, toluene, THF and 1,4-dioxane were obtained with a MBraun solvent purification system. Degassed solvent for cross-coupling reactions was obtained by evacuating the solvents under sonication followed by refilling with Ar 3 times. Analytical thin layer chromatography (TLC) was conducted on aluminum-backed silica gel plates (200 µm thick with F254 indicator, Silicycle) and visualized with UV light (254 nm or 365 nm for inherent fluorescence) or I_2 staining. Silica column chromatography was performed with irregular silica gel (40-63 µm, Sigma-Aldrich) under compressed air. Nuclear magnetic resonance (NMR) spectra were measured on a Bruker AVIII 400 (400 MHz for ^{1}H, 101 MHz for ^{13}C) or AVIIIHD 500 (500 MHz for ^{1}H, 126 MHz for ^{13}C, 203 MHz for ^{31}P) spectrometer. 1D ^{13}C and ^{31}P spectra were recorded with proton decoupling. ^{1}H and ^{13}C chemical shifts (δ) are reported in parts per million (ppm) relative to residual solvent peaks: $CDCl_3$ (δ_H = 7.26 ppm, δ_C = 77.2 ppm), DMSO-d_6 (δ_H = 2.50 ppm, δ_C = 39.5 ppm), D_2O (δ_H = 4.79 ppm). Coupling constants (*J*) for NMR signals were reported in hertz (Hz). The following abbreviations were used to report multiplet types: s, singlet; d, doublet; t, triplet; q, quadruplet; p, pentet; dd, doublet of doublets; dt, doublet of triplets; dq, doublet of quadruplets; td, triplet of doublets; ddd, doublet of a doublet of a doublet; m, multiplet. High resolution mass spectrometry (HRMS) was performed by direct infusion of compound solutions into a Bruker maXis impact qTOF mass spectrometer or a Thermo Scientific Exactive Plus orbitrap mass spectrometer via ESI or atmospheric-pressure chemical ionization (APCI).

Mammalian cell culture

HeLa X1/5 cells (gift from Prof. Hanadi Sleiman) and HepG2 cells (gift from Prof. Maureen McKeague) were cultured in high-glucose Dulbecco's Modified Eagle Medium (DMEM) supplemented with 10% FBS, 1 × MEM non-essential amino acids, 100 U/mL penicillin, and 100 µg/mL streptomycin at 37°C with 5% CO_2. Cells were passaged at 80–90% confluency with 0.25% Trypsin-EDTA. Cell counting was conducted with a DeNovix CellDrop cell counter after staining with trypan blue.

Oligonucleotide synthesis and analysis

Oligonucleotide synthesis was performed with standard phosphoramidite chemistry on a K&A S4-LC DNA synthesizer at 2 µmol scale. All 2'-F, 2'-OMe, and DNA phosphoramidites (with standard protecting groups) and universal CPG support (500 Å) were purchased from Chemgenes. The VdU phosphoramidite was synthesized according to a previous publication.[2] All phosphoramidites were dissolved in anhydrous MeCN at 0.1 M immediately before synthesis. After synthesis, the DMTr-on oligonucleotides were cleaved and deprotected with ammonium hydroxide at 55°C for 17 h, then dried. The crude material was purified by RP-HPLC on an Agilent 1260 preparative HPLC equipped with an Agilent prep C18 column (100 Å, 5 µm, 10 × 50 mm). The product was eluted with 10–35% MeCN in 100 mM triethylammonium acetate (TEAA, pH = 7.0) in H_2O over 13 min, then held at 35% MeCN for 3 min. Fractions containing the DMTr group were pooled, lyophilized, and detritylated

with 400 µL 20% AcOH in water for 1 h. The detritylation mixture was quenched with 100 µL 1 M NaOAc buffer (pH = 5.0), then directly desalted with Glen-Pak desalting column to afford the oligonucleotide product as the sodium salt. The oligonucleotide was quantified by absorbance at 260 nm on a Thermo Fisher NanoDrop One UV spectrometer, with extinction coefficients calculated with the OligoAnalyzer tool from IDT (VdU and VdA were calculated as U and A, respectively), and extinction coefficients for **p2**-reacted oligonucleotides were estimated using the correction factor at 260 nm reported for PINK.[2]

Guide strands modified with **p2** were prepared by reacting each VdU/VdA-containing strand (100 µM) with **p2-HCl** (1 mM) in 100 mM NaOAc buffer (pH = 5.0) with 25% DMSO at 37°C for 1–2 d. The reaction products were purified on a Waters Arc HPLC system equipped with a Waters XBridge BEH C18 column (130 Å, 3.5 µm, 4.6 × 150 mm) and a 2998 photodiode array (PDA) detector. The samples were eluted with 12% MeCN in 0.1 M TEAA in water for 3 min, 12–25% MeCN over 7 min, then 25–30% MeCN over 3 min at 1.6 mL/min with column temperature of 65°C. Fractions containing the desired products were pooled, lyophilized, and co-evaporated with water three times to afford the final products.

Oligonucleotides were analyzed by LC-MS with a Thermo Scientific Ultimate 3000 UHPLC system equipped with an Agilent AdvanceBio Oligonucleotide column (130 Å, 2.7 µm, 2.1 × 50 mm) coupled to a Bruker maXis impact qTOF mass spectrometer. The samples were eluted with 10–40% MeOH in 100 mM HFIP, 5 mM Et_3N in H_2O over 8 min at 0.2 mL/min with a column temperature of 40°C. The MS data were deconvoluted with Bruker DataAnalysis software version 4.1, and monoisotopic masses are reported in Table S1.

HPLC analysis of tetrazine reactions with different siRNA, DNA duplexes, and VdU nucleoside

siRNA or DNA duplexes were prepared by annealing equal amounts of guide and passenger strands in 1× PBS by heating to 95°C, held for 10 min, then cooled to 4°C at a cooling rate of 0.1°C/s. See Table S2 for duplex names. The annealed duplexes (final 10 µM) were mixed with tetrazines (final 100 µM) in PBS with 10% DMSO and incubated at 37°C for 6 h prior to RP-HPLC analysis. Nucleoside reactions were performed by mixing VdU (final 20 µM) with each tetrazine (final 100 µM), and thymidine was added as an internal standard (final 20 µM) in PBS with 10% DMSO and incubated at 37°C for 24 h prior to RP-HPLC analysis.

RP-HPLC analyses were performed on a Waters Arc HPLC system with a Waters XBridge Premier Oligonucleotide BEH C18 column (300 Å, 2.5 µm, 4.6 × 150 mm) and 2998 photodiode array (PDA) detector. siRNA reaction samples were analyzed using a gradient of 11.5–15% MeCN in 0.1 M TEAA over 4.5 min, followed by 15–20% MeCN over 3.5 min at 2.4 mL/min and 65°C. DNA duplex samples were analyzed using a gradient of 7–10% MeCN in 0.1 M TEAA over 4 min, followed by 10–25% MeCN over 3.5 min under the same conditions. Nucleoside reaction samples were analyzed using a gradient of 1–30% MeCN in 0.1% TFA over 9 min at 1.6 mL/min at room temperature. Chromatograms were monitored at 260 nm for oligonucleotides and 270 nm for nucleosides, while full absorbance spectra were collected to identify excess tetrazines. Guide strand conversion was quantified using the passenger strand as an internal standard. The conversion of VdU nucleoside was calculated using thymidine as an internal standard.

Viscosity measurements of calf-thymus DNA (CTDNA) solutions

CTDNA (solid fibers) was dissolved in PBS at ~8 mg/mL by gentle rotation overnight. The solution was divided into 100 µL aliquots in 600 µL microcentrifuge tubes and sonicated using a QSonica Q800R3 sonicator at 25% power, 10 kHz for 40 s at 4°C. The samples were then combined, diluted 1.5-fold, and gently rotated overnight, followed by centrifugation at 10 000 × g for 1 min. The supernatant was collected for viscosity measurements. DNA concentration was determined using a Thermo Fisher NanoDrop One UV spectrometer and converted to base-pair concentration using an average molecular weight of 660 g/mol per base pair.

For viscosity measurements, CTDNA (final concentration: 300 µM base pairs) was incubated with tetrazines in PBS containing 1% DMSO for >30 min prior to analysis (**tz6** and **tz8**: 10–50 µM; **p2**: 5–50 µM). Sample viscosities were measured using a Cannon–Manning semi-micro Ostwald viscometer (size 75) immersed in a water bath at room temperature (21.8–22.2°C). Flow times (125.98–177.13 s) were recorded using a smartphone stopwatch application, with three replicates measured for each sample. Relative DNA length (L/L_0) was calculated from the cube root of the intrinsic viscosities of tetrazine-treated ([η]) and untreated ([η_0]) samples, according to literature methods.[3]

Resazurin assays

HeLa X1/5 cells were seeded at 5000 cells/well in 96-well plates and incubated overnight before replacing the medium with DMEM containing different concentrations of tetrazines. Cells were incubated with tetrazines for 4 days, then were incubated with DMEM containing 44 µM resazurin for 1–2 h at 37°C. The fluorescence from the reduced resorufin product was measured on a plate reader (Agilent Synergy H1, Ex: 554/18 nm; Em: 593/20 nm). The fluorescence intensity was normalized to the sample without tetrazine treatment, plotted against tetrazine concentration and fitted with an inhibitor response curve. The IC_{50} values were calculated from the means of triplicate measurements.

Neo-BODIPY displacement assays

Neo-BODIPY (Neomycin B-BODIPY conjugate) was synthesized as previously described.[4] Displacement assays were performed by mixing Neo-BODIPY (final 25 nM) and RNA duplex **d1.6** and **d4.0** (final 25 nM) with varying concentrations of tetrazines (10–250 µM for **tz6**, 50–1000 µM for **tz8**) in PBS containing 10% DMSO for 30 min at room temperature in a black 384-well plate, followed by fluorescence anisotropy measurements on a plate reader (Agilent Synergy H1) using the green FP cube. Anisotropy values were normalized with samples containing only Neo-BODIPY (as 0) and samples with Neo-BODIPY and RNA duplex (as 1).

Comparison of siRNA activities before and after reaction with p2

HeLa X1/5 cells were seeded into 24-well plates (25000 cells/well, three wells per siRNA) and incubated overnight before being transfected with different siRNA duplexes (2 nM final concentration) with Lipofectamine RNAiMAX according to manufacturer's protocol. The cells were lysed 24 h post-transfection and the lysate was subjected to luciferase assays (One-Glo EX Luciferase Assay System, Promega) in duplicates on 96-well white and transparent plates, respectively. The luminescence of each sample was normalized to samples transfected with scramble

control siRNA (**d0.2**).

siRNA deactivation assays with Luciferase-targeting siRNA

As long-term culture (>4 days) of HeLa X1/5 cells in 24-well plates was not possible due to their rapid growth, we performed the siRNA and tetrazine treatment in two separate plates. HeLa X1/5 cells were first seeded into 6-well plates (300000 cells/well, 1 well per siRNA) and incubated overnight before being transfected with different siRNA duplexes at a final concentration of 2 nM with Lipofectamine RNAiMAX according to manufacturer's protocol. After incubation with siRNA for 18 h, the cells were trypsinized and reseeded into 24-well plates (15000 cells/well, 3 wells per treatment) containing 1 mL DMEM with different tetrazines (0.75 µM for **p2**, 7.5 µM for **tz6** and **tz8**). The cells were lysed after 4-day incubation with tetrazines, and the lysate was subjected to luciferase assays (One-Glo EX Luciferase Assay System, Promega) and BCA protein quantification assays (Pierce BCA Assay Kit, ThermoFisher) in duplicates on 96-well white and transparent plates, respectively. The luminescence of each sample was first corrected with protein concentrations determined by the BCA assay, then normalized to samples transfected with scramble control siRNA (**d0.2**) treated with the same tetrazine. Groups with and without tetrazine treatment under same siRNA treatment were compared with Student's *t*-test.

siRNA deactivation assays with AGT-targeting siRNAs

HepG2 cells were reversed-transfected with different siRNA duplexes (2 nM final concentration) using Lipofectamine RNAiMAX according to manufacturer's protocol in 24-well plates (25000 cells/well, three wells per treatment). After incubating for 18 h, the medium was replaced with 1 mL DMEM containing 7.5 µM of **tz6** and mRNA expression analyses were conducted after incubating cells with tetrazines for 7 days (fresh medium and tetrazines were replaced on the 4th day).

mRNA quantities were analyzed by RT-qPCR. Total RNA from cells was extracted with EZ-10 DNAaway RNA Mini-Prep Kit (Bio Basic), followed by cDNA synthesis (~500 ng total RNA input in 10-µL reactions) with GoScript Reverse Transcriptase Oligo (dT) kit (Promega). The cDNA product was diluted to 100 µL with nuclease-free water and used for qPCR in 20-µL reactions with Bio-Rad SsoAdvanced Universal Probes Supermix (triplicate reactions per target per sample, 5 µL cDNA solution per reaction) in clear 96-well plates with commercially available TaqMan assays (*AGT*: Thermo Fisher #Hs01586213_m, *HPRT1*: Bio-Rad #qHsaCIP0030549, *PPIA*: Biorad #qHsaCEP0041342, all FAM-labeled). The qPCR reactions were performed on a Bio-Rad CFX Opus 96 system using cycle parameters recommended by the qPCR mix manufacturer. *AGT* expression levels were obtained using the $\Delta\Delta C_t$ method with *HPRT1* and *PPIA* as reference genes and then normalized to cells treated with scramble control siRNA and the same tetrazine as the samples. Groups with and without tetrazine treatment under same siRNA treatment were compared with Student's *t*-test. The three siRNA groups without tetrazine treatment were compared with one-way ANOVA test.

Supplementary Tables

Table S1 Oligonucleotides used in this study. (See Figure S15-S21 for LC-MS analysis of **p2** modified guide strands)

Name	Sequence (5'-3')	Calculated mass	Found mass
G1	U'U'AAUUAAAGACUUCAAGCGG	6920.1117	6920.0877
G1-VdU2	U'U_V'AAUUAAAGACUUCAAGCGG	6928.1368	6928.1143
G1-VdU5	U'U'AAU_VUAAAGACUUCAAGCGG	6916.1168	6916.1064
G1-VdU6	U'U'AAUU_VAAAGACUUCAAGCGG	6928.1368	6928.1288
G1-VdU14	U'U'AAUUAAAGACUU_VCAAGCGG	6928.1368	6928.1467
G1-VdU1314	U'U'AAUUAAAGAC$U_V$$U_V$CAAGCGG	6924.1418	6924.1417
G1-VdU2-**p2**	U'U_V^P'AAUUAAAGACUUCAAGCGG	7269.3128 (+H$^+$)	7269.3224
G1-VdU5-**p2**	U'U'AAU_V^PUAAAGACUUCAAGCGG	7257.2976 (+H$^+$)	7257.2976
G1-VdU6-**p2**	U'U'AAUU_V^PAAAGACUUCAAGCGG	7269.3128 (+H$^+$)	7269.3245
G1-VdU14-**p2**	U'U'AAUUAAAGACUU_V^PCAAGCGG	7269.3128 (+H$^+$)	7269.3150
G1-VdA3	U'U'A_VAUUAAAGACUUCAAGCGG	6915.1215	6915.0496
G1-VdA4	U'U'AA_VUUAAAGACUUCAAGCGG	6915.1215	6915.0449
G1-VdA7	U'U'AAUUA_VAAGACUUCAAGCGG	6915.1215	6915.0423
G1-VdA3-**p2**	U'U'A_V^PAUUAAAGACUUCAAGCGG	7258.3012	7258.0245
G1-VdA4-**p2**	U'U'AA_V^PUUAAAGACUUCAAGCGG	7258.3012	7258.2625
G1-VdA7-**p2**	U'U'AAUUA_V^PAAGACUUCAAGCGG	7258.3012	7258.0500
G0	U'U'UACGAUUUAAAUAUUGCGG	6923.1212	6923.1350
G5	U'G'UACUCUCAUUGUGGAUGAC'G'A	7600.1465	7600.0021
G5-VdU14	U'G'UACUCUCAUUGU_VGGAUGAC'G'A	7608.1716	7608.1946
P1	G'C'UUGAAGUCUUUAAUUAATT	6864.0504	6863.9955
P1-A	G'C'UUGAUGUCUUAAAUUAATT	6887.0777	6887.0769
P2-C3	G'C'UUGAAGUCUUSAAUUAATT	6682.0177	6682.0000
P4-C3	G'C'UUGAAGUCUUUSAAUUAATT	7002.0586	7002.0510
P4-C3-14	G'C'UUGASAGUCUUUAAUUAATT	7002.0586	7002.0720
P5	G'U'CAUCCACAAUGAGAGUACA	6978.1648	6978.1384
P5-C3 bulge	G'U'CAUCCASCAAUGAGAGUACA	7116.1730	7116.1252
P0	G'C'AAUAUUUAAAUCGUAAATT	6942.1899	6942.2041
DNA1	GCCGCAATAU_VATAACGCCG	5791.0163	5790.9373
DNA2	CGGCGTTATATATTGCGGC	5831.9939	5831.9084

U_V: VdU, A_V: VdA, U_V^P: VdU-**p2**, A_V^P: VdA-**p2** S: C3-spacer, ': phosphorothioate linkage

Sugar modifications: Black: 2'-OMe, Green: 2'-F, Blue: 2'-deoxy, Orange: (*S*)-GNA

Table S2 Duplexes used in this study.

Duplex name	Guide strand	Passenger strand	Designs	Target
**d0.2**	G0	P0	scramble control	scramble control
**d1.1**	G1-VdU6	P1-A	mismatch design with VdU	LUC
**d1.4**	G1-VdU6	P1	perfect duplex with VdU at g6	
**d1.6**	G1	P1	perfect duplex	
**d1.8**	G1-VdU14	P1	perfect duplex with VdU at g14	
**d1.10**	G1-VdU2	P1	perfect duplex with VdU at g2	
**d1.11**	G1-VdU5	P1	perfect duplex with VdU at g5	
**d1.12**	G1-VdA3	P1	perfect duplex with VdA at g3	
**d1.13**	G1-VdA4	P1	perfect duplex with VdA at g4	
**d1.15**	G1-VdA7	P1	perfect duplex with VdA at g7	
**d2.1**	G1-VdU6	P2-C3	abasic site design with VdU	
**d4.0**	G1	P4-C3	bulge design	
**d4.1**	G1-VdU6	P4-C3	bulge design with VdU	
**d4.11**	G1-VdU14	P4-C3-14	bulge design with VdU at g14	
**d4.12**	G1-VdU1314	P4-C3-14	bulge design with VdU at g13 and g14	
**d5.1**	G5	P5	original Zilebesiran sequence without GalNAc	AGT
**d5.2**	G5-VdU14	P5	perfect duplex with VdU at g14	
**d5.3**	G5-VdU14	P5-C3 bulge	bulge design with VdU at g14	
**d9.1**	DNA1	DNA2	double-stranded DNA with VdU	-

Supplementary figures and discussions

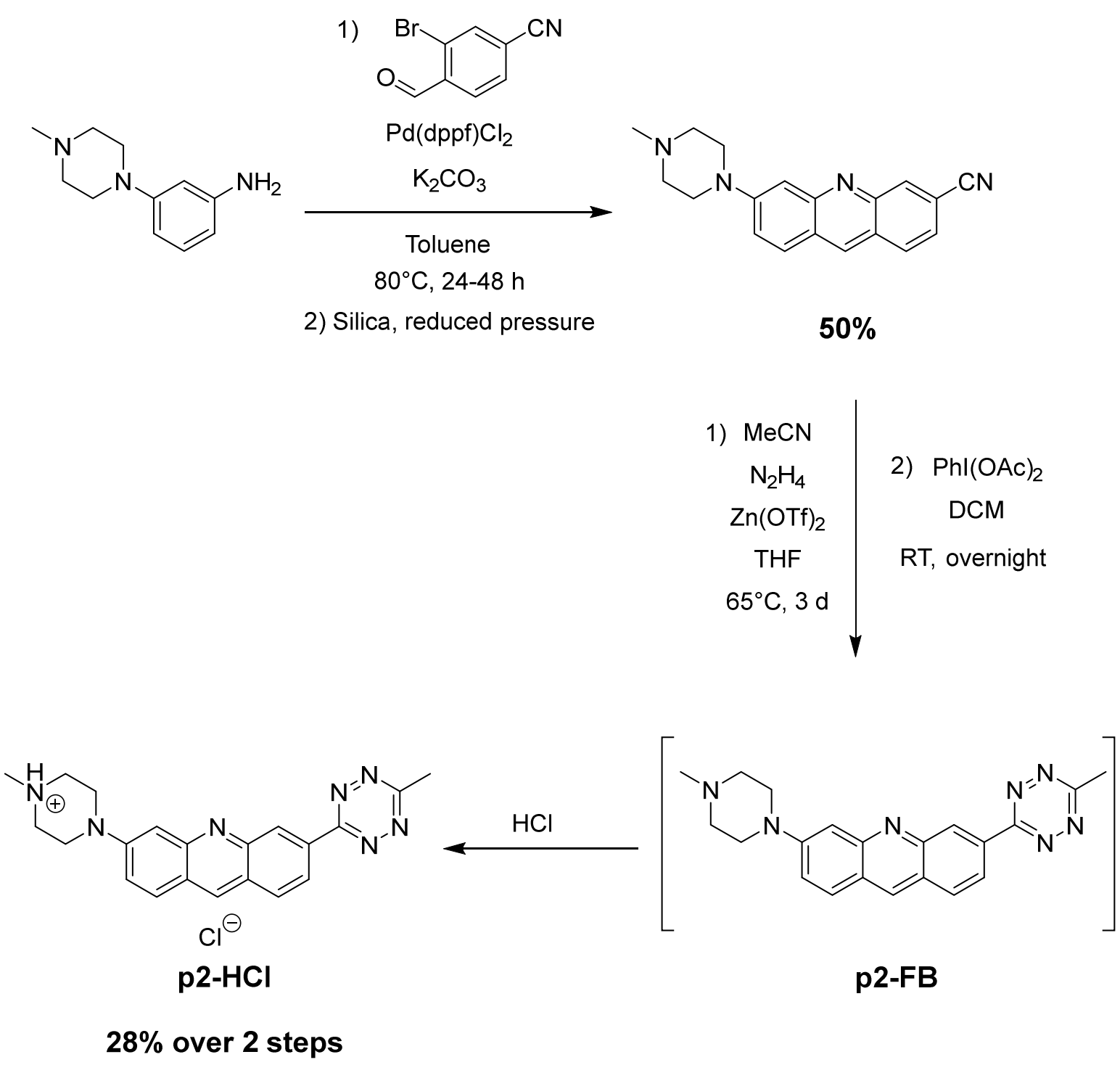

Figure S1 Synthesis of **p2**.

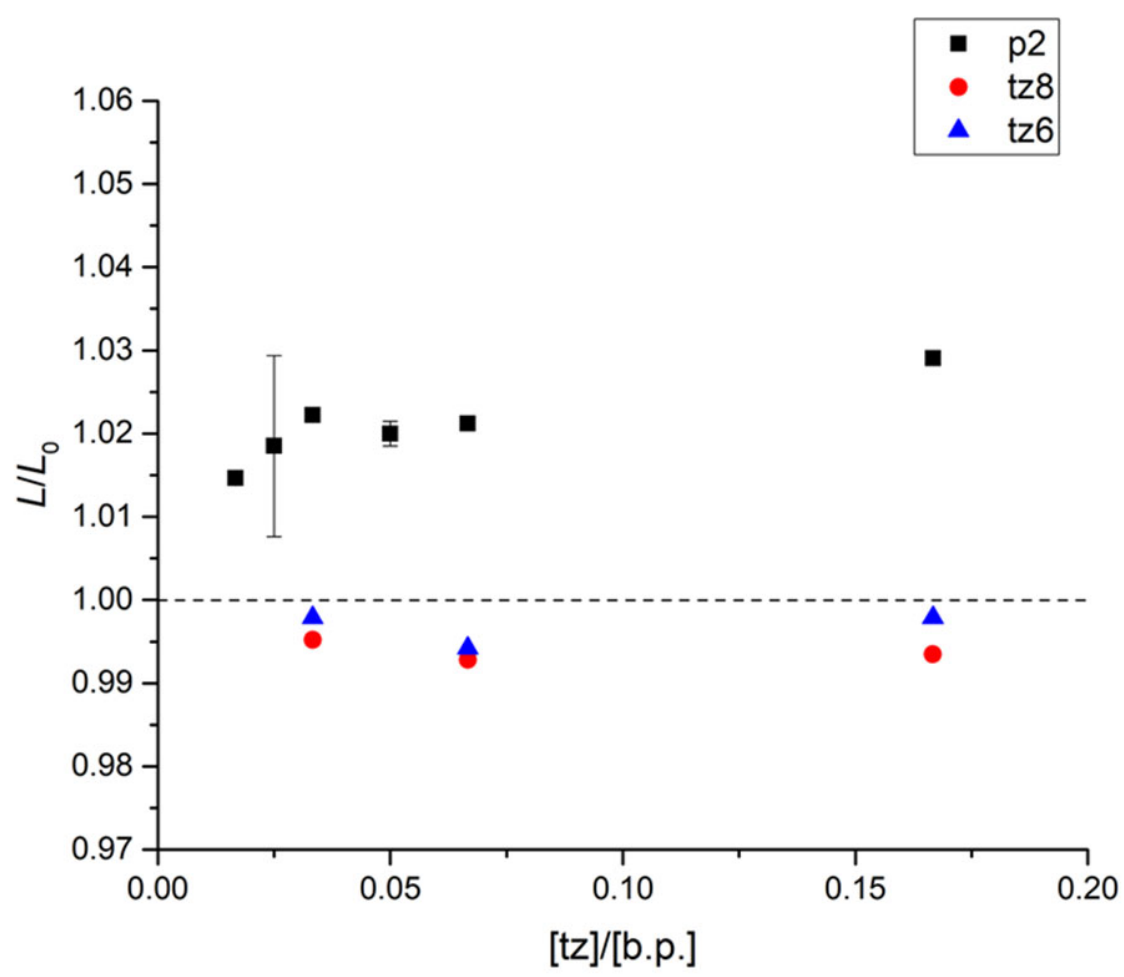

Figure S2 Changes in viscosity of calf thymus DNA (CTDNA, 300 μM base-pairs in PBS, pH = 7.4) solutions upon incubation with different concentrations of tetrazines. Data represent mean ± SD among samples (n = 2 for **p2** samples, n = 1 for **tz6** and **tz8** samples with technical triplicates).

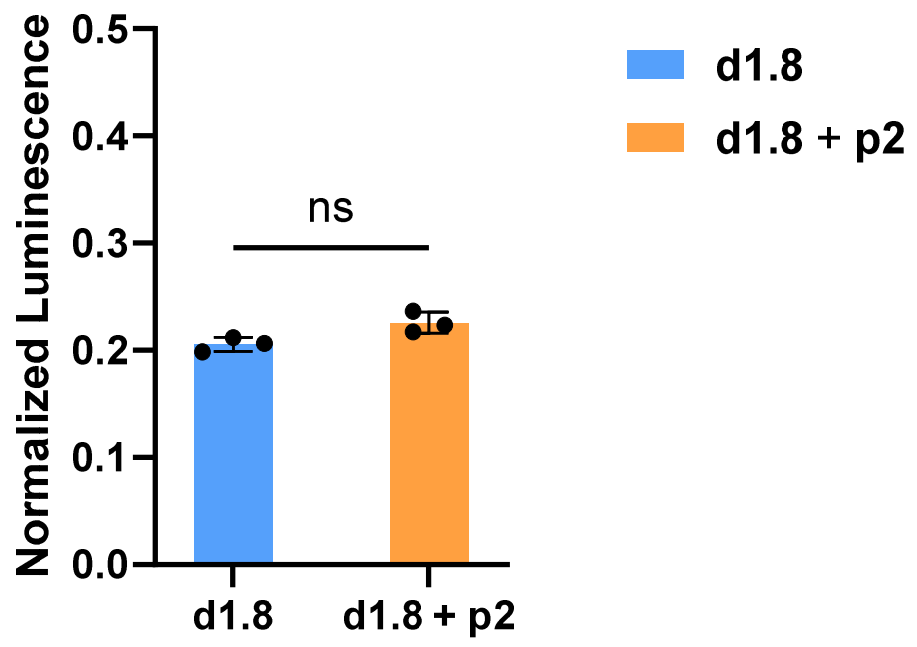

Figure S3 Normalized luminescence signals from HeLa X1/5 cell lysates upon treatment with 2 nM **d1.8** for 18 h followed by 0.75 mM **p2** for 4 days. The luminescence signals of the samples were first corrected to total protein concentrations of each cell lysate and then normalized to samples treated with scrambled siRNA control (**d0.2**). Data represents mean ± SD ($n = 3$). ns, $p > 0.05$ by Student's t-test.

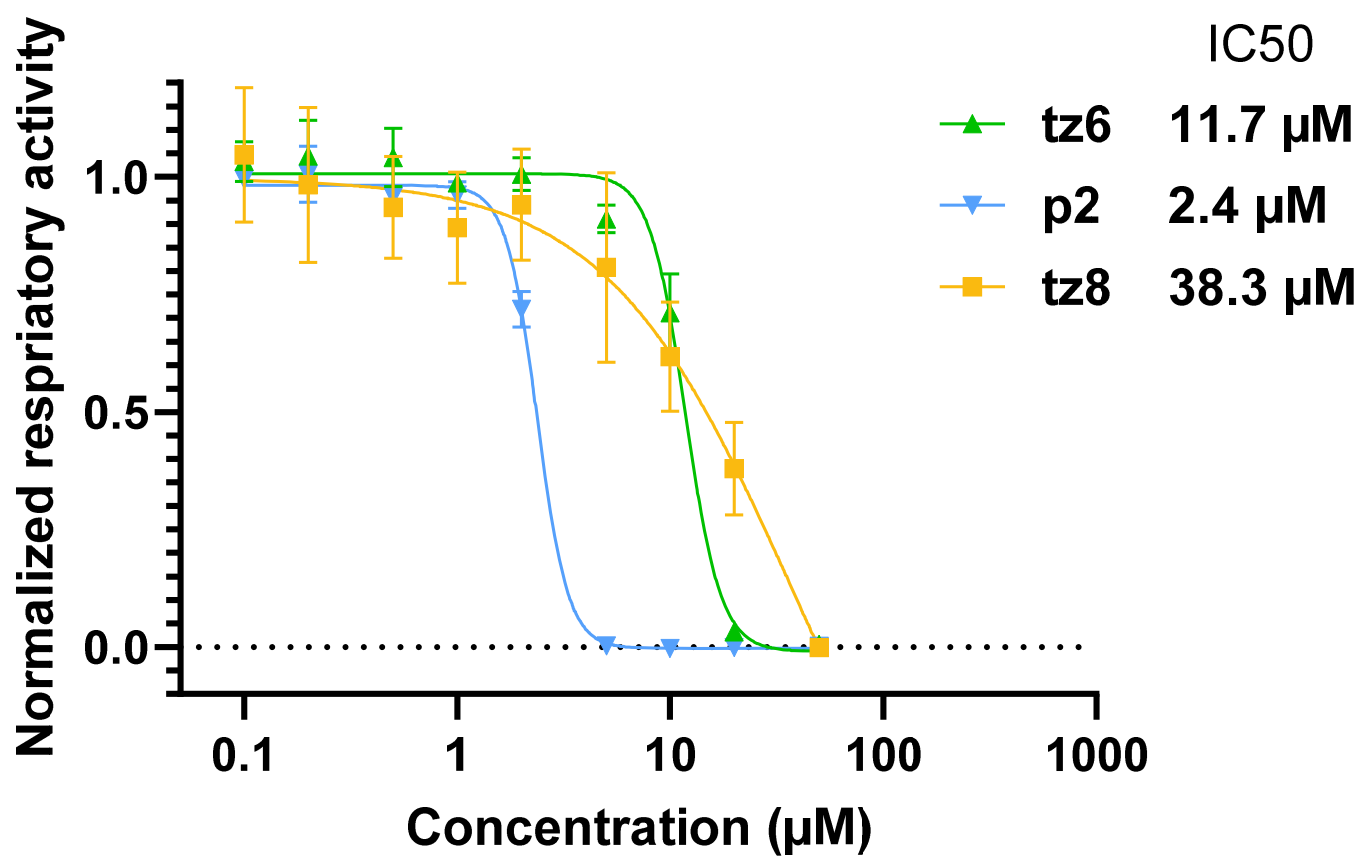

Figure S4 Toxicity of tetrazines in HeLa X1/5 cells after 4-day incubations according to the resazurin assay. Data represent mean ± SD ($n = 3$).

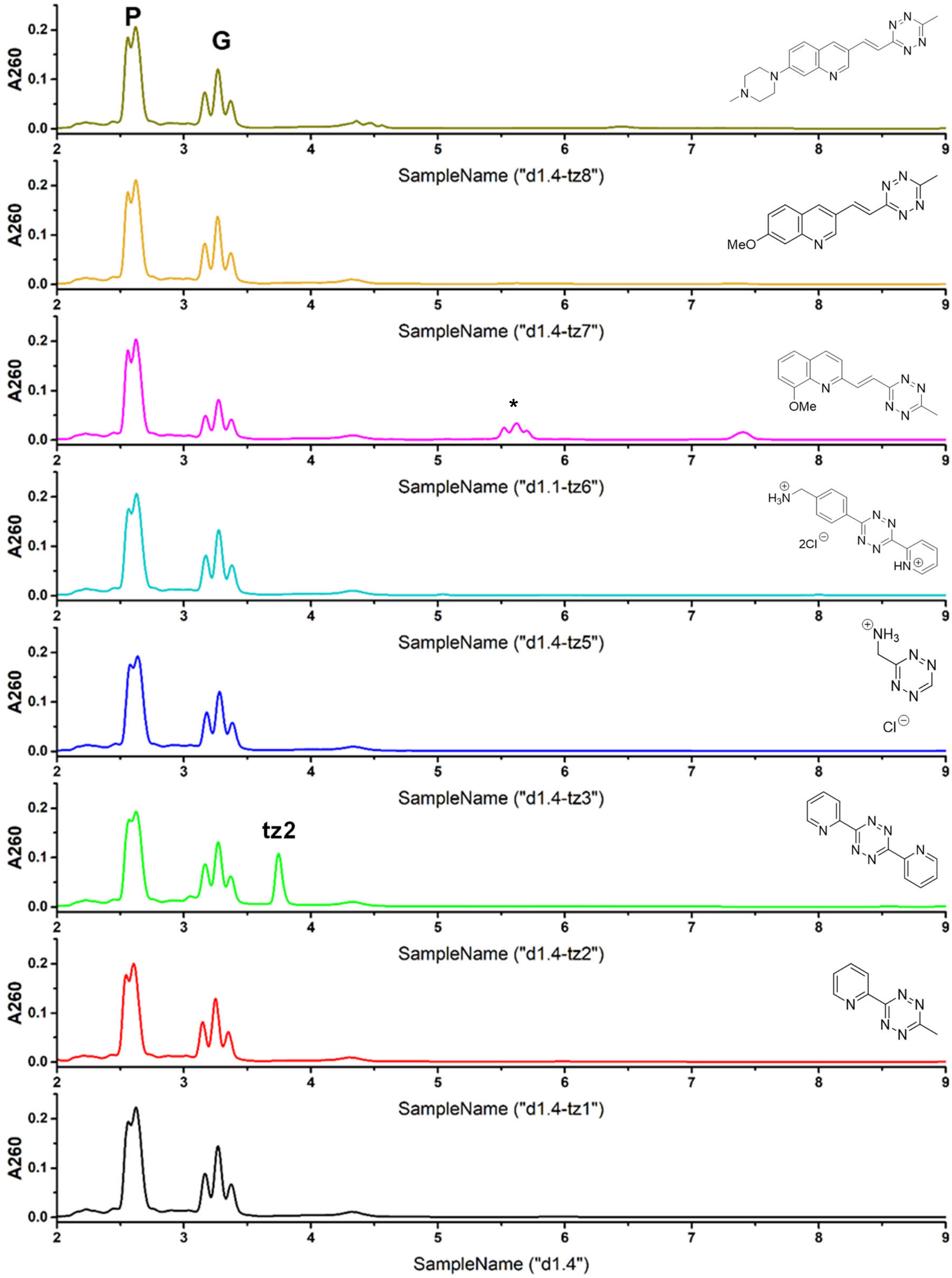

Figure S5 Raw HPLC traces of tetrazine screening reactions (**d1.4** (10 µM) with **tz1**, **tz2**, **tz3**, **tz5**, **tz6**, **tz7**, **tz8** (100 µM) in PBS with 10% DMSO at 37°C for 6 h). Labels in chromatograms showed tentative assignment of HPLC peaks from PDA absorbance spectrum. G: guide strand, P: passenger strand, *, tetrazine adduct of guide strand.

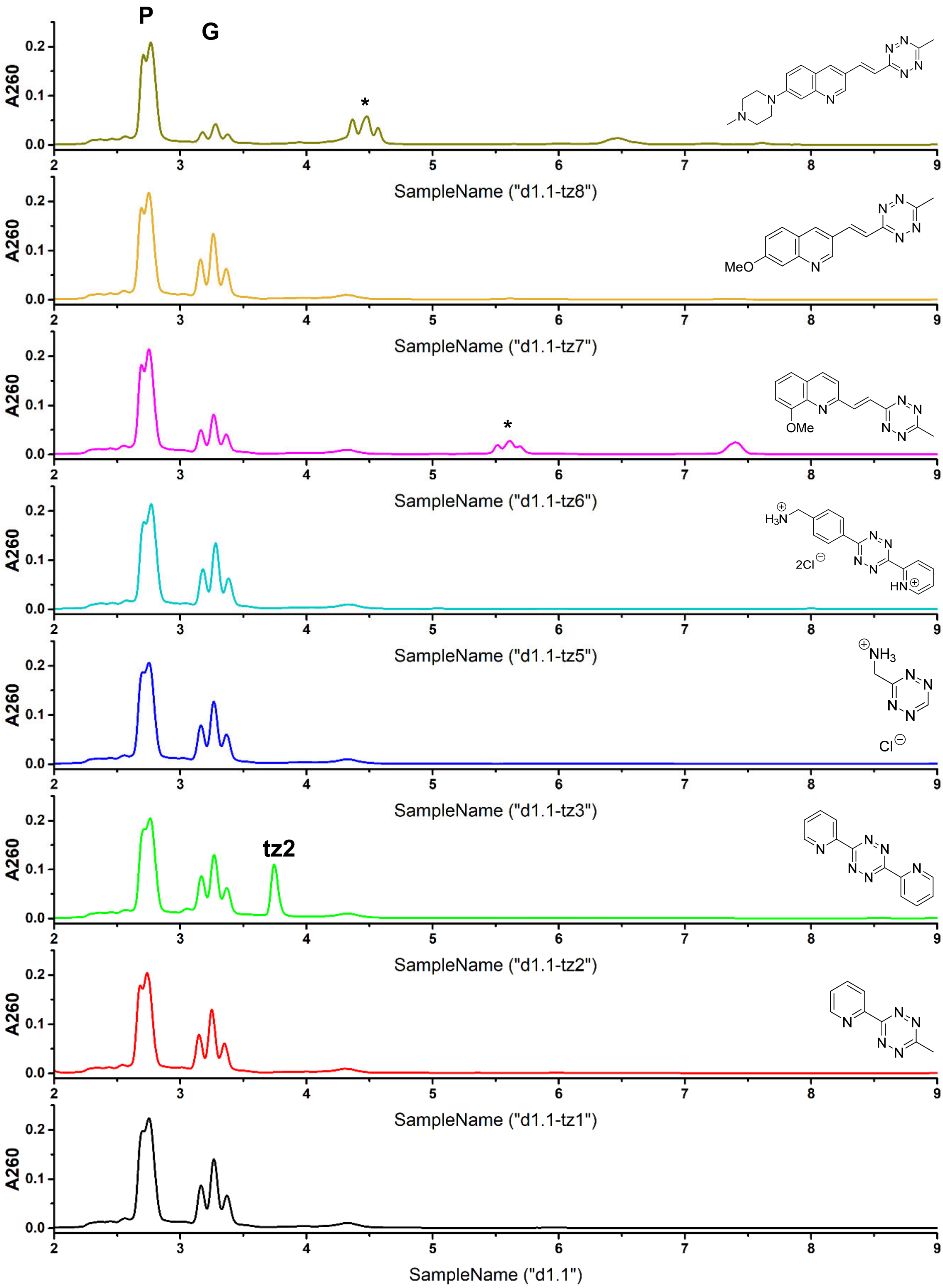

Figure S6 Raw HPLC traces of tetrazine screening reactions (**d1.1** (10 μM) with **tz1**, **tz2**, **tz3**, **tz5**, **tz6**, **tz7**, **tz8** (100 μM) in PBS with 10% DMSO at 37°C for 6 h). Labels in chromatograms showed tentative assignment of HPLC peaks from PDA absorbance spectrum. G: guide strand, P: passenger strand, *, tetrazine adduct of guide strand.

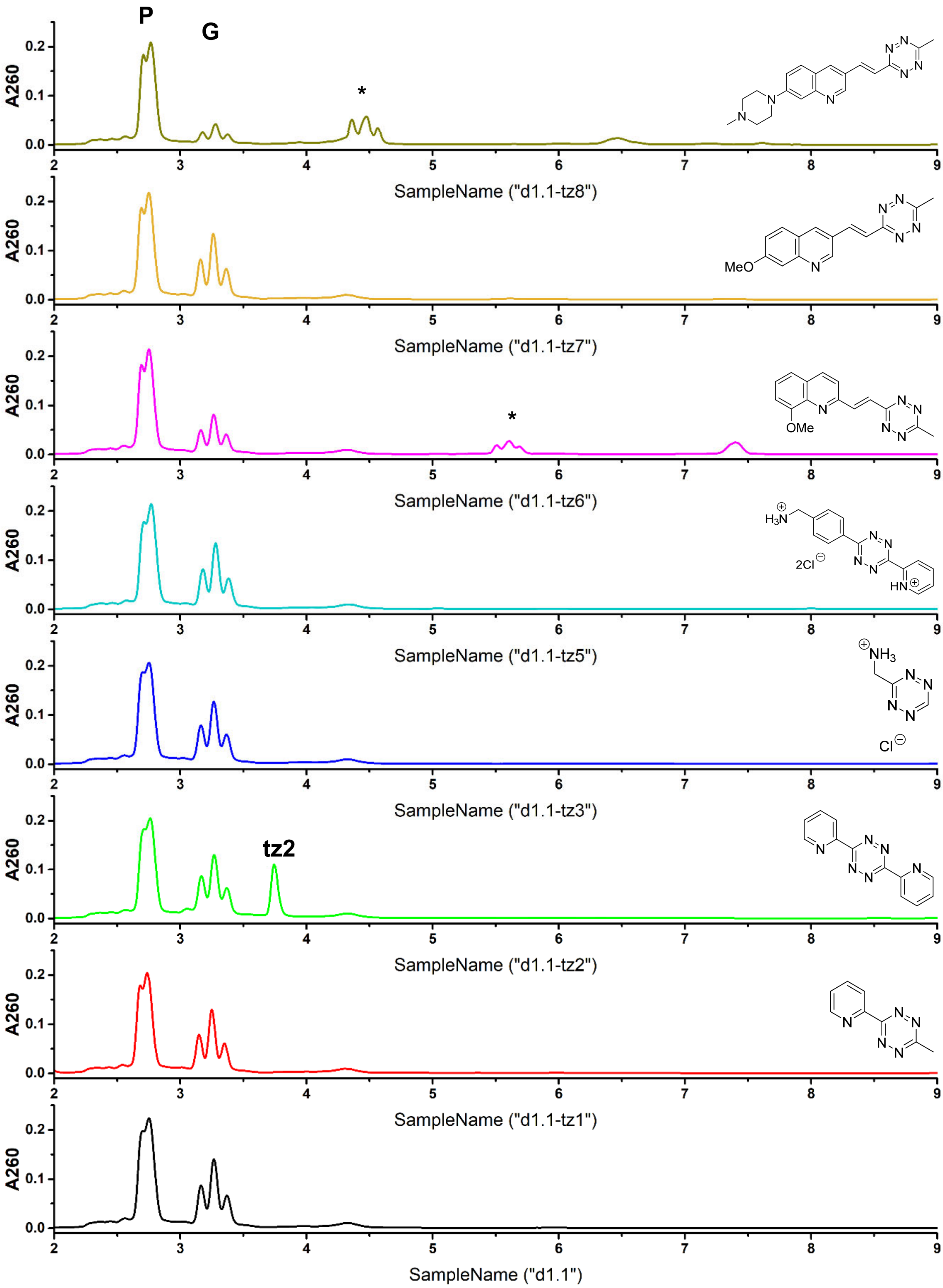

Figure S7 Raw HPLC traces of tetrazine screening reactions (**d2.1** (10 μM) with **tz1, tz2, tz3, tz5, tz6, tz7, tz8** (100 μM) in PBS with 10% DMSO at 37°C for 6 h). Labels in chromatograms showed tentative assignment of HPLC peaks from PDA absorbance spectrum. G: guide strand, P: passenger strand, *, tetrazine adduct of guide strand.

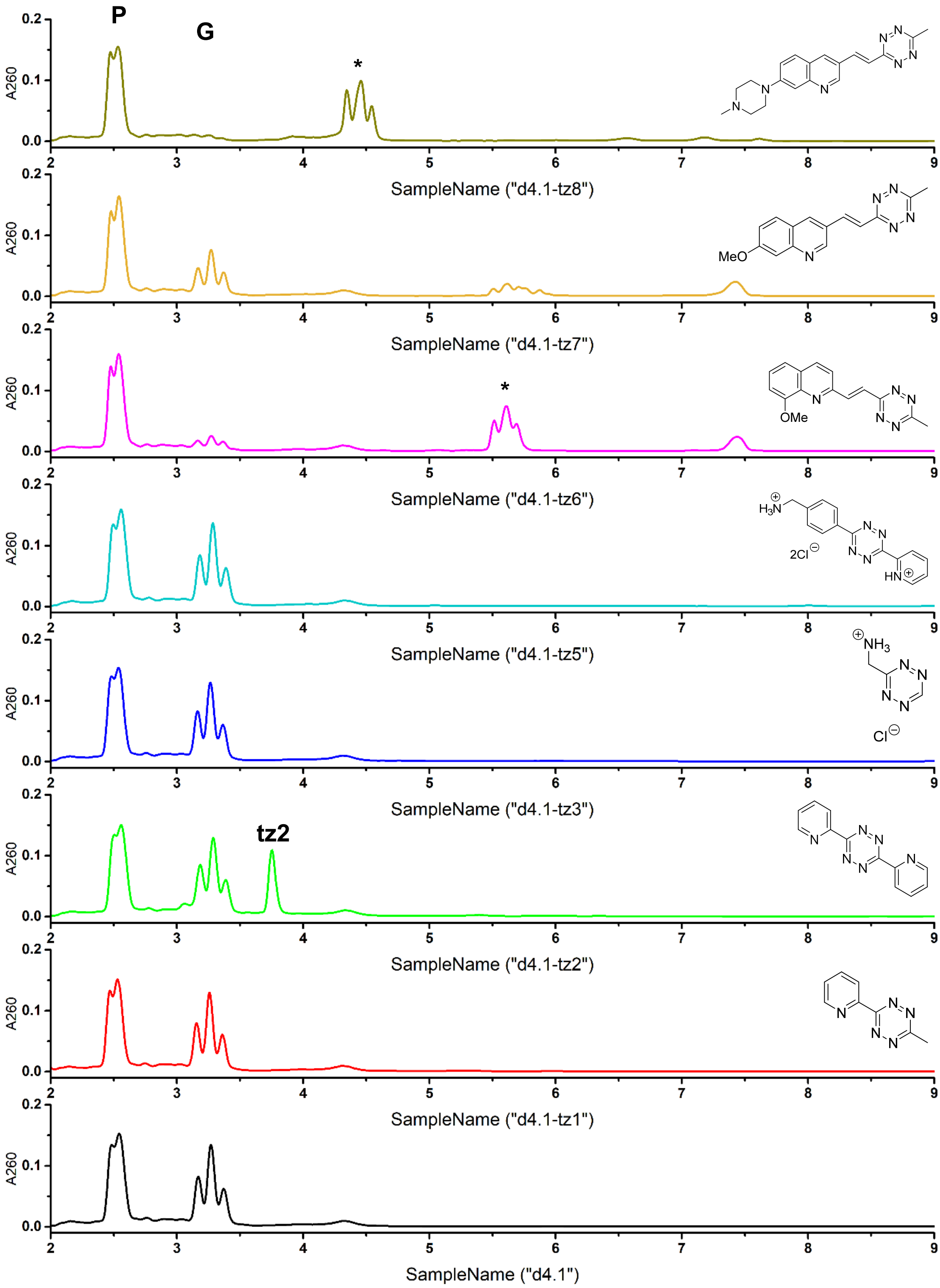

Figure S8 Raw HPLC traces of tetrazine screening reactions (**d4.1** (10 μM) with **tz1, tz2, tz3, tz5, tz6, tz7, tz8**(100 μM) in PBS with 10% DMSO at 37°C for 6 h). Labels in chromatograms showed tentative assignment of HPLC peaks from PDA absorbance spectrum. G: guide strand, P: passenger strand, *, tetrazine adduct of guide strand.

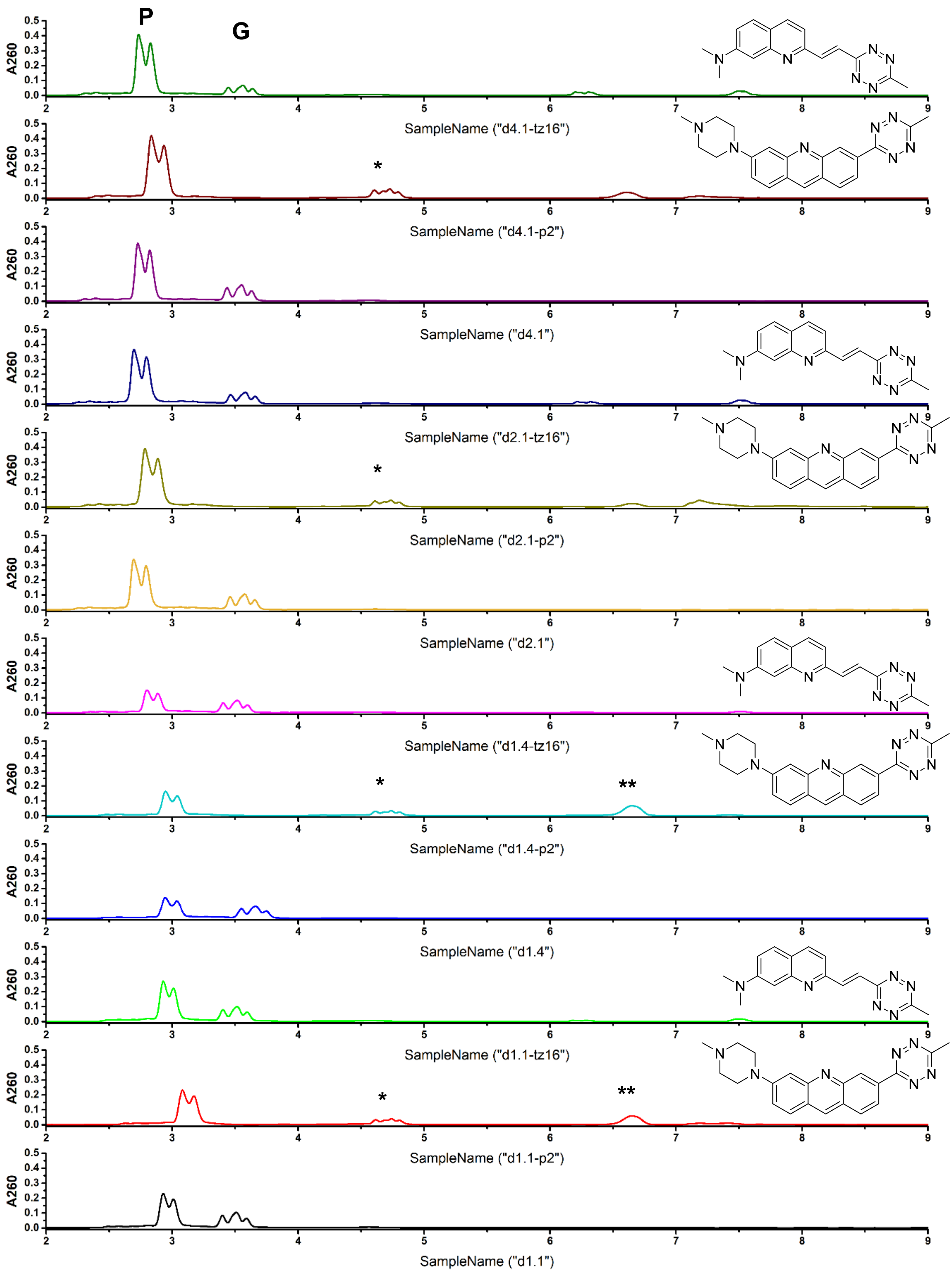

Figure S9 Raw HPLC traces of tetrazine screening reactions (duplexes (10 μM) with **tz16** and **p2** (100 μM) in PBS with 10% DMSO at 37°C for 6 h). G: guide strand, P: passenger strand. Labels in chromatograms showed tentative assignment of HPLC peaks from PDA absorbance spectrum. G: guide strand, P: passenger strand, *, **p2** single adduct of guide strand, **, **p2** double adduct of guide strand.

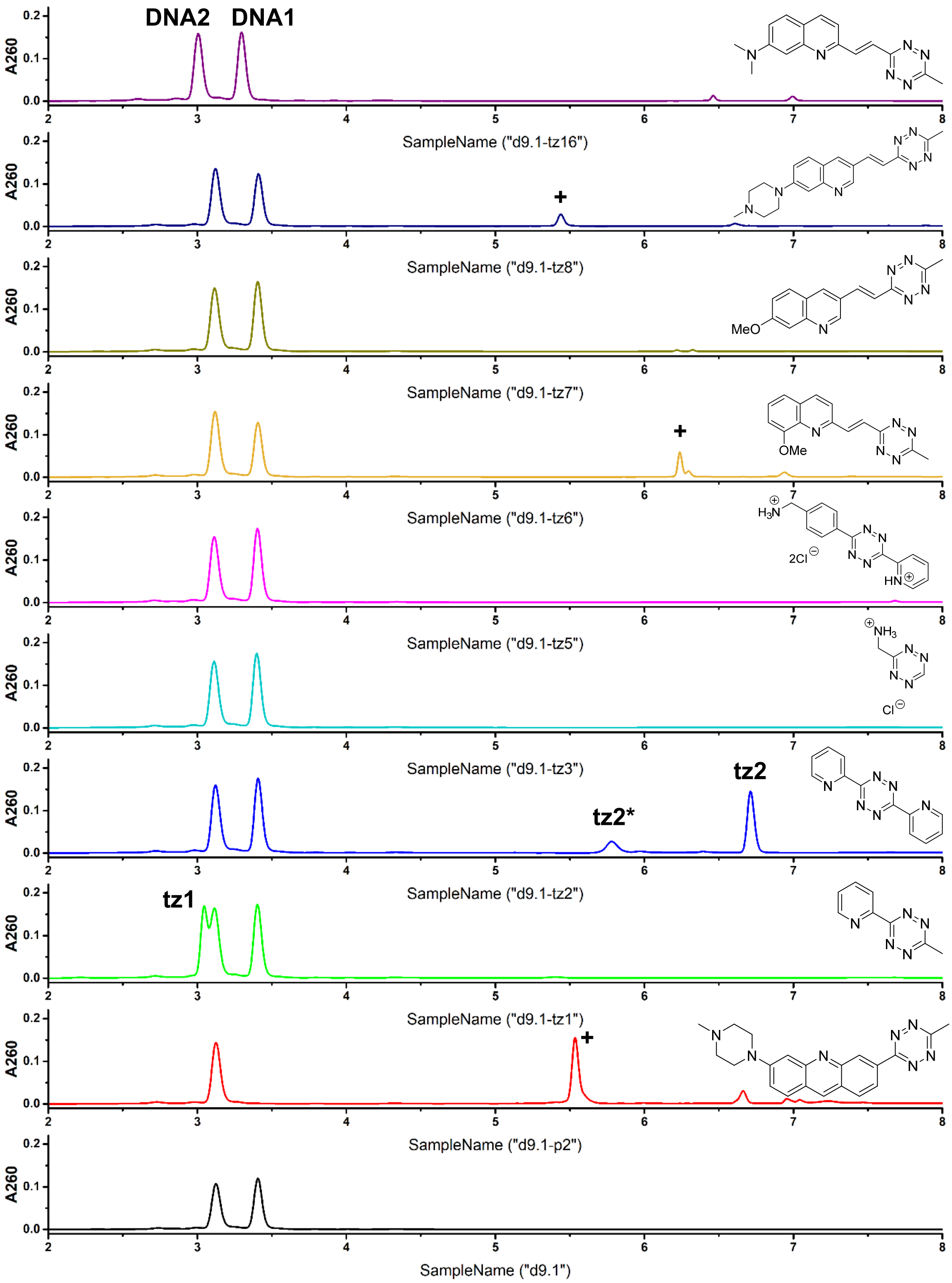

Figure S10 Raw HPLC traces of tetrazine screening reactions (**d9.1** (10 μM) with tetrazines (100 μM) in PBS with 10% DMSO at 37°C for 6 h). Labels in the chromatogram showed tentative assignment of HPLC peaks from PDA absorbance spectrum. +, tetrazine single adduct of DNA1, **tz2***, degraded **tz2**.

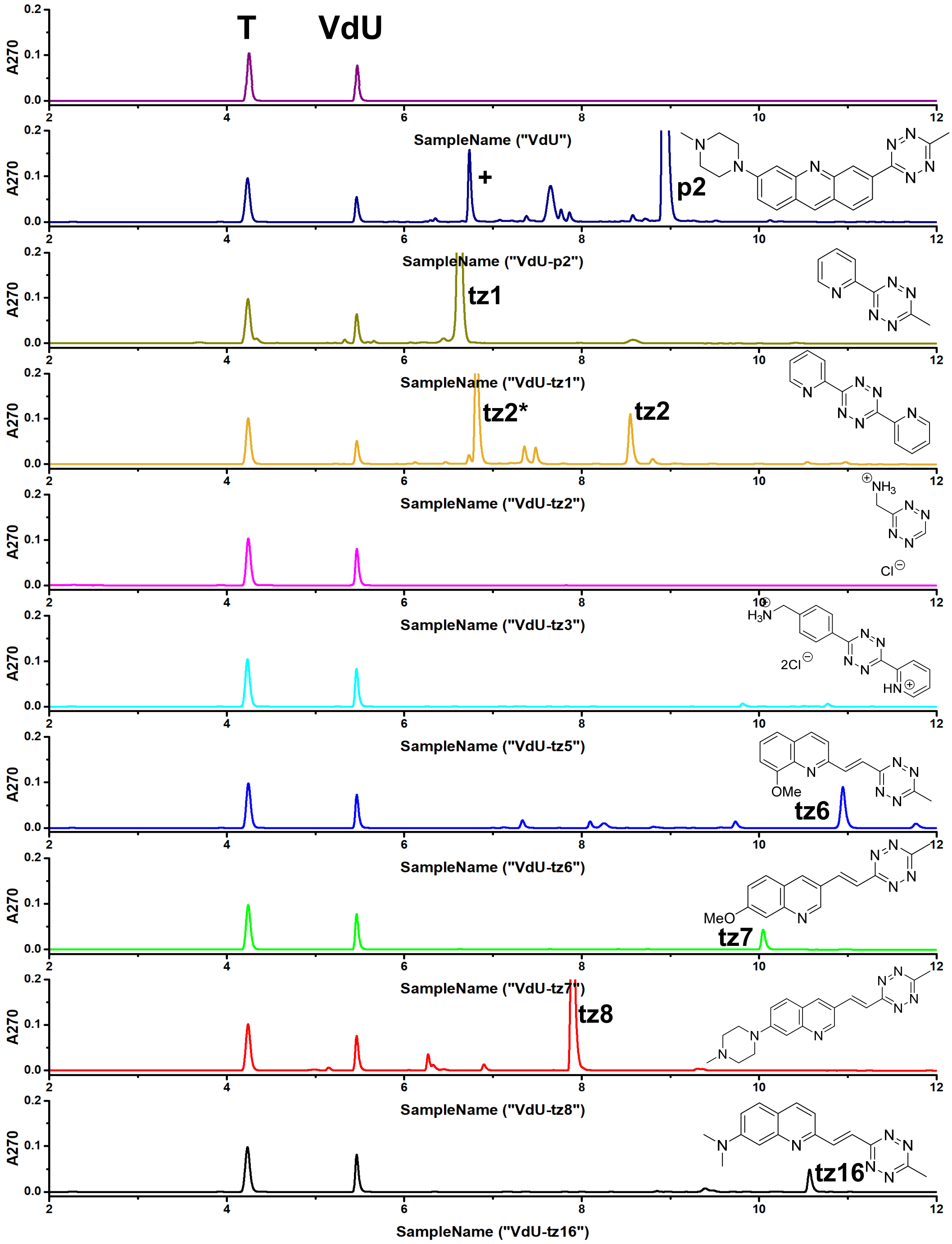

Figure S11 Raw HPLC traces of VdU (20 µM) react with tetrazines (100 µM) in PBS with 10% DMSO at 37°C for 24 h. Labels in the chromatogram showed tentative assignment of HPLC peaks from PDA absorbance spectrum. **tz2***, degradation product of **tz2**; +, VdU-**p2**.

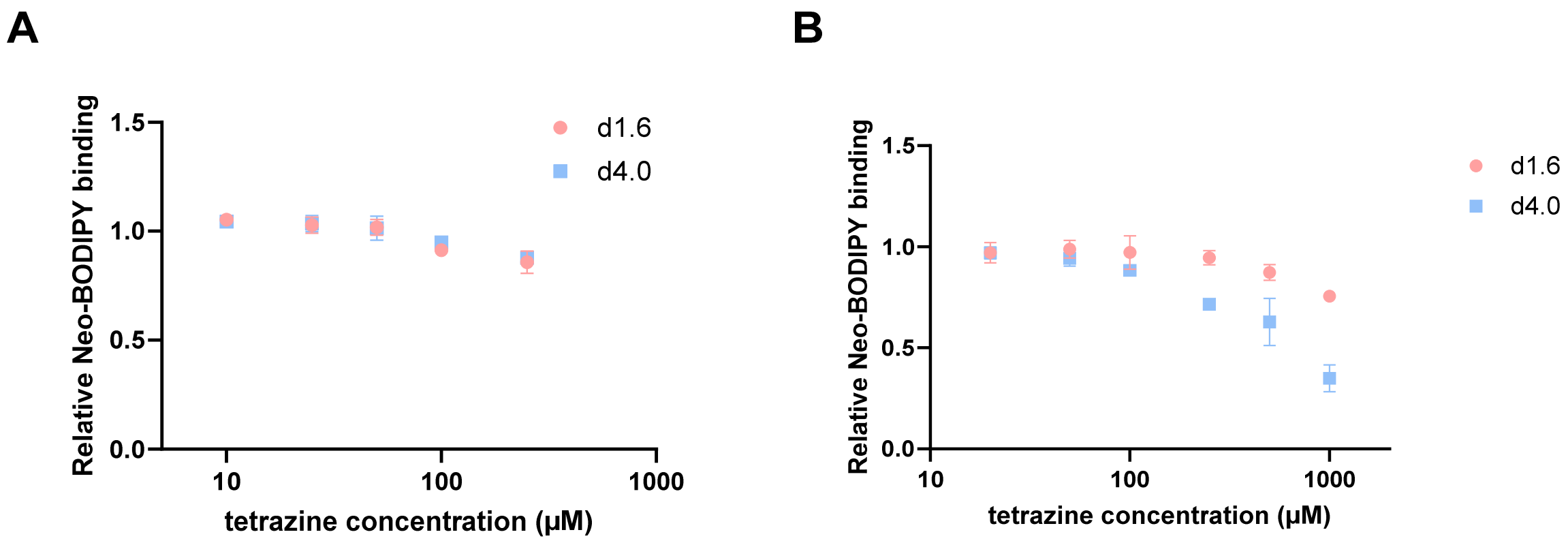

Figure S12 Displacement of Neo-BODIPY from siRNA duplexes by **tz6** (A) and **tz8** (B) in 1×PBS containing 10% DMSO. Data represent mean ± SD (*n* = 3).

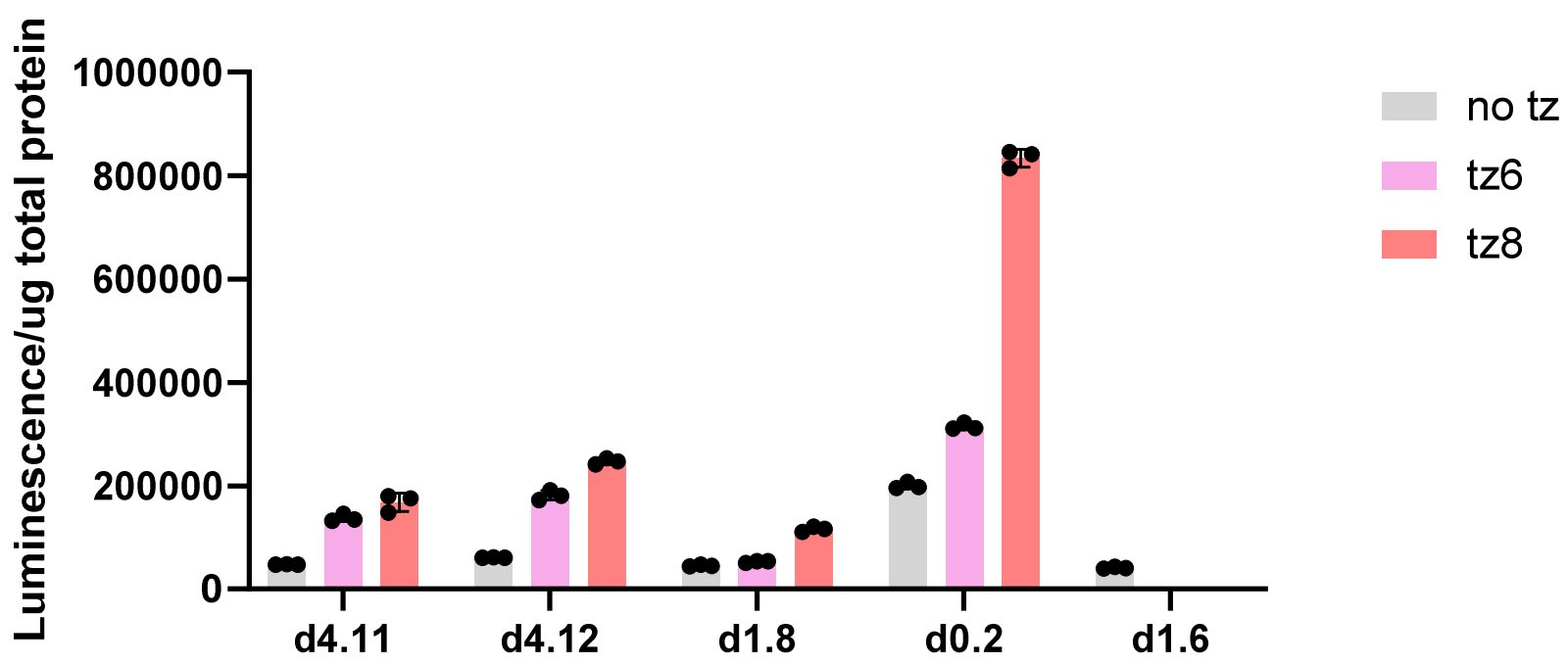

Figure S13 Luminescence data from siRNA deactivation assays with **tz6** and **tz8** (with HeLa X1/5 cells, treated with siRNA, 2 nM for 18 h, then tetrazines, 7.5 µM for 4 days), corrected only by protein concentration. Mean ± SD (*n* = 3) was plotted.

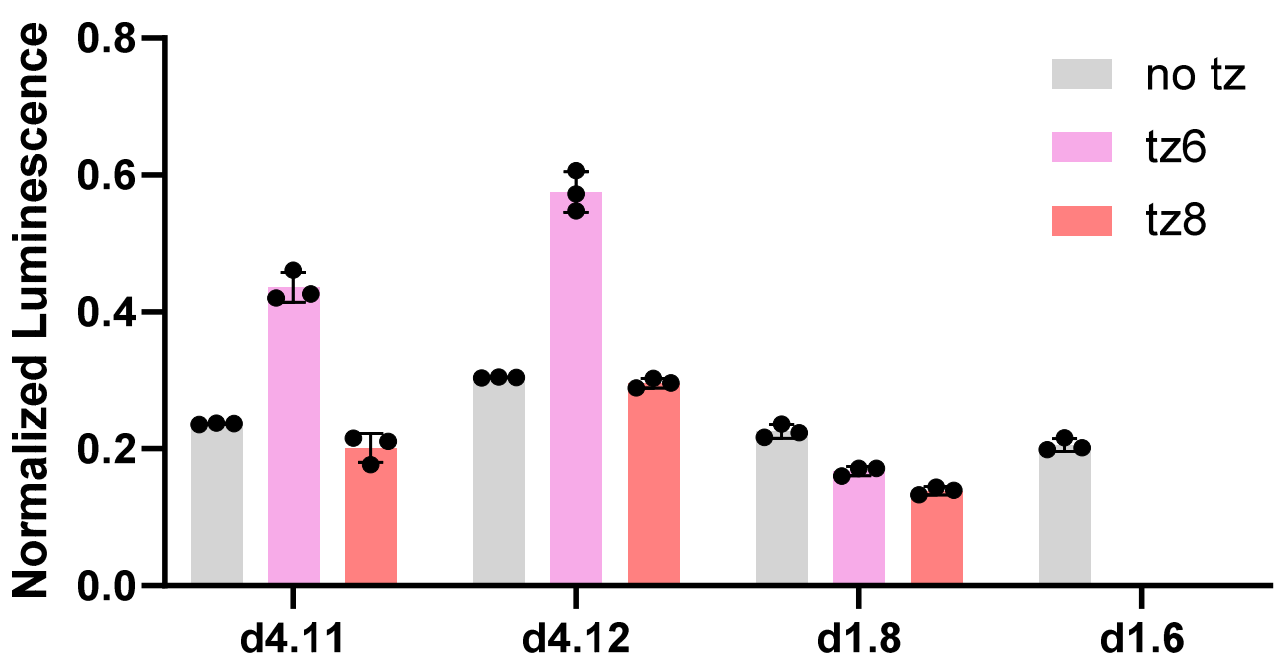

Figure S14 Normalized luminescence data derived from Figure S13. Luminescence data were normalized to samples treated with scrambled control siRNA **d0.2** and the corresponding tetrazine. Mean ± SD values (n = 3) are shown.

LC-MS data for p2-modified guide strands

G1-VdU2-p2 Calculated mass: 7269.3128

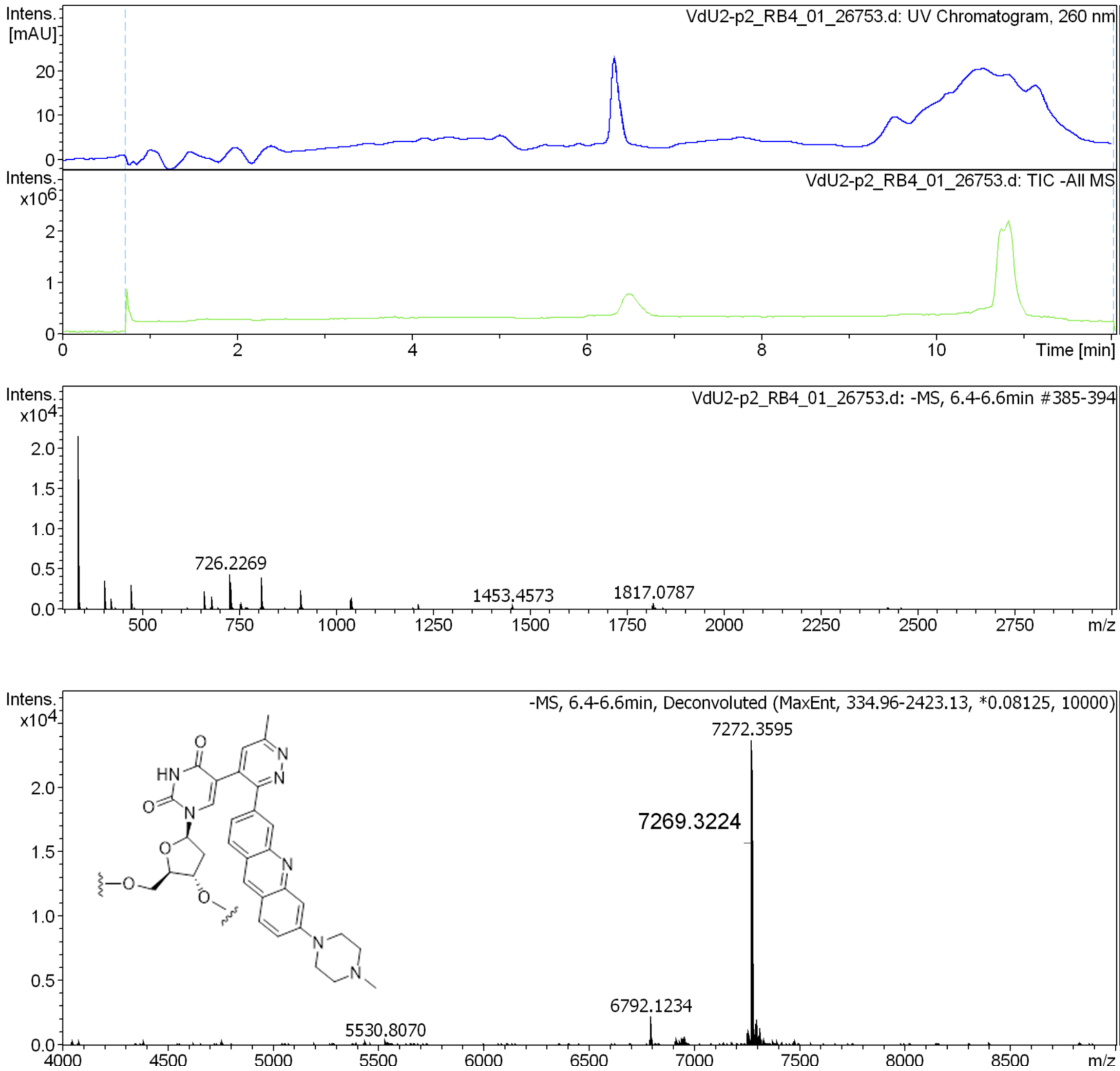

Figure S15 LC-MS data for **G1-VdU2-p2**.

G1-VdA3-p2 Calculated mass: 7258.3012

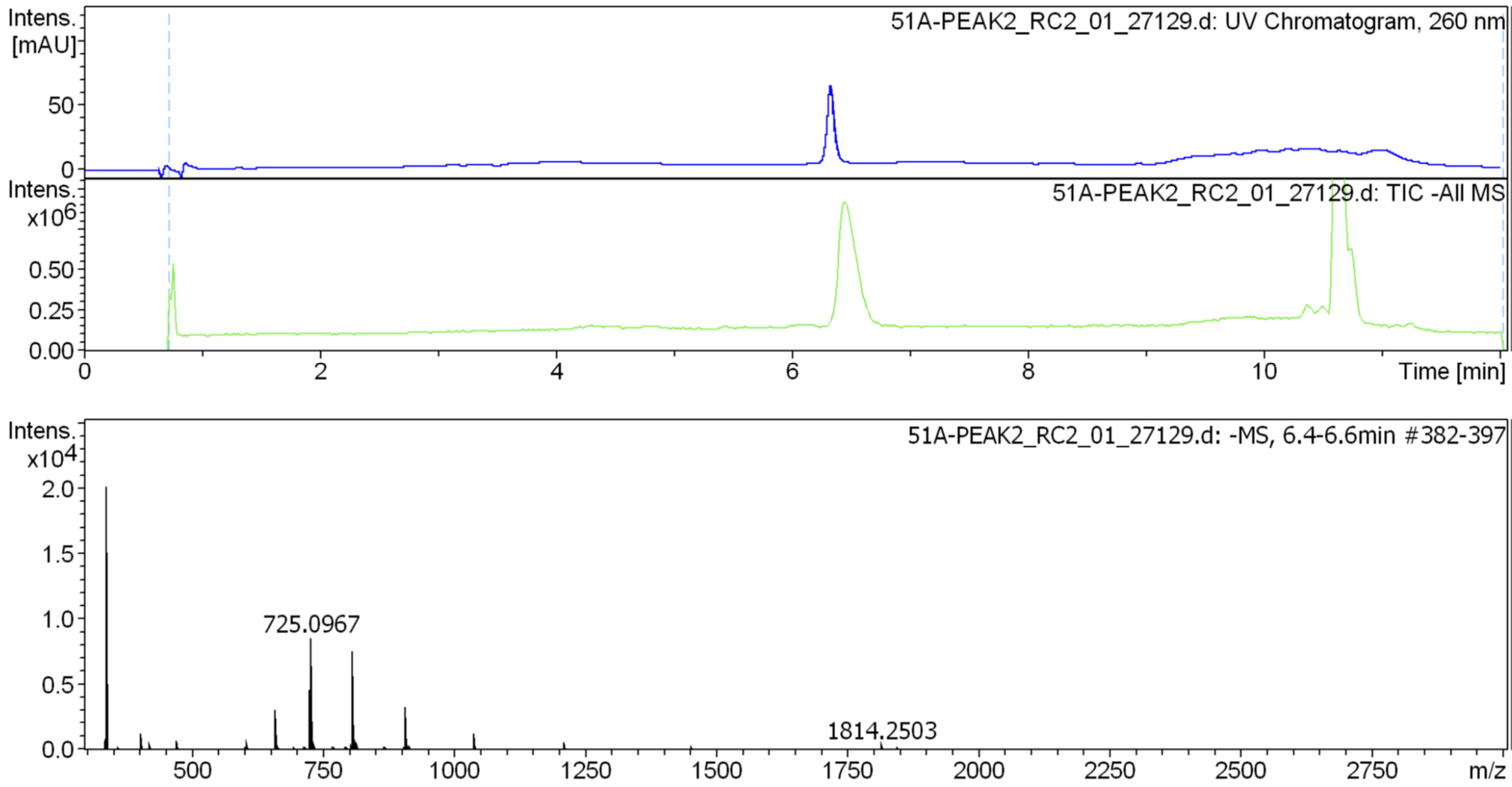

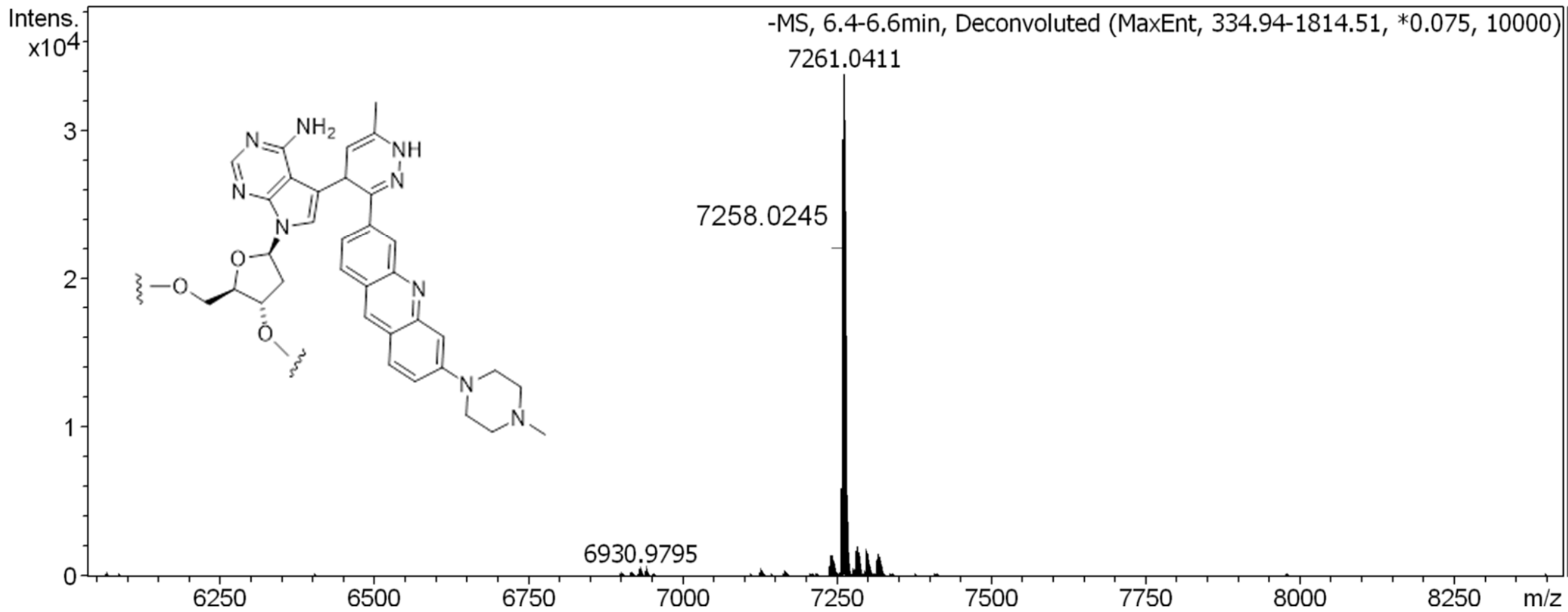

Figure S16 LC–MS data for **G1-VdA3-p2.**

G1-VdA4-p2 Calculated mass: 7258.3012

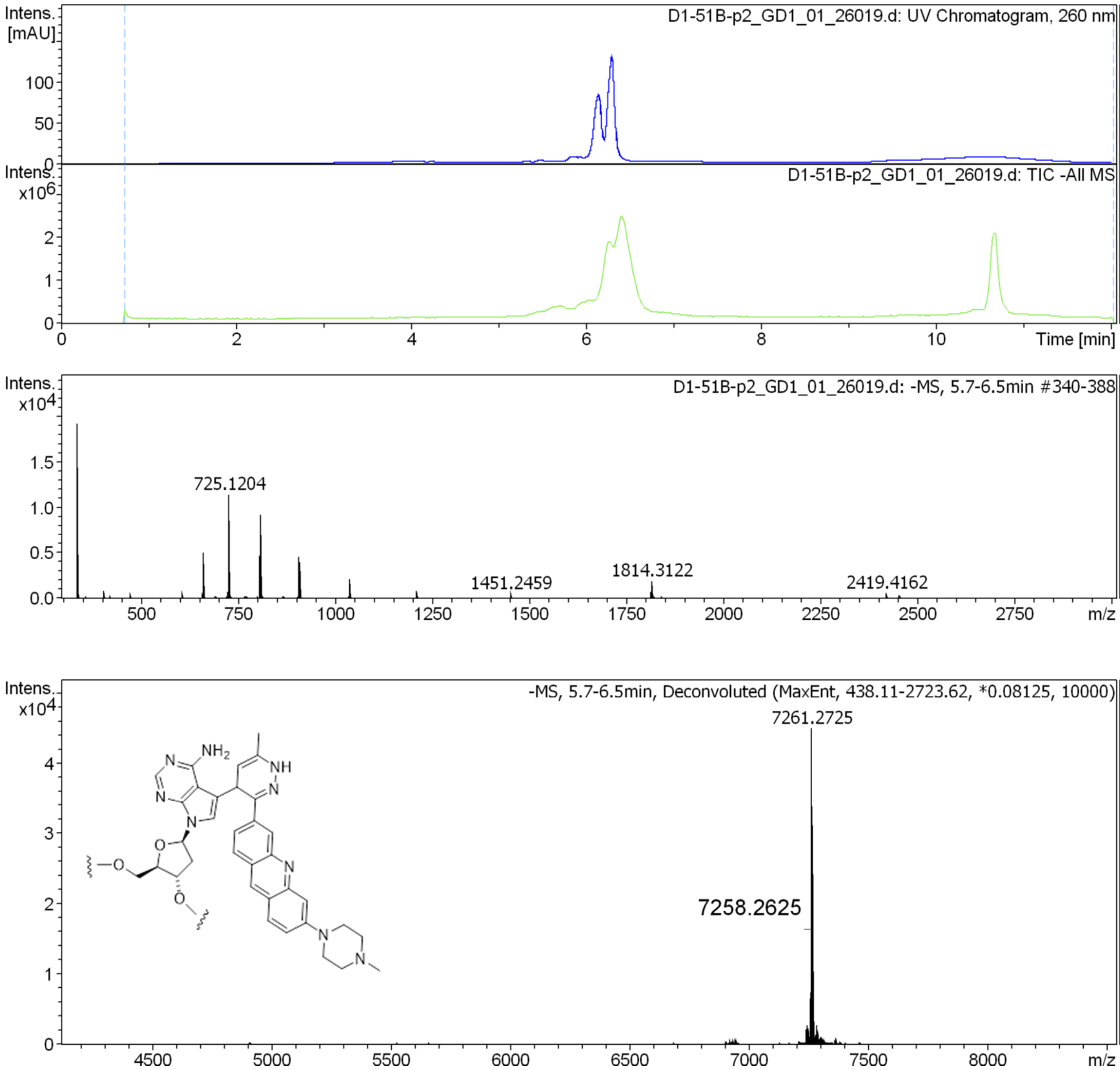

Figure S17 LC-MS data for **G1-VdA3-p2**. Two isomers with the same molecular weight were found in HPLC preparation but not separated. They were mixed and used in silencing assay.

G1-VdU5-p2 Calculated mass 7257.2976

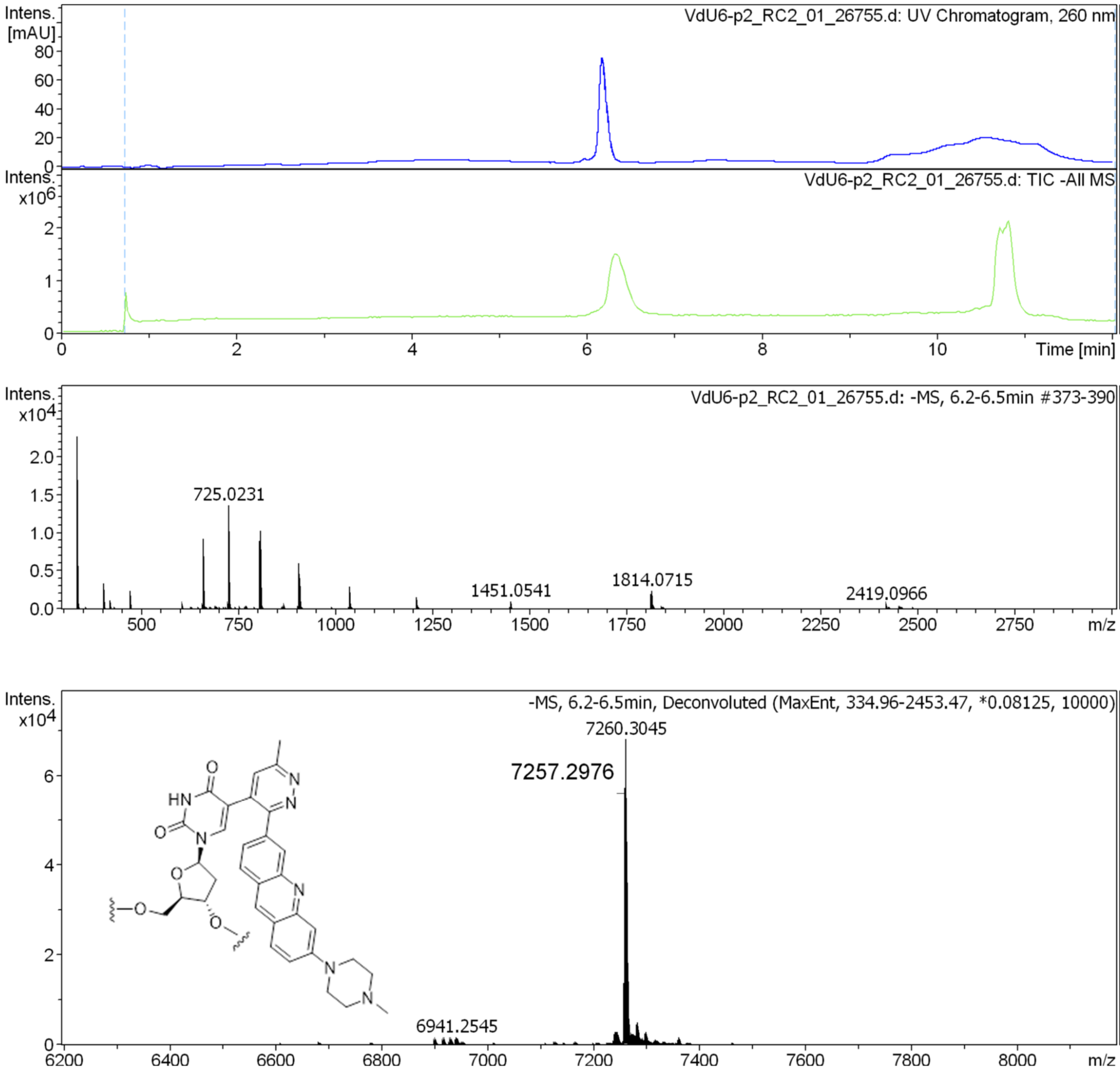

Figure S18 LC-MS data for **G1-VdU5-p2**.

G1-VdU6-p2 Calculated mass: 7269.3128

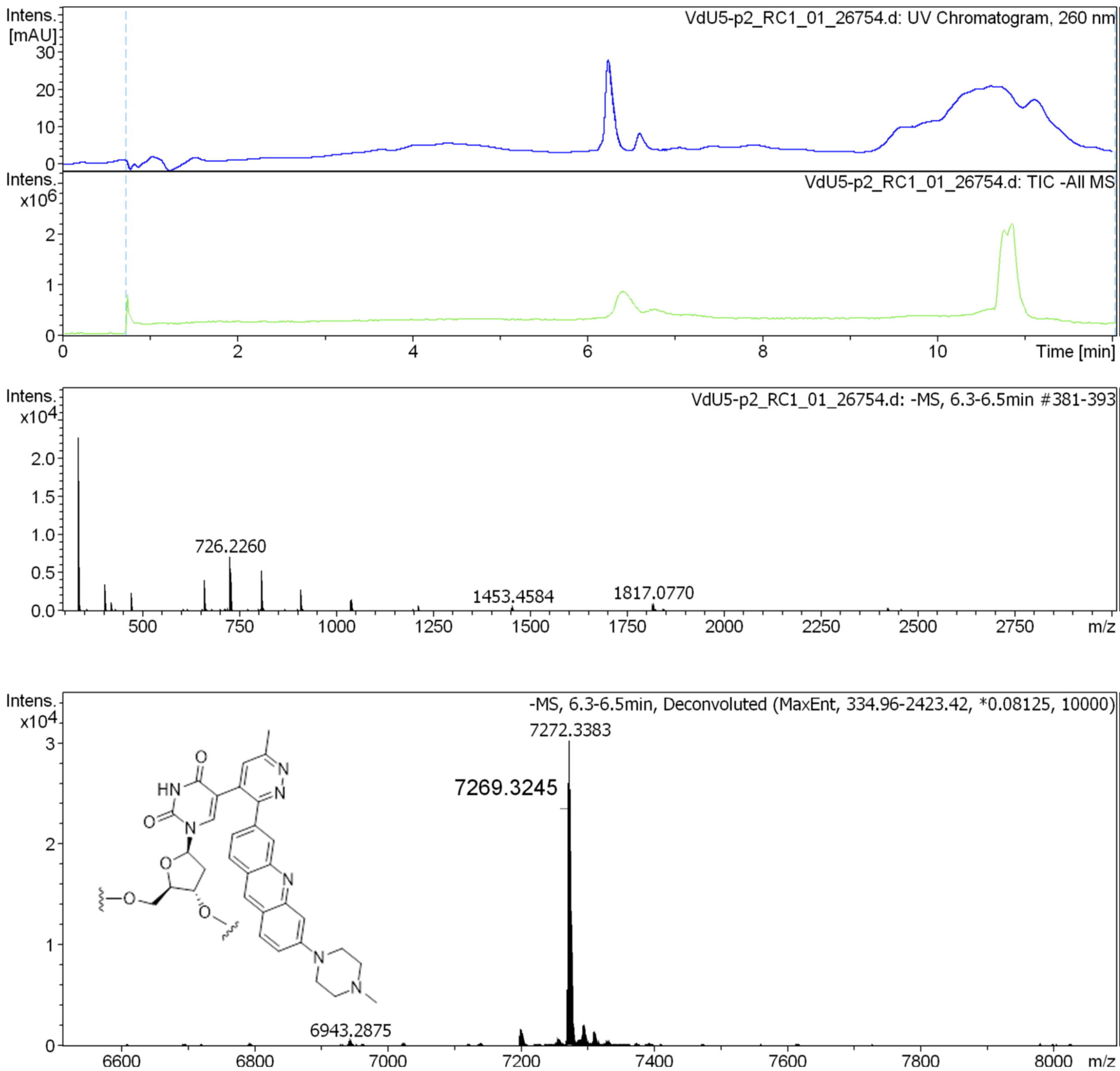

Figure S19 LC-MS data for **G1-VdU6-p2**.

G1-VdA7-p2 Calculated mass: 7258.3012

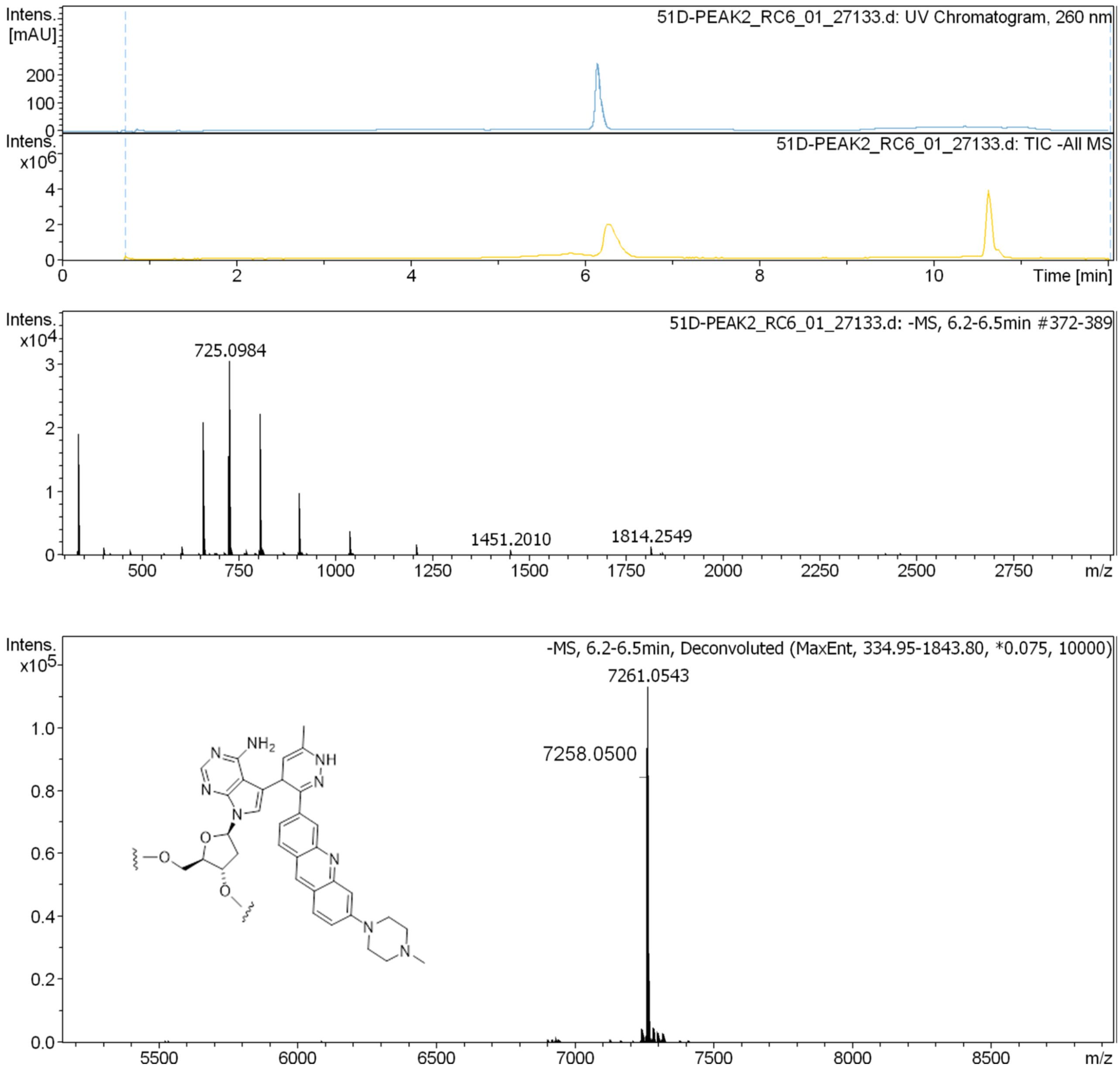

Figure S20 LC-MS data for **G1-VdA3-p2**.

G1-VdU14-p2 Calculated mass: 7269.3128

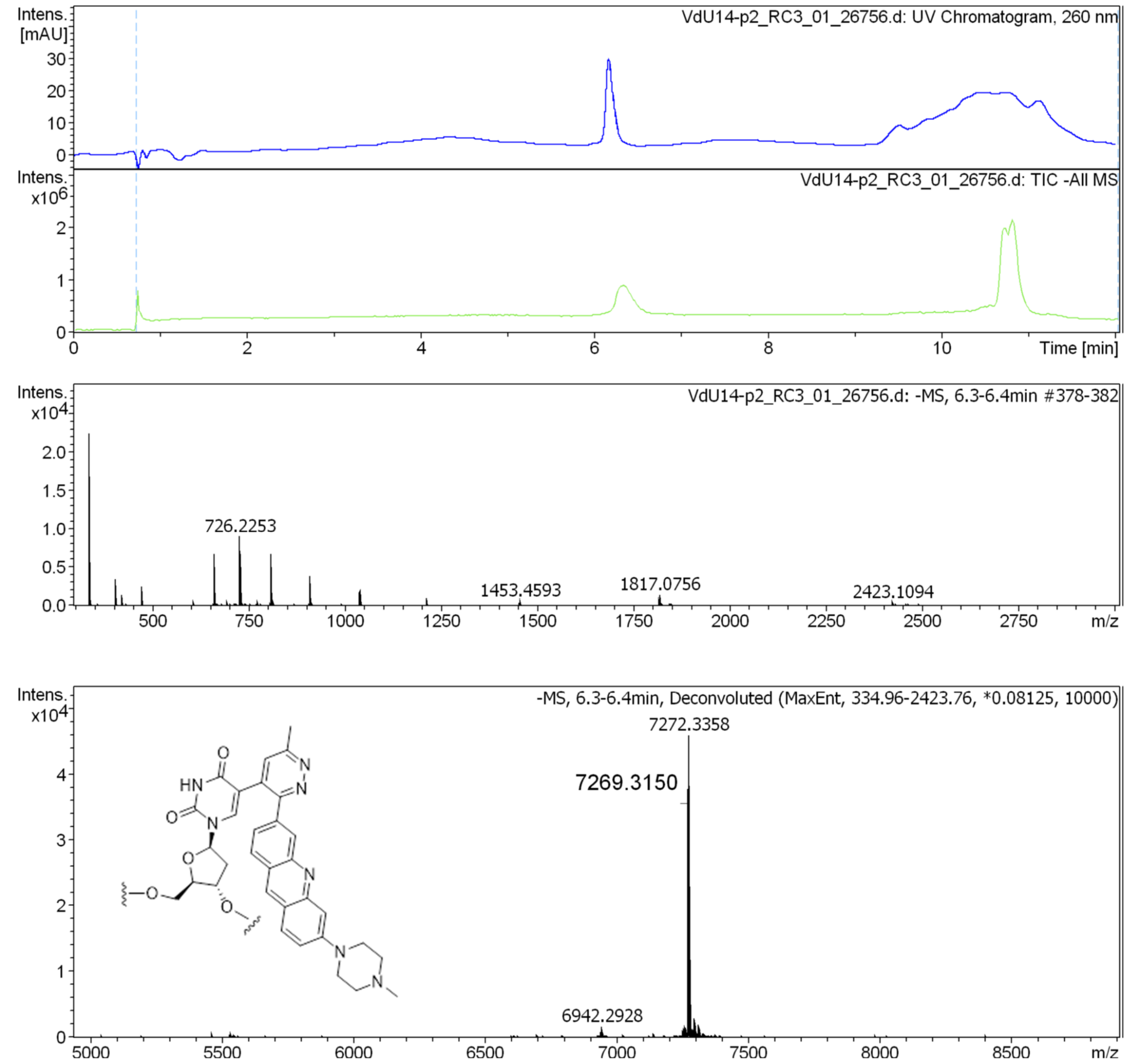

Figure S21 LC-MS data for **G1-VdU14-p2**.

Synthesis procedures

6-(4-methylpiperazin-1-yl)acridine-3-carbonitrile (**S1**)

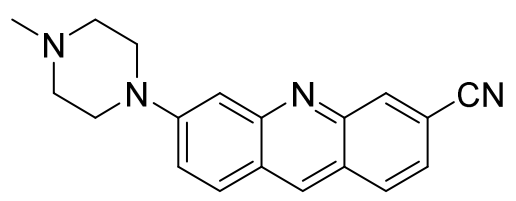

The synthesis procedure was adapted from a literature procedure with slight modifications.[2] To a 100 mL round bottom flask were added 3-(4-methylpiperazin-1-yl)aniline (1.00 g, 5.23 mmol), dppf (292 mg, 526 µmol), and K_2CO_3 (1.517 g, 11.0 mmol). The flask was evacuated and refilled with Ar three times, followed by the addition of 28 mL dry, degassed toluene. $Pd_2(dba)_3$ (119 mg, 130 µmol) and 3-bromo-4-formylbenzonitrile (1.072 g, 5.79 mmol) were then added to the flask and stirred under Ar for 2 d at 80°C. The resulting mixture was filtered through celite then mixed with silica gel for 2 h before being loaded into a silica gel column. The crude was eluted from the silica using 5% MeOH in DCM, dried, followed by trituration with ether to afford **S1** as an orange solid (662 mg, 2.19 mmol, 42%).

^{1}H NMR (500 MHz, $CDCl_3$) δ 8.57 (s, 1H), 8.45 (p, *J* = 0.8 Hz, 1H), 7.97 (d, *J* = 8.5 Hz, 1H), 7.84 (d, *J* = 9.4 Hz, 1H), 7.49 (dd, *J* = 8.6, 1.5 Hz, 1H), 7.45 (dd, *J* = 9.4, 2.4 Hz, 1H), 7.32 (d, *J* = 2.4 Hz, 1H), 3.53 – 3.48 (m, 4H), 2.66 – 2.60 (m, 4H), 2.38 (s, 3H).

^{13}C NMR (126 MHz, $CDCl_3$) δ 153.1, 152.3, 147.8, 135.4, 135.3, 129.9, 129.0, 126.4, 123.7, 123.5, 121.7, 119.1, 113.3, 107.7, 54.9, 48.2, 46.3.

HRMS(ESI) Calculated m/z for $C_{19}H_{19}N_4^+$ $[M+Na]^+$: 303.16042 found: 303.15998

(3-(6-methyl-1,2,4,5-tetrazin-3-yl)-6-(4-methylpiperazin-1-yl)acridin-10-ium chloride) (**p2-HCl**)

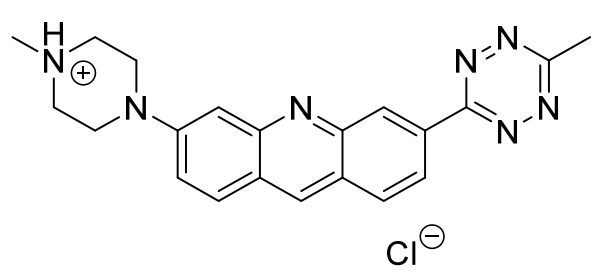

The synthesis procedure was adapted from a literature procedure with slight modifications.[2] To a 150 mL pressure tube were added **S1** (331 mg, 1.09 mmol) and $Zn(OTf)_2$ (201 mg, 554 µmol). The tube was evacuated and refilled with Ar three times, followed by addition of MeCN (590 µL, 11.3 mmol) and N_2H_4 (1 M in THF, 55 mL, 55 mmol). The tube was sealed and heated to 65°C for 3 d. The mixture was then evaporated to dryness, redissolved in 2 mL DCM, followed by addition of $PhI(OAc)_2$ (578 mg, 1.80 mmol). The mixture was stirred at RT overnight, then directly loaded onto a silica column. The crude was purified by silica column chromatography (eluted with 0-0.5% MeOH, 0.5% Et_3N in DCM). The resulting **p2-FB** was dissolved in DMSO followed by addition of 1 mL of 4 M HCl in dioxane, then precipitated with Et_2O to afford **p2-HCl** as a red solid (134 mg, 329 µmol, 30%).

^{1}H NMR (500 MHz, D_2O) δ 8.98 (s, 1H), 8.32 (dd, *J* = 8.6, 1.6 Hz, 1H), 8.26 – 8.24 (m, 1H), 8.21 (d, *J* = 8.6 Hz, 1H), 7.80 (d, *J* = 9.6 Hz, 1H), 7.34 (dd, *J* = 9.6, 2.4 Hz, 1H), 6.73 (d, *J* = 2.4 Hz, 1H), 4.50 – 4.42 (m, 2H), 3.88 – 3.79 (m, 2H), 3.70 – 3.60 (m, 2H), 3.45 – 3.35 (m, 2H), 3.18 (s, 3H), 3.08 (s, 3H).

^{13}C NMR (126 MHz, D_2O) δ 168.0, 162.2, 155.2, 144.8, 142.8, 137.6, 136.5, 132.1, 131.3, 124.3, 122.8, 122.7, 119.3, 116.3, 94.5, 52.6, 44.0, 43.0, 20.4.

HRMS(ESI) Calculated m/z for $C_{21}H_{22}N_7^+$ $[M+H]^+$: 372.19312 found: 372.19199

7-vinyl-7-deaza-2'-deoxyadenosine (**VdA**)

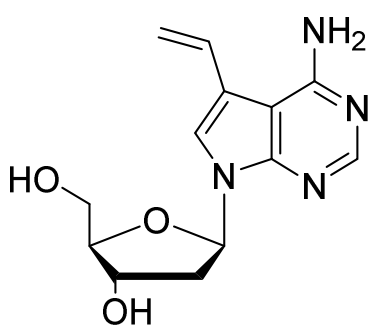

The synthesis was adapted from a literature procedure.[5] To a 100 mL round bottom flask were added 7-iodo-7-deaza-2'-deoxyadenosine (500 mg, 1.33 mmol), triphenylphosphine-3,3',3''-trisulfonic acid trisodium salt (TPPTS, 214 mg, 271 µmol), potassium vinyltrifluoroborate (214 mg, 1.60 mmol), and Cs_2CO_3 (2.176 g, 6.68 mmol), followed by $Pd(OAc)_2$ (30.4 mg, 135 µmol). The flask was evacuated and refilled with Ar three times, then 25 mL of degassed MeCN/H_2O mixture (1:2, v/v) was added and heated to 80°C for 3 h, then cooled to room temperature (RT) and filtered through celite. The crude material was adsorbed onto silica and purified by silica column chromatography (eluted with 0.5%–7% MeOH in DCM) to afford **VdA** as an off white solid (279 mg, 1.01 mmol, 76%)

^{1}H NMR (500 MHz, DMSO-d_6) δ 8.05 (s, 1H), 7.65 (s, 1H), 7.10 (dd, *J* = 17.3, 10.9 Hz, 1H), 6.72 (s, 2H), 6.51 (dd, *J* = 8.3, 5.9 Hz, 1H), 5.57 (dd, *J* = 17.2, 1.7 Hz, 1H), 5.25 (d, *J* = 4.1 Hz, 1H), 5.12 (dd, *J* = 10.9, 1.8 Hz, 1H), 5.08 (t, *J* = 5.6 Hz, 1H), 4.35 (dq, *J* = 5.9, 2.8 Hz, 1H), 3.82 (td, *J* = 4.5, 2.5 Hz, 1H), 3.58 (dt, *J* = 11.7, 4.6 Hz, 1H), 3.51 (dt, *J* = 11.6, 4.9 Hz, 1H), 2.16 (ddd, *J* = 13.0, 5.9, 2.6 Hz, 1H).(one of the two 2'-H have signal overlapping with solvent residual peak by COSY and HSQC spectrum).

^{13}C NMR (126 MHz, DMSO-d_6) δ 157.6, 151.5, 150.4, 129.1, 118.6, 114.2, 113.0, 100.6, 87.3, 82.9, 71.0, 62.0, 39.6.

HRMS calculated for $C_{13}H_{17}N_4O_3^+$ $[M+H]^+$ 277.12952, found 277.12938.

N^6-benoyl-7-vinyl-7-deaza-2'-deoxyadenosine (**Bz-VdA**)

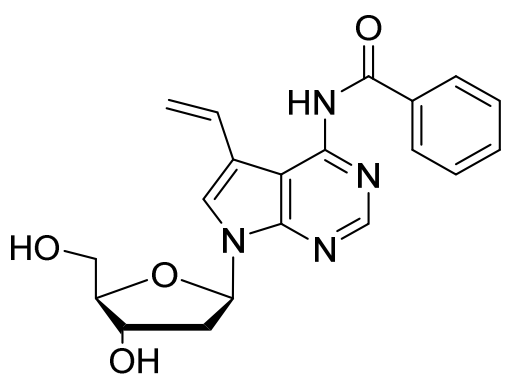

To a 25 mL round bottom flask was added **VdA** (400 mg, 1.45 mmol). The flask was evacuated and refilled with Ar, followed by addition of dry pyridine (4.0 mL). The mixture was cooled to 0°C and was slowly added benzoyl chloride (0.84 mL, 7.24 mmol). The mixture slowly warmed up to RT and was stirred for 6 h. Upon full conversion to fully benzoylated intermediate by TLC, the mixture was cooled to 0°C and was added MeOH to quench the residual benzoyl chloride. The mixture was diluted with DCM, then washed with 1×20 mL saturated $NaHCO_3$, 1×20 mL saturated NH_4Cl, and 1×20 mL brine. The organic layer was dried over $MgSO_4$, evaporated and redissolved in mixture of MeOH (12.4 mL) and pyridine (3.2 mL). To the solution was slowly added NaOH (2 M in H_2O, 6.4 mL) and stirred at RT for 15 min. The reaction was quenched with AcOH to neutral pH and adsorbed onto silica. The crude material was purified with silica column chromatography (eluted with 0.1–4% MeOH in DCM) to afford **Bz-VdA** as a light-yellow solid (447.3 mg, 1.18 mmol, 81%). A small analytical sample was obtained by cleaning up with C18 SPE cartridge.

^{1}H NMR (500 MHz, DMSO-d_6) δ 11.07 (s, 1H), 8.69 (s, 1H), 8.07 (s, 1H), 8.06 – 7.99 (m, 2H), 7.64 (t, *J* = 7.4 Hz, 1H), 7.56 (t, *J* = 7.6 Hz, 2H), 6.71 (dd, *J* = 8.0, 6.0 Hz, 1H), 6.61 (dd, *J* = 17.7, 11.1 Hz, 1H), 5.58 (dd, *J* = 17.7, 1.6 Hz, 1H), 5.34 (d, *J* = 4.0 Hz, 1H), 5.00 (dd, *J* = 9.4, 2.7 Hz, 2H), 4.41 (dd, *J* = 6.5, 3.4 Hz, 1H), 3.88 (td, *J* = 4.6, 2.6 Hz, 1H), 3.62 (dt, *J* = 9.3, 4.3 Hz, 1H), 3.55 (dt, *J* = 11.7, 4.2 Hz, 1H), 2.61 (ddd, *J* = 13.6, 8.1, 5.8 Hz, 1H), 2.29 (ddd, *J* = 13.2, 6.1, 2.9 Hz, 1H).

^{13}C NMR (126 MHz, DMSO-d_6) δ 166.7, 152.4, 151.3, 150.6, 133.3, 132.4, 128.6, 128.2, 128.1, 123.5, 114.5, 113.3, 111.5, 87.5, 82.8, 70.9, 61.8, 39.6.

HRMS(ESI) Calculated m/z for $C_{20}H_{20}N_4O_4Na^+$ $[M+Na]^+$: 403.1377 found: 403.1371

5'-*O*-dimethoxytrityl-*N*^{6}-benoyl-7-vinyl-7-deaza-2'-deoxyadenosine (**DMTr-VdA**)

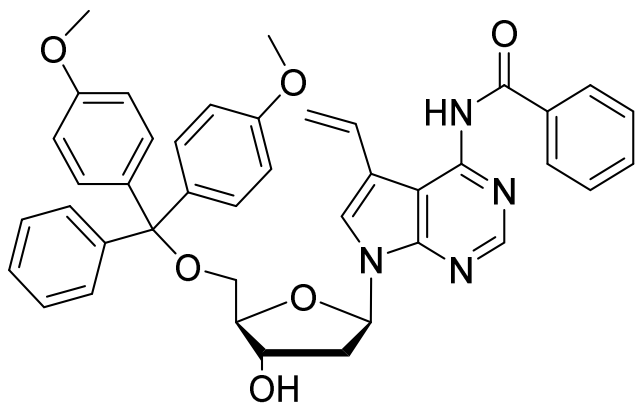

To a 100 mL round bottom flask was added **Bz-VdA** (750 mg, 1.97 mmol). The flask was evacuated and refilled with Ar three times, followed by addition of dry pyridine (20 mL). DMTr-Cl (1.34 g, 3.94 mmol) was slowly added to the mixture under stirring, then the mixture was stirred under Ar at RT overnight. The reaction mixture was diluted with DCM and washed with 3×50 mL saturated $NaHCO_3$, 1×50 mL brine. The organic layer was dried over $MgSO_4$, concentrated and purified with silica column chromatography (eluted with 0.05–2% MeOH, 1% Et_3N in DCM) to afford **DMTr-VdA** as an off-white solid (550 mg, 805 µmol, 41%).

^{1}H NMR (500 MHz, $CDCl_3$) δ 8.33 (s, 1H), 8.13 (d, *J* = 7.6 Hz, 2H), 7.58 – 7.53 (m, 1H), 7.52 – 7.46 (m, 3H), 7.45 – 7.42 (m, 2H), 7.32 (dq, *J* = 8.5, 3.2 Hz, 4H), 7.31 – 7.25 (m, 2H), 7.24 – 7.20 (m, 1H), 7.08 (s, 1H), 6.84 – 6.80 (m, 4H), 6.78 (t, *J* = 6.7 Hz, 1H), 5.33 (dd, *J* = 17.8, 1.4 Hz, 1H), 5.12 (dd, *J* = 11.0, 1.4 Hz, 1H), 4.67 (dt, *J* = 6.5, 3.5 Hz, 1H), 4.12 (q, *J* = 3.9 Hz, 1H), 3.78 (d, *J* = 1.0 Hz, 6H), 3.45 – 3.37 (m, 2H), 2.67 (ddd, *J* = 13.5, 7.2, 6.1 Hz, 1H), 2.49 (ddd, *J* = 13.5, 6.3, 3.7 Hz, 1H).

^{13}C NMR (126 MHz, $CDCl_3$) δ 158.7, 150.5 (from ^{1}H-^{13}C-HMBC signal), 144.7, 135.8, 135.7, 132.3, 130.2, 130.2, 128.7, 128.6, 128.3, 128.1, 127.1, 116.8 (from ^{1}H-^{13}C-HMBC signal), 114.9, 113.4, 108.3, 86.8, 85.9, 83.6, 72.8, 64.1, 55.4, 41.1.

HRMS(ESI) calculated for $C_{41}H_{38}N_4O_6Na^+$ $[M+Na]^+$: 705.2684, found 705.2677.

5'-*O*-dimethoxytrityl-N^6-benoyl-7-vinyl-7-deaza-2'-deoxyadenosine, 3'- (2-cyanoethyl) diisopropylphosphoramidite (**VdA-amidite**)

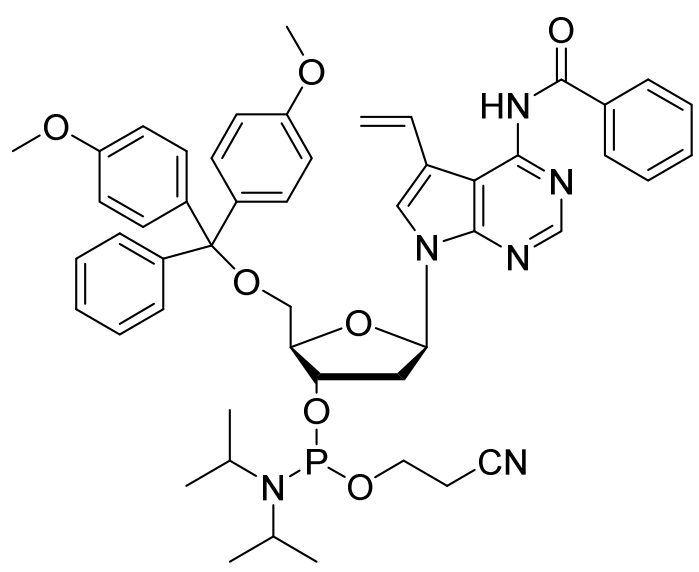

To a 10 mL round bottom flask was added **DMTr-VdA** (250 mg, 266 µmol). The flask was evacuated and refilled with Ar three times, followed by addition of dry DCM (1.5 mL) and dry DIPEA (0.16 mL, 915 µmol). The mixture was cooled to 0°C, followed by dropwise addition of 2-Cyanoethyl *N*,*N*-diisopropylchlorophosphoramidite (0.17 mL, 732 µmol). The mixture was let slowly warmed up to RT and stirred for 1 h. The mixture was directly purified by silica column chromatography to afford **VdA-amidite** as a white solid (234.1 mg, 265 µmol, 72%).

^{1}H NMR (500 MHz, $CDCl_3$) mixture of diastereomers, see spectrum.

^{31}P NMR (203 MHz, $CDCl_3$) δ 148.8, 148.6.

HRMS(ESI) calculated for $C_{50}H_{56}N_6O_7P^+$ $[M+H]^+$: 883.39426, found 883.39528.

3-methyl-6-(pyridin-2-yl)-1,2,4,5-tetrazine (**tz1**)

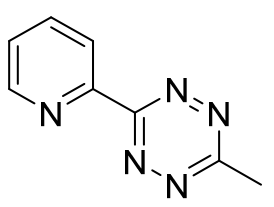

The synthesis was adapted from a literature procedure.[6] To a 20 mL pressure tube was added 2-pyridinecarbonitrile (100 µL, 1.04 mmol) and $Zn(OTf)_2$ (185 mg, 510 µmol). The tube was evacuated and refilled with Ar 3 times, followed by addition of MeCN (580 µL, 11.1 mmol) and hydrazine monohydrate (2.5 mL, 51.5 mmol). The tube was sealed and the mixture was heated to 65°C for 24 h. Ater reaction, the mixture was transferred to an Erlenmeyer flask with water and DCM, followed by addition of $NaNO_2$ (~2 g). The mixture was vigorously stirred during which HCl (6 M) was slowly added until no bubbles forming and the pH of the solution is < 3 (Caution! Toxic nitrogen oxide gases produced in this step, perform this step in a well-ventilated fumehood). The mixture was neutralized with $KHCO_3$ until pH is ~8, then was extracted with 3×10 mL DCM. The organic layer was washed with 1×10 mL water, 1×10 mL brine, dried over $MgSO_4$ and concentrated. The crude material was purified by silica column chromatography (eluted with 0-10% EtOAc in toluene) to afford **tz1** as a pink solid (28.1 mg, 162 µmol, 16%). The analytical data is in accordance with published data.[6]

^{1}H NMR (500 MHz, $CDCl_3$) δ 8.94 (ddd, J = 4.8, 1.8, 0.9 Hz, 1H), 8.64 (dt, J = 7.9, 1.1 Hz, 1H), 7.98 (td, J = 7.8, 1.8 Hz, 1H), 7.55 (ddd, J = 7.7, 4.8, 1.2 Hz, 1H), 3.16 (s, 3H).

^{13}C NMR (126 MHz, $CDCl_3$) δ 168.3, 163.8, 151.0, 150.4, 137.6, 126.5, 124.0, 21.5.

tert-butyl ((1,2,4,5-tetrazin-3-yl)methyl)carbamate (**Boc-tz3**)

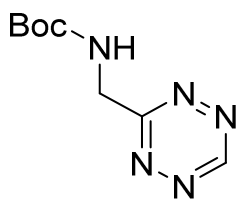

The synthesis was adapted from a literature procedure.[7] To a 10 mL round bottom flask was added *tert*-butyl (cyanomethyl)carbamate (251 mg, 1.61 mmol), $Zn(OTf)_2$ (291 mg, 800 µmol), and formamidine acetate (1.691 g, 10.3 mmol). The flask was evacuated and refilled with Ar 3 times, followed by addition of hydrazine monohydrate (3.9 mL, 80 mmol). The mixture was heated to 30°C for 24 h, during which some white precipitate started to form. The reaction mixture was transferred to an Erlenmeyer flask with water and DCM, followed by addition of $NaNO_2$ solution (1.08 g in 10 mL water). The mixture was vigorously stirred during which HCl (6 M) was slowly added until no bubbles forming and the pH of the solution is < 3 (Caution! Toxic nitrogen oxide gases produced in this step, perform this step in a well-ventilated fumehood). The mixture was neutralized with $KHCO_3$ until pH is ~8, then was extracted with 3×30 mL DCM. The organic layer was washed with 1×30 mL brine, dried over $MgSO_4$ and concentrated. The crude material was purified with silica column chromatography (eluted with 0–40% EtOAc in hexanes) to afford **Boc-tz3** as a pink solid (133 mg, 629 µmol, 39%). The analytical data is in accordance with published data.[7]

^{1}H NMR (500 MHz, $CDCl_3$) δ 10.27 (s, 1H), 5.57 (s, 1H), 5.01 (d, *J* = 6.0 Hz, 2H), 1.46 (s, 9H).

^{13}C NMR (126 MHz, $CDCl_3$) δ 169.5, 158.8, 155.9, 80.7, 43.9, 28.4.

(1,2,4,5-tetrazin-3-yl)methanaminium chloride (**tz3-HCl**)

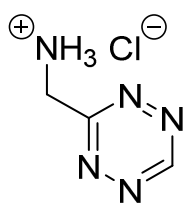

The synthesis was adapted from a literature procedure.[8] To a dry round bottom flask was added **Boc-tz3** (102 mg, 483 µmol), followed by slow addition of HCl (4 M in dioxane, 1.2 mL, 4.8 mmol). The mixture was vigorously stirred for 30 min at RT, during which a red precipitate was slowly formed. The mixture was filtered, washed with hexanes, and dissolved with 1:1 DCM/MeOH. The solution was evaporated to yield **tz3-HCl** as a red solid (62.0 mg, 420 µmol, 87%). Analytical data is in accordance with previously published data.[8]

^{1}H NMR (500 MHz, DMSO-d_6) δ 10.76 (s, 1H), 9.07 (s, 3H), 4.75 (s, 2H).

^{13}C NMR (126 MHz, DMSO-d_6) δ 166.3, 159.1, 41.2.

tert-butyl (4-cyanobenzyl)carbamate (**S2**)

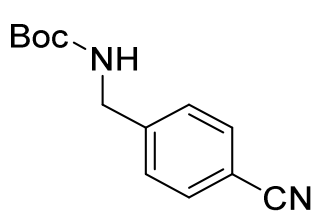

The synthesis was adapted from a literature procedure.[9] To an Erlenmeyer flask was added (4-cyanophenyl)methanaminium chloride (2.03 g, 17.0 mmol) and 20 mL water. The solution was neutralized with Na_2CO_3 until bubbling subsided, then was extracted with 4×20 mL DCM. The organic layer was washed with 20 mL brine and concentrated. HFIP (12 mL) was added to the flask, followed by addition of Boc_2O (2.80 g, 12.8 mmol). The mixture was stirred for 30 min, then diluted with 50 mL DCM, washed with 2×20 mL saturated

$NaHCO_3$ and 1×20 mL brine. The organic layer was dried over $MgSO_4$ and evaporated to afford **S2** as a white solid (2.47 g, 10.6 mmol, 88%). The analytical data is in accordance with published data.[10]

^{1}H NMR (500 MHz, $CDCl_3$) δ 7.65 – 7.59 (m, 2H), 7.41 – 7.36 (m, 2H), 4.96 (s, 1H), 4.37 (d, *J* = 6.2 Hz, 2H), 1.46 (s, 9H).

^{13}C NMR (126 MHz, $CDCl_3$) δ 156.0, 144.8, 132.6, 127.9, 118.9, 111.3, 80.2, 44.4, 28.5.

tert-butyl (4-(6-(pyridin-2-yl)-1,2,4,5-tetrazin-3-yl)benzyl)carbamate (**Boc-tz5**)

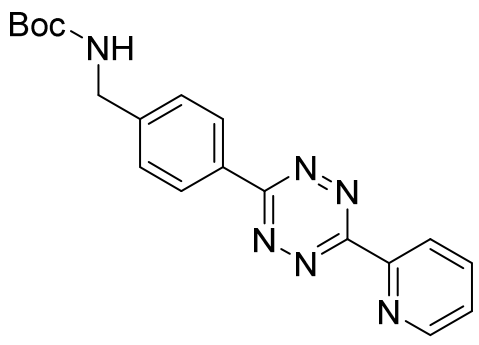

To a 48 mL pressure tube was added *tert*-butyl (4-cyanobenzyl)carbamate (503 mg, 2.17 mmol), 2-pyridinecarbonitrile (2.239 g, 21.5 mmol) and $Zn(OTf)_2$ (407 mg, 1.12 mmol). The tube was evacuated and refilled with Ar 3 times, followed by addition of hydrazine monohydrate (5.4 mL). The mixture was heated at 60°C for 20 h, transferred to an Erlenmeyer flask with water and DCM, and acidified to pH 2. Then to the mixture $NaNO_2$ was slowly added until no bubbles forming (Caution! Toxic nitrogen oxide gases produced in this step, perform this step in a well-ventilated fumehood). The mixture was extracted with 3×20 mL DCM. The combined organic layer was washed with 20 mL brine, dried over $MgSO_4$, and concentrated. The crude material was purified by silica column chromatography (0–15% EtOAc in toluene) to afford **Boc-tz5** as a dark purple solid (281 mg, 772 μmol, 36%). The analytical data is in accordance with published data.[10]

^{1}H NMR (500 MHz, $CDCl_3$) δ 8.96 (dt, *J* = 4.7, 1.5 Hz, 1H), 8.68 (dd, *J* = 7.9, 1.1 Hz, 1H), 8.67 – 8.63 (m, 2H), 7.99 (td, *J* = 7.8, 1.8 Hz, 1H), 7.56 (ddd, *J* = 7.6, 4.7, 1.2 Hz, 1H), 7.53 (d, *J* = 8.2 Hz, 2H), 5.04 (s, 1H), 4.45 (d, *J* = 6.2 Hz, 2H), 1.48 (s, 9H).

^{13}C NMR (126 MHz, $CDCl_3$) δ 164.3, 163.5, 156.1, 151.1, 150.5, 144.7, 137.6, 130.7, 128.8, 128.3, 126.4, 124.0, 80.0, 44.5, 28.5.

HRMS(ESI) calculated m/z for $C_{19}H_{21}N_6O_2^+$ $[M+H]^+$: 365.1721 found: 365.1719

tz5-HCl

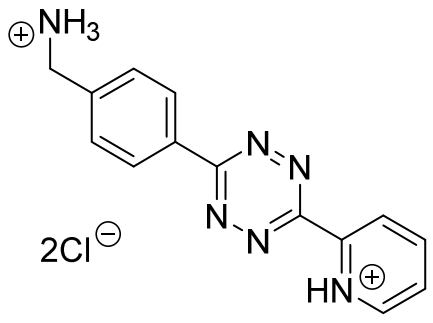

The synthesis was adapted from a literature procedure. To a 25 mL round bottom flask was added **Boc-tz5** (466 mg, 1.28 mmol). The flask was evacuated and refilled with Ar 3 times, followed by addition of HCl (4 M in dioxane, 3.2 mL, 12.8 mmol) under vigorous stirring. The mixture was stirred at RT for 2.5 h, then filtered. The precipitate was washed with acetone and DCM to afford **tz5-HCl** as a pink solid (405 mg, 1.20 mmol, 94%). The analytical data is in accordance with published data.[10]

^{1}H NMR (500 MHz, DMSO-d_6) δ 8.94 (ddd, *J* = 4.7, 1.7, 0.9 Hz, 1H), 8.78 (s, 3H), 8.61 (dt, *J* = 7.9, 1.1 Hz, 1H), 8.60 – 8.56 (m, 2H), 8.20 (td, *J* = 7.7, 1.7 Hz, 1H), 7.87 – 7.82 (m, 2H), 7.77 (ddd, *J* = 7.6, 4.8, 1.2 Hz, 1H), 7.60 (s, 1H), 4.19 (d, *J* = 5.8 Hz, 1H), 4.17 (d, *J* = 6.1 Hz, 1H).

^{13}C NMR (126 MHz, DMSO-d_6) δ 163.3, 163.0, 150.2, 149.7, 139.1, 138.5, 131.6, 130.0, 128.0, 126.8, 124.2, 41.8.

HRMS(ESI) Calculated m/z for $C_{14}H_{13}N_6^+$ $[M+H]^+$: 265.11962 found: 265.11900

2-Dimentylaminomethylene-1,3-bis(dimethylimmonio)propane bis(tetrafluoroborate) (**S3**)

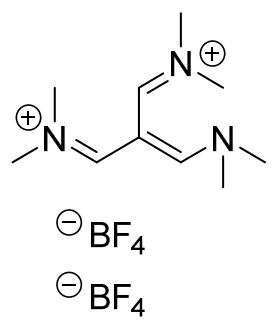

The synthesis was adapted from a literature procedure.[11] To a 250 mL round bottom flask was added DMF (18.9 mL, 244 mmol). The flask was cooled to 0°C, then $POCl_3$ (11.2 mL, 120 mmol) was added dropwise. Bromoacetic acid (5.56 g, 40.01 mmol) was then added to the flask, and the mixture was heated to 110°C for 3 h. The resulting mixture was cooled to 0°C and was added HBF_4 (12.8 mL 48% solution in H_2O mixed with 23 mL MeOH) dropwise over 30 min, followed by 23 mL isopropanol. The mixture was stirred at 0°C for 1 h, then filtered. The precipitate was washed with EtOAc, briefly air dried, then recrystallized from 60 mL MeCN to afford **S3** as light yellow crystalline solid (7.31 g, 20.5 mmol, 51%). The analytical data is in accordance with published data.[11]

^{1}H NMR (400 MHz, DMSO-d_6) δ 8.43 (s, 3H), 3.53 (s, 9H), 3.38 (s, 9H).

HRMS(ESI) Calculated m/z for $C_{10}H_{21}N_3^{2+}$ $[M]^{2+}$: 91.68623 found: 91.58640

7-methoxyquinoline-3-carbaldehyde (**S4**)

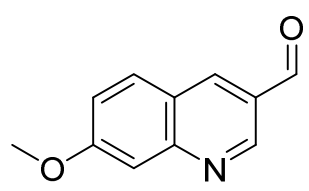

The synthesis was adapted from a literature procedure.[12] To a 20 mL pressure tube was added *m*-anisidine (200 µL, 1.78 mmol) and **S3** (1.27 g, 3.56 mmol), followed by ethanol (5 mL). The tube was sealed and heated to 85°C. The reaction was stirred overnight during which the mixture gradually turned to a dark orange solution. After reaction, the tube was cooled to RT and evaporated to dryness. The resulting solid was dissolved in 3 mL THF and 3 mL 1 M HCl, and was stirred at RT for 2 h, then quenched with saturated $NaHCO_3$. The mixture was extracted with 3×20 mL EtOAc. The combined organic layer was washed with 1×20 mL saturated $NaHCO_3$, 1×20 mL brine, dried over $MgSO_4$ and concentrated. The crude material was purified by silica column chromatography (eluted with 15–25% EtOAc in hexanes) to yield **S4** (202 mg, 1.08 mmol, 61%) as a white solid.

^{1}H NMR (500 MHz, $CDCl_3$) δ 10.19 (s, 1H), 9.27 (d, *J* = 2.1 Hz, 1H), 8.53 (dd, *J* = 2.1, 0.8 Hz, 1H), 7.85 (d, *J* = 9.0 Hz, 1H), 7.48 (d, *J* = 2.5 Hz, 1H), 7.29 (dd, *J* = 9.0, 2.5 Hz, 1H), 4.00 (s, 3H).

^{13}C NMR (126 MHz, $CDCl_3$) δ 190.7, 163.5, 153.0, 150.2, 139.2, 130.7, 127.2, 122.4, 121.4, 107.9, 55.9.

HRMS(ESI) Calculated m/z for $C_{11}H_{10}NO_2^+$ $[M+H]^+$: 188.0706 found: 188.0708

7-(4-methylpiperazin-1-yl)quinoline-3-carbaldehyde (**S5**)

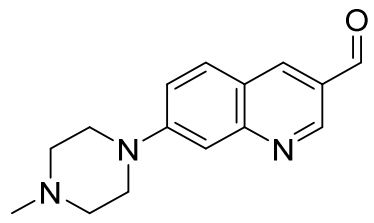

The synthesis was adapted from a literature procedure with slight modification.[12] To a 20 mL pressure tube was added 3-(4-methylpiperazin-1-yl)aniline (253 mg, 1.32 mmol) and **S3** (933 mg, 2.61 mmol), followed by ethanol (3 mL). The tube was sealed and heated to 85°C. The reaction was stirred overnight during which the mixture gradually turned to a dark orange solution. After reaction, the tube was cooled to RT and evaporated to dryness. The resulting solid was dissolved in 3 mL THF and 3 mL 1 M HCl, and was stirred at RT for 2 h, then quenched with saturated $NaHCO_3$ then Na_2CO_3. The mixture was extracted with 3×20 mL DCM. The combined aqueous layer was washed with 1×20 mL $NaHCO_3$, 1×20 mL brine, dried over $MgSO_4$ and concentrated. The crude was purified by silica column chromatography with 0.5% MeOH, 0.5% Et_3N in DCM to afford **S5** as a yellow solid (246 mg, 962 μmol, 73%).

^{1}H NMR (500 MHz, $CDCl_3$) δ 10.10 (s, 1H), 9.17 (d, *J* = 2.1 Hz, 1H), 8.40 (d, *J* = 2.1 Hz, 1H), 7.88 – 7.66 (m, 1H), 7.36 – 7.33 (m, 2H), 3.51 – 3.46 (m, 4H), 2.63 – 2.56 (m, 4H), 2.36 (s, 3H).

^{13}C NMR (126 MHz, $CDCl_3$) δ 190.6, 154.1, 152.9, 150.6, 138.8, 130.5, 126.3, 120.7, 119.1, 109.8, 54.8, 47.7, 46.2.

HRMS(ESI) Calculated m/z for $C_{15}H_{18}N_3O^+$ $[M+H]^+$: 256.1444 found: 256.1435

N,*N*,-2-trimethylquinolin-7-amine (**S6**)

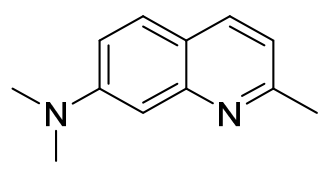

The synthesis was adapted from a literature procedure.[13] To a 50 mL round bottom flask was added N^1,N^1-dimethylbenzene-1,3-diaminium dichloride (1.022 g, 7.50 mmol) and 10.5 mL concentrated HCl (12 M)., followed by dropwise addition of paraldehyde (1.91 mL, 14.4 mmol). The mixture was stirred at RT overnight through which the color of mixture slowly turned dark. After all starting materials were consumed, the mixture was transferred to an Erlenmeyer flask and 10% NaOH solution was slowly added until solution turned milky. The mixture was extracted with 4×50 mL DCM, and the organic layer was washed with 3×50 mL saturated $NaHCO_3$ solution, and 1×50 mL brine. The organic phase was dried with $MgSO_4$, concentrated and purified with silica column chromatography (eluted with 10–25% EtOAc in hexanes) to yield **S6** as a dark orange to brown liquid (275 mg, 20%). The analytical data was in accordance with published data.[13]

^{1}H NMR (500 MHz, $CDCl_3$) δ 7.85 (d, *J* = 8.2 Hz, 1H), 7.62 – 7.55 (m, 1H), 7.10 (m, 2H), 6.99 (d, *J* = 8.2 Hz, 1H), 3.08 (s, 6H), 2.67 (s, 3H).

^{13}C NMR (126 MHz, $CDCl_3$) δ 159.2, 151.4, 149.8, 135.7, 128.2, 119.2, 118.1, 115.4, 106.8, 40.7, 25.5.

HRMS(APCI) Calculated m/z for $C_{12}H_{15}N_2^+$ $[M+H]^+$: 187.12298 found: 187.12178

7-(dimethylamino)quinoline-2-carbaldehyde (**S7**)

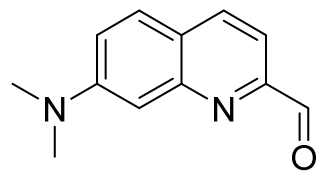

The synthesis was adapted from a literature procedure.[13] To a 25 mL round bottom flask was added **S6** (274 mg, 1.47 mmol), followed by 7.3 mL dioxane, then SeO_2 (268 mg, 2.41 mmol) and 470 µL H_2O. The mixture was heated to 80°C for 3 h, then filtered through celite, concentrated and purified with silica column chromatography (eluted with 5–10% EtOAc in hexanes) to afford **S7** (42.8 mg, 214 µmol, 15%) as a yellow solid. The analytical data was in accordance with published data.[13]

^{1}H NMR (500 MHz, $CDCl_3$) δ 10.17 (d, J = 0.9 Hz, 1H), 8.11 (d, J = 8.2 Hz, 1H), 7.76 – 7.69 (m, 2H), 7.30 (dd, J = 9.1, 2.6 Hz, 1H), 7.24 (d, J = 2.6 Hz, 1H), 3.15 (s, 7H).

^{13}C NMR (126 MHz, $CDCl_3$) δ 194.4, 153.0, 151.9, 150.1, 136.8, 128.5, 123.0, 119.0, 113.7, 107.0, 40.6.

HRMS(ESI) Calculated m/z for $C_{12}H_{12}N_2ONa^+$ $[M+Na]^+$: 223.0842 found: 223.0835

Diethyl ((6-methyl-1,2,4,5-tetrazin-3-yl)methyl)phosphonate (**S8**)

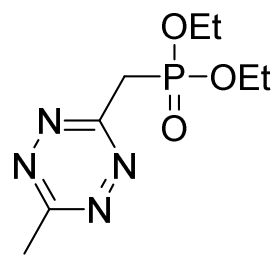

The synthesis was adapted from a literature procedure with slight modifications.[14] A solution of diethyl cyanomethyl phosphonate (2.43 mL, 15 mmol), acetonitrile (12.5 mL, 300 mmol), and 3-mercaptopropionic acid (1.32 mL, 15 mmol) in ethanol (5.9 mL) was cooled to 0°C. To this mixture was slowly added hydrazine hydrate (11.7 mL, 240 mmol). The reaction mixture was stirred at RT for 48 h and slowly turned light yellow. After the reaction was completed, sodium nitrile (15.5 g, 225 mmol) was added to the reaction, followed by slow addition of 6 M HCl until no bubbles forming and pH < 3 (Caution! Toxic nitrogen oxide gases produced in this step, perform this step in a well-ventilated fumehood). The pH was neutralized using $KHCO_3$. The resulting mixture was extracted with 4×50 mL DCM. The organic layer was washed with 3×50 mL water and 1×50 mL brine, dried over $MgSO_4$, and concentrated. The liquid was purified by silica column (1–10% acetone in DCM) and dried under high vacuum overnight to afford product as a dark pink liquid (1.04 g, 4.21 mmol, 28%). Analytical data is in accordance with published data.[14]

^{1}H NMR (500 MHz, $CDCl_3$) δ 4.28 – 4.12 (m, 4H), 3.92 (d, J = 22.1 Hz, 2H), 3.06 (d, J = 1.8 Hz, 3H), 1.33 (t, J = 7.1 Hz, 6H).

^{13}C NMR (126 MHz, $CDCl_3$) δ 167.6 (d, J = 3.1 Hz), 164.3 (d, J = 9.6 Hz), 63.0 (d, J = 6.2 Hz), 33.8 (d, J = 135.2 Hz), 21.2, 16.3 (d, J = 6.2 Hz).

^{31}P NMR (203 MHz, $CDCl_3$) δ 20.5.

HRMS(ESI) Calculated m/z for $C_8H_{15}N_4O_3PNa^+$ $[M+Na]^+$: 269.07740 found: 269.07706

Synthesis of **tz6–tz8** and **tz16**:

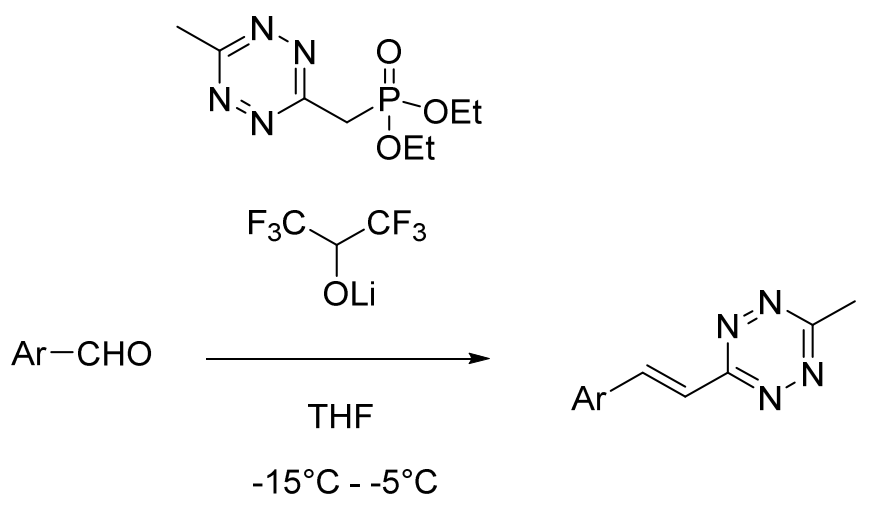

General: The method was adapted from a literature procedure.[14] To a 10 mL round bottom flask was added **S8** (1.1 eq.). The flask was evacuated and refilled with Ar 3 times, then dry THF (to final aldehyde concentration 0.1 M) was added to the flask. The solution was cooled to -15°C – -20°C, then 1 M solution of lithium hexafluoroisopropoxide (1.5 eq.) in THF was added dropwise. The bright pink solution turned dark pink to purple. Then the aldehyde (1 eq.) was added, and the mixture was stirred under Ar at -15°C – -5°C for 2 – 4 h. Upon TLC monitor showing complete consumption of aldehyde, the reaction was quenched with 2 mL saturated NH_4Cl solution, diluted with water, then extracted with EtOAc or DCM 3 times. The organic layer was washed with saturated $NaHCO_3$ and brine once before dried over $MgSO_4$ and evaporated. The crude material was suspended in Et_2O or 1:3 Et_2O/hexanes mixture, filtered and washed twice with same solvent to afford the product tetrazine from the precipitate.

(*E*)-8-methoxy-2-(2-(6-methyl-1,2,4,5-tetrazin-3-yl)vinyl)quinoline (**tz6**)

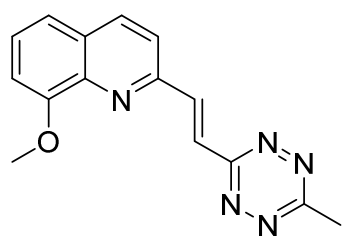

According to the general procedure, 8-methoxyquinoline-2-carbaldehyde (84.4 mg, 451 µmol) was reacted with **S8** (148 mg, 603 µmol) and lithium hexafluoroisopropoxide (650 µL, 650 µmol), to yield 102.4 mg (367 µmol, 81%) of **tz6** as a pink solid.

^{1}H NMR (500 MHz, $CDCl_3$) δ 8.61 (d, *J* = 16.2 Hz, 1H), 8.21 (d, *J* = 8.5 Hz, 1H), 8.03 (d, *J* = 16.2 Hz, 1H), 7.83 (d, *J* = 8.5 Hz, 1H), 7.50 (t, *J* = 7.9 Hz, 1H), 7.42 (dd, *J* = 8.2, 1.2 Hz, 1H), 7.10 (dd, *J* = 7.7, 1.2 Hz, 1H), 4.13 (s, 3H), 3.08 (s, 3H).

^{13}C NMR (126 MHz, $CDCl_3$) δ 166.7, 164.8, 155.8, 152.8, 141.0, 140.5, 136.9, 129.3, 127.9, 126.0, 120.8, 119.6, 108.5, 56.4, 21.4.

HRMS(ESI) Calculated m/z for $C_{15}H_{13}N_5ONa^+$ $[M+Na]^+$: 302.1012 found: 302.0999

(*E*)-7-methoxy-3-(2-(6-methyl-1,2,4,5-tetrazin-3-yl)vinyl)quinoline (**tz7**)

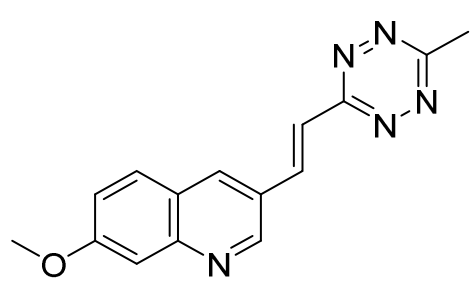

According to the general procedure, **S4** (80.8 mg, 432 µmol) was reacted with **S8** (177 mg, 476 µmol) and lithium hexafluoroisopropoxide (520 µL, 520 µmol), to yield 34.3 mg (123 µmol, 28%) of **tz7** as a pink solid.

^{1}H NMR (500 MHz, $CDCl_3$) δ 9.17 (d, *J* = 2.2 Hz, 1H), 8.43 (d, *J* = 16.4 Hz, 1H), 8.31 (d, *J* = 2.2 Hz, 1H), 7.78 (d, *J* = 9.0 Hz, 1H), 7.62 (d, *J* = 16.3 Hz, 1H), 7.45 (d, *J* = 2.5 Hz, 1H), 7.25 (dd, *J* = 8.9, 2.4 Hz, 1H), 3.99 (s, 3H), 3.08 (s, 3H).

^{13}C NMR (126 MHz, $CDCl_3$) δ 166.6, 164.7, 162.0, 150.6, 150.0, 137.9, 134.9, 129.6, 126.3, 123.1, 121.3, 120.9, 107.7, 55.8, 21.4.

HRMS(ESI) Calculated m/z for $C_{15}H_{14}N_5O^+$ $[M+H]^+$: 280.11929 found: 280.11902

(*E*)-3-(2-(6-methyl-1,2,4,5-tetrazin-3-yl)vinyl)-7-(4-methylpiperazin-1-yl)quinoline (**tz8**)

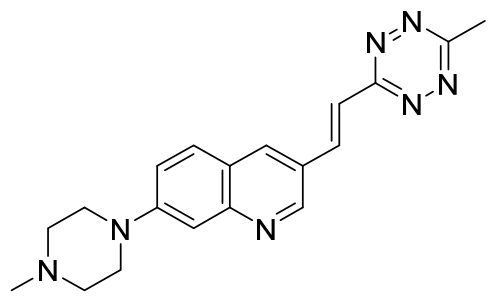

According to the general procedure, **S5** (82.3 mg, 322 µmol) was reacted with **S8** (87.3 mg, 476 µmol) and lithium hexafluoroisopropoxide (500 µL, 500 µmol), to yield 79.3 mg (228 µmol, 71%) of **tz8** as a pink solid.

^{1}H NMR (500 MHz, $CDCl_3$) δ 9.09 (d, *J* = 2.2 Hz, 1H), 8.39 (d, *J* = 16.3 Hz, 1H), 8.22 (d, *J* = 2.2 Hz, 1H), 7.72 (d, *J* = 9.0 Hz, 1H), 7.57 (d, *J* = 16.3 Hz, 1H), 7.36 (d, *J* = 2.4 Hz, 1H), 7.34 (dd, *J* = 9.0, 2.5 Hz, 1H), 3.48 – 3.41 (m, 4H), 3.06 (s, 3H), 2.64 – 2.60 (m, 4H), 2.38 (s, 3H).

^{13}C NMR (126 MHz, $CDCl_3$) δ 166.4, 164.8, 152.9, 150.6, 150.2, 138.2, 134.6, 129.2, 125.4, 121.8, 120.3, 119.5, 110.5, 54.9, 48.3, 46.3, 21.3.

HRMS(ESI) Calculated m/z for $C_{19}H_{22}N_7^+$ $[M+H]^+$: 348.1931 found: 348.1940

(*E*)-*N*,*N*-dimethyl-2-(2-(6-methyl-1,2,4,5-tetrazin-3-yl)vinyl)quinolin-7-amine (**tz16**)

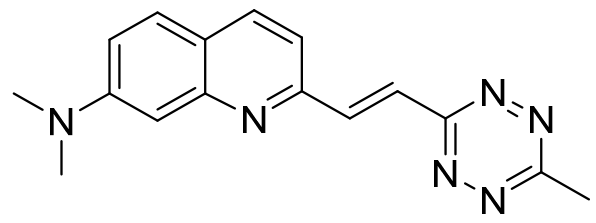

According to the general procedure, **S7** (40.0 mg, 200 µmol) was reacted with **S8** (54.1 mg, 220 µmol) and lithium hexafluoroisopropoxide (300 µL, 300 µmol), to yield 45.1 mg (154 µmol, 88%) of **tz16** as a pink solid.

^{1}H NMR (500 MHz, $CDCl_3$) δ 8.46 (d, *J* = 16.0 Hz, 1H), 8.08 – 8.01 (m, 2H), 7.67 (d, *J* = 8.8 Hz, 1H), 7.46 (d, *J* = 8.2 Hz, 1H), 7.24 – 7.17 (m, 2H), 3.14 (s, 6H), 3.08 (s, 3H), 2.17 (s, 2H).

^{13}C NMR (126 MHz, $CDCl_3$) δ 166.6, 164.9, 153.8, 151.8, 150.4, 141.3, 136.3, 128.3, 125.3, 121.1, 117.3, 117.2, 107.0, 40.6, 21.4.

HRMS(ESI) Calculated m/z for $C_{16}H_{17}N_6^+$ $[M+H]^+$: 293.1509 found: 293.1503

NMR spectra

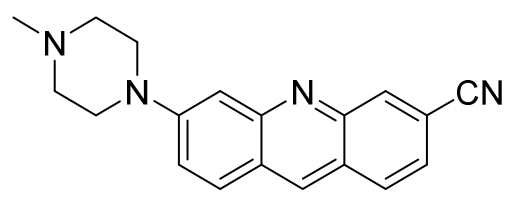

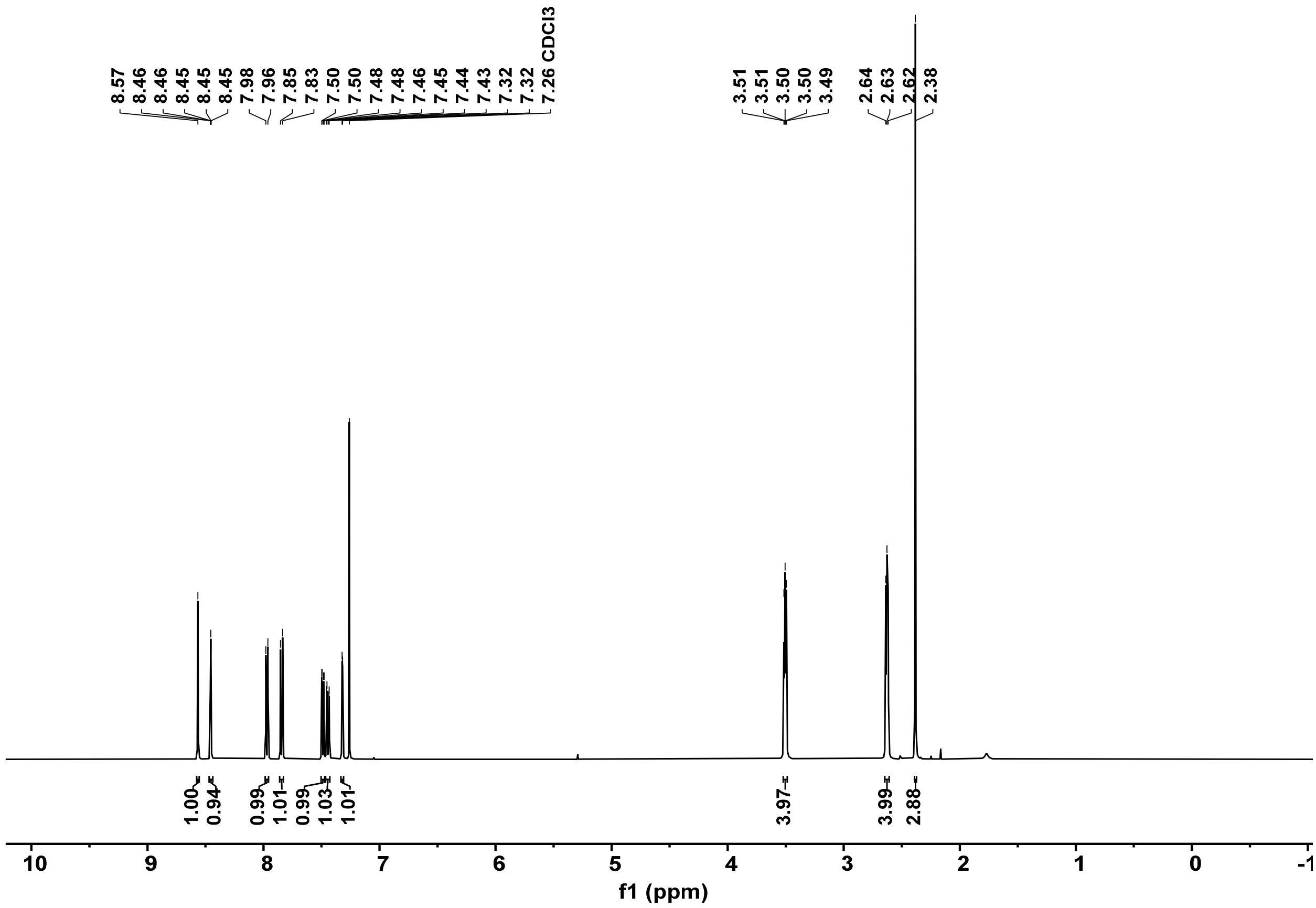

^{1}H ($CDCl_3$)

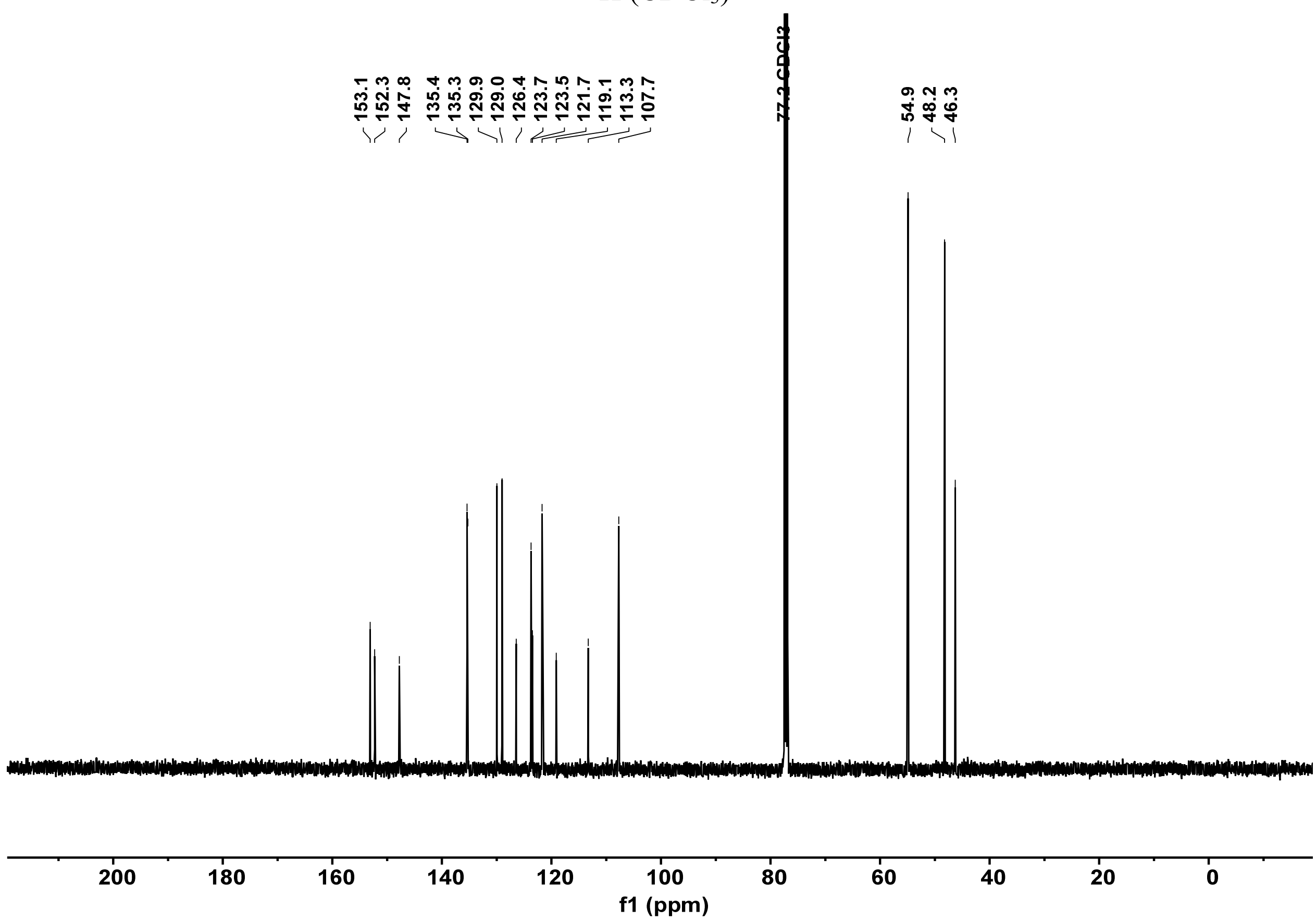

^{13}C ($CDCl_3$)

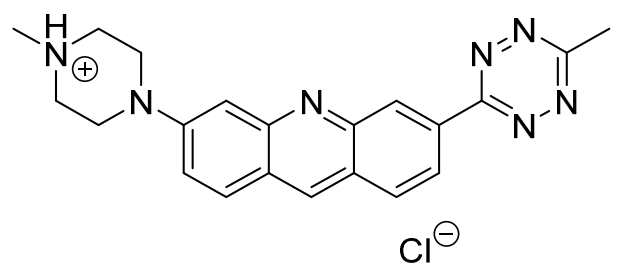

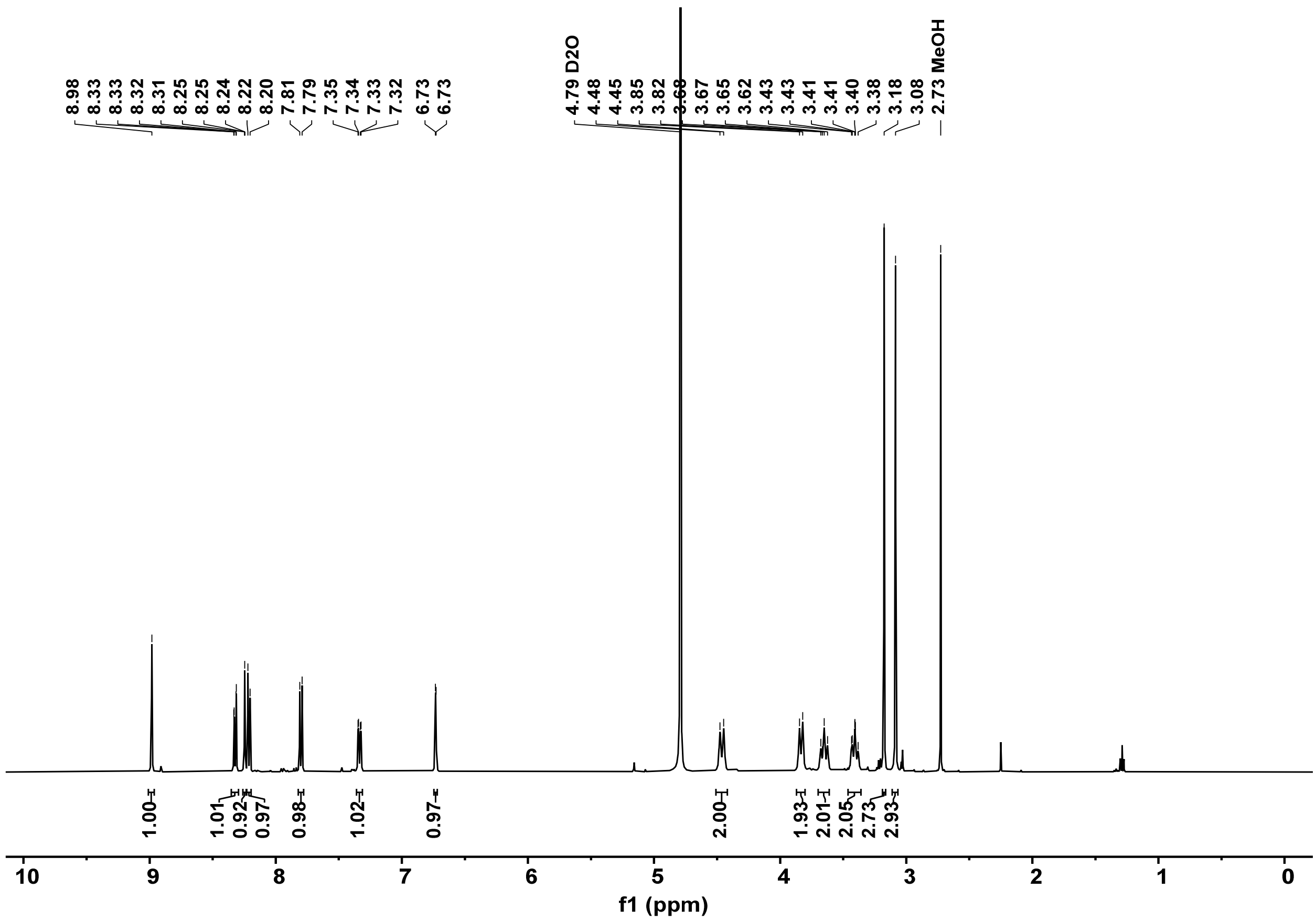

^{1}H (D_2O)

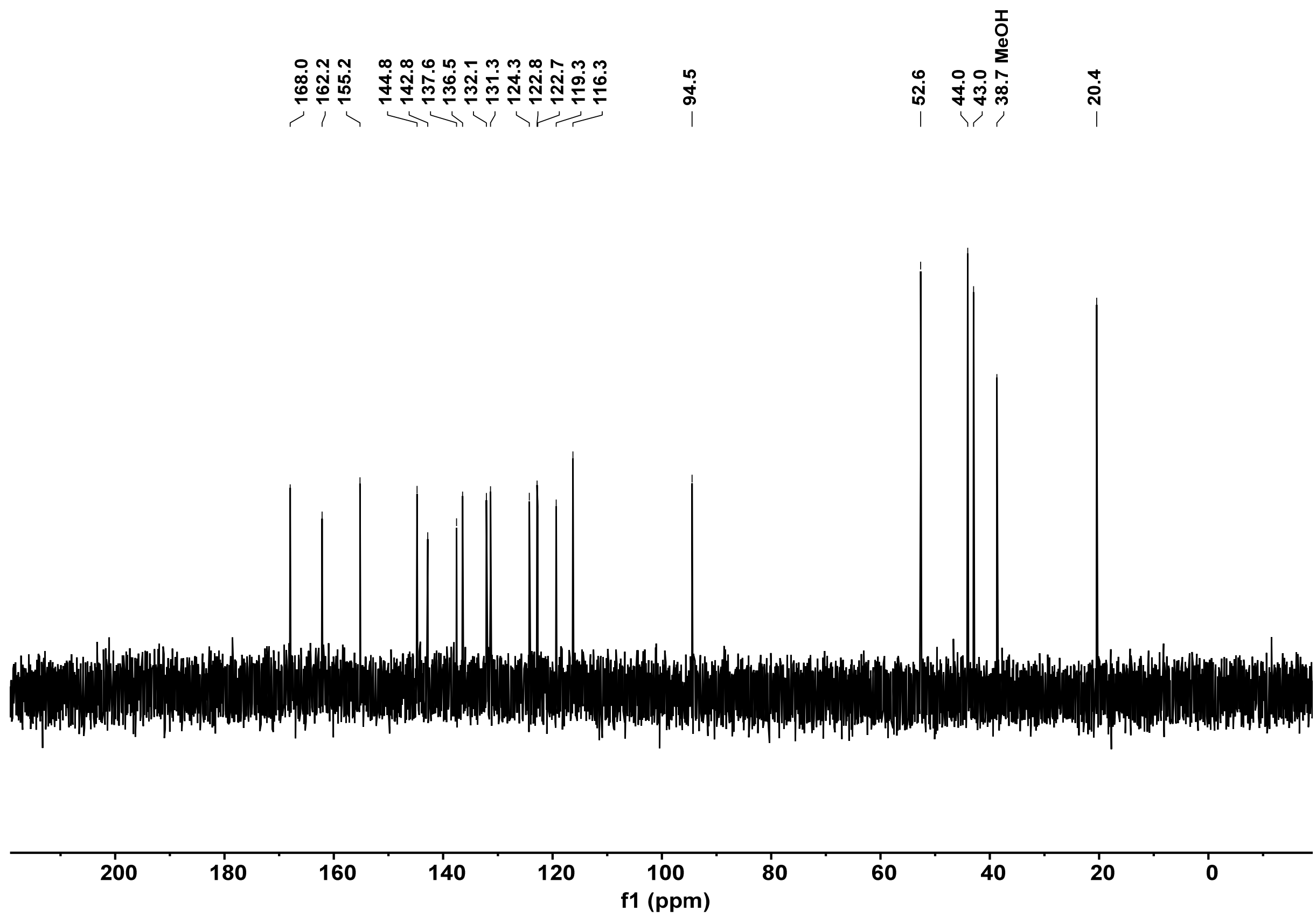

^{13}C (D_2O)

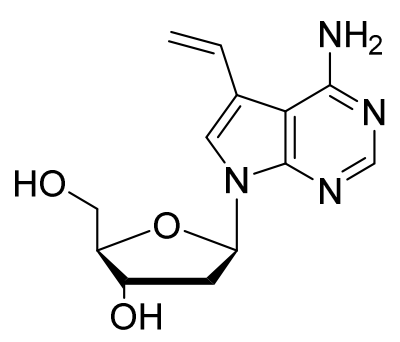

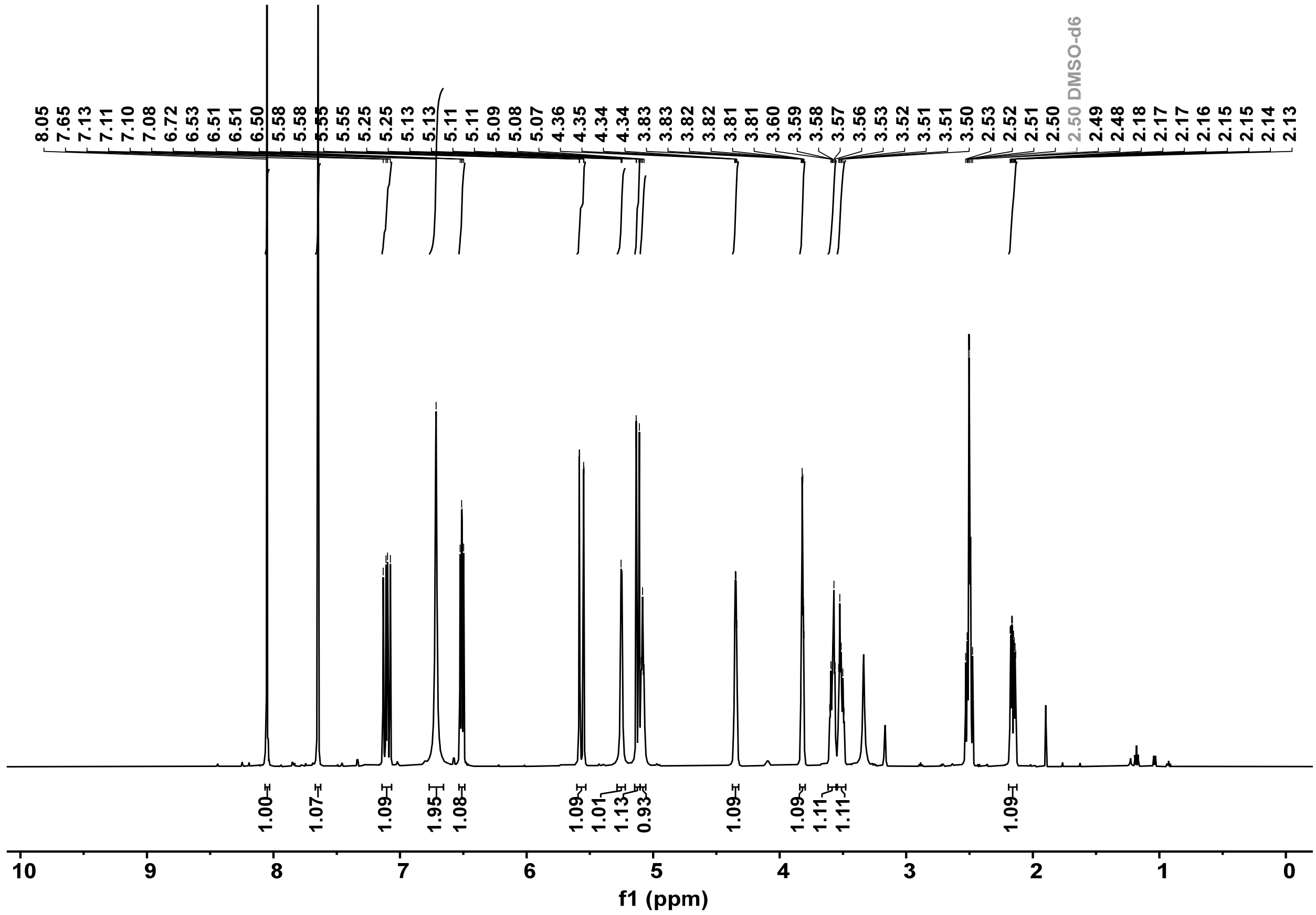

[1]H (DMSO-d_6)

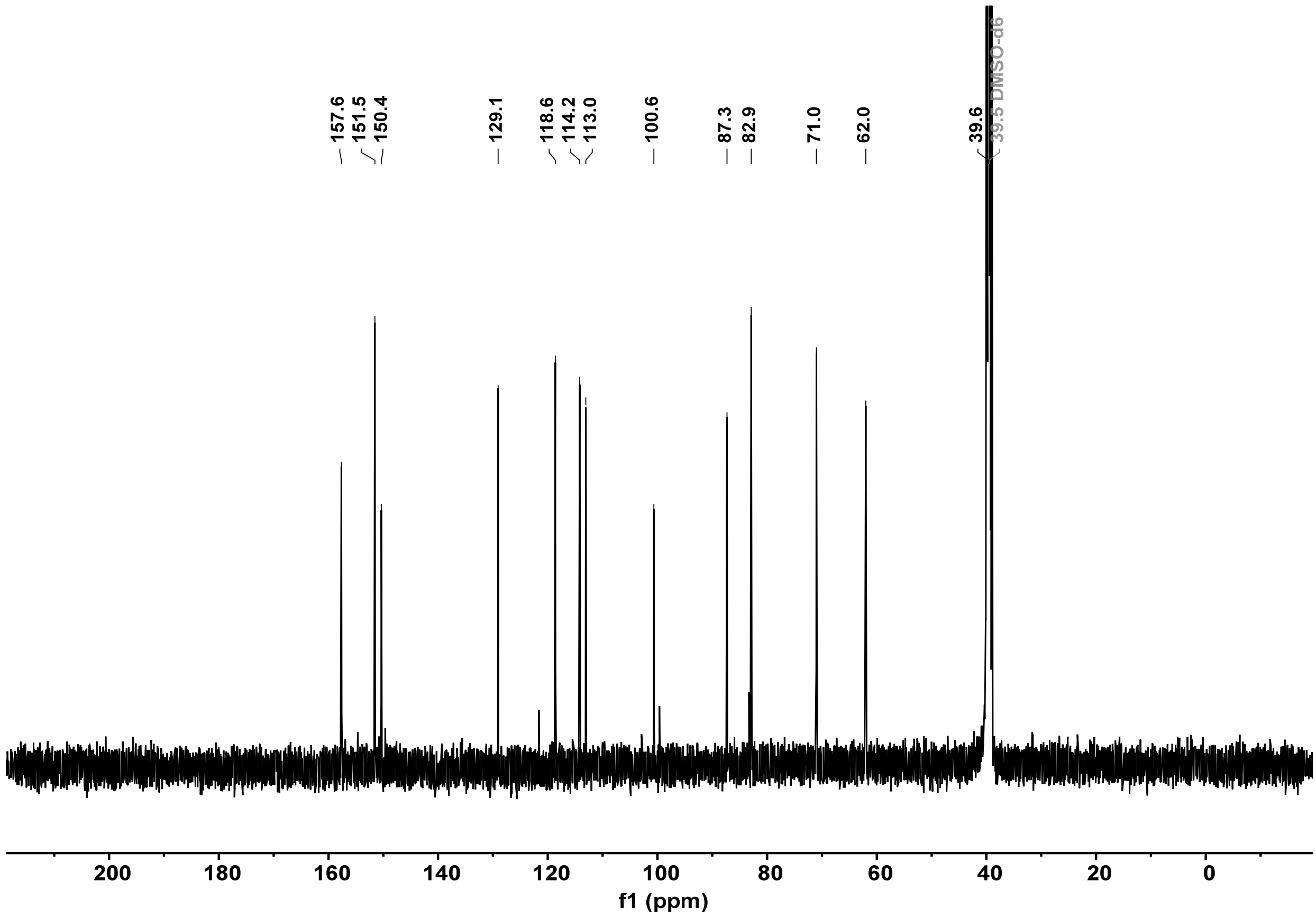

[13]C (DMSO-d_6)

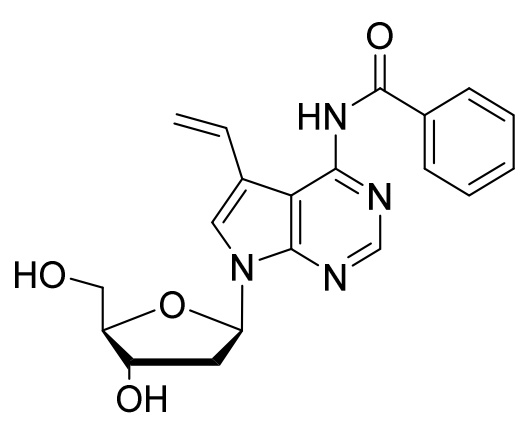

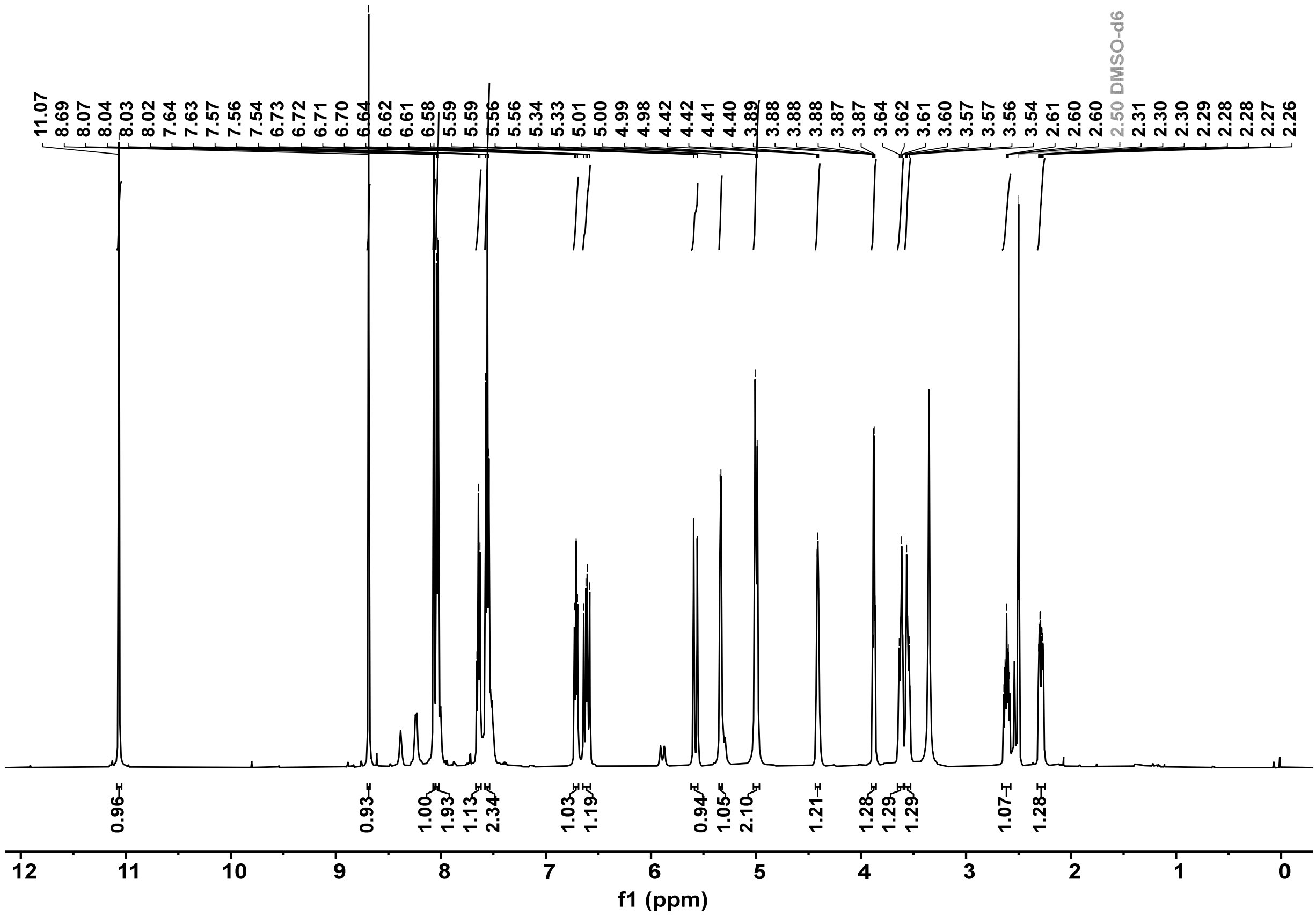

^{1}H (DMSO-d_6)

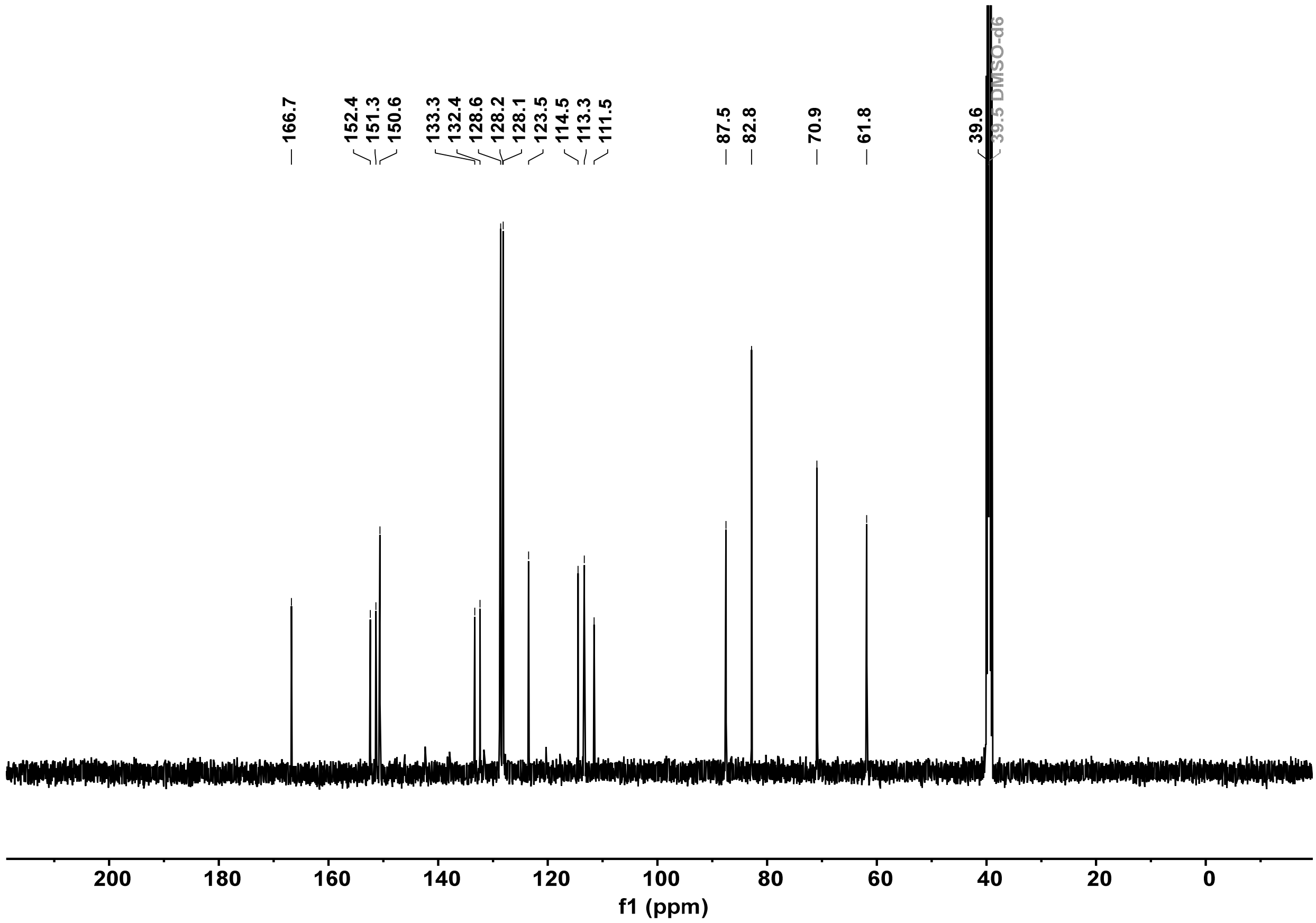

^{13}C (DMSO-d_6)

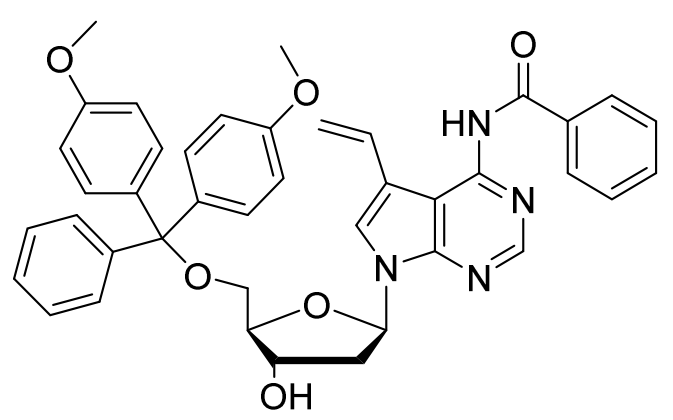

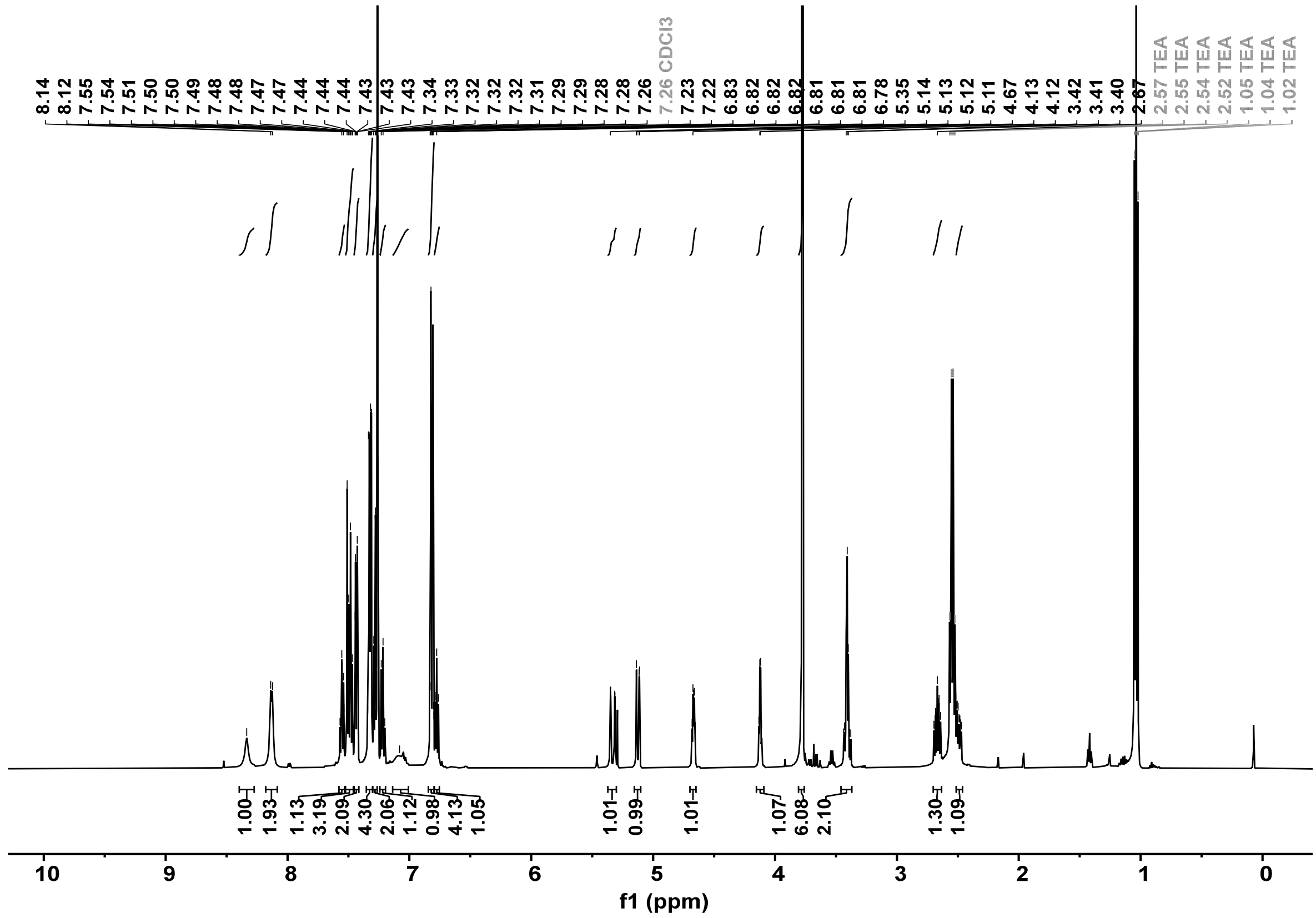

^{1}H ($CDCl_3$)

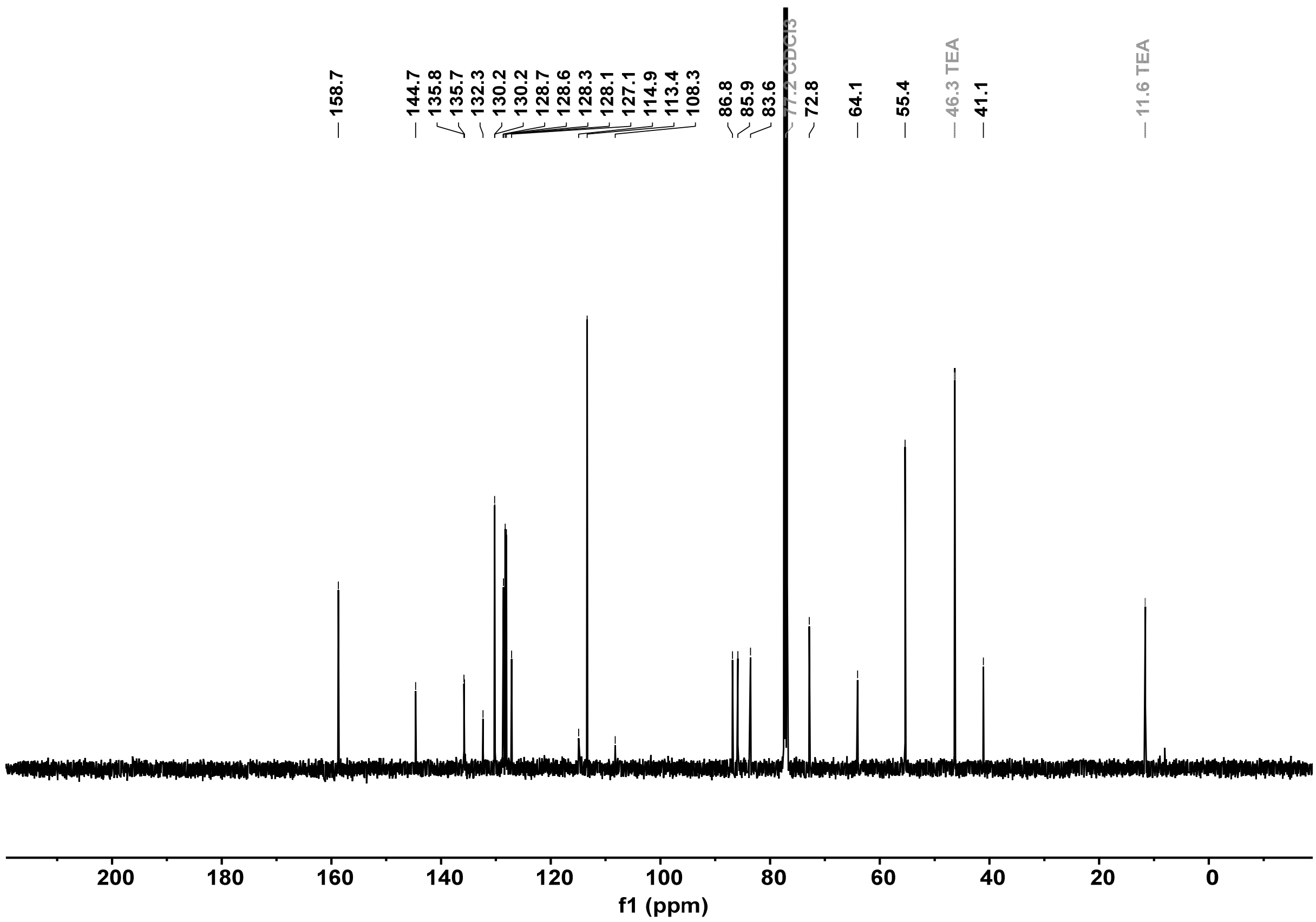

^{13}C ($CDCl_3$)

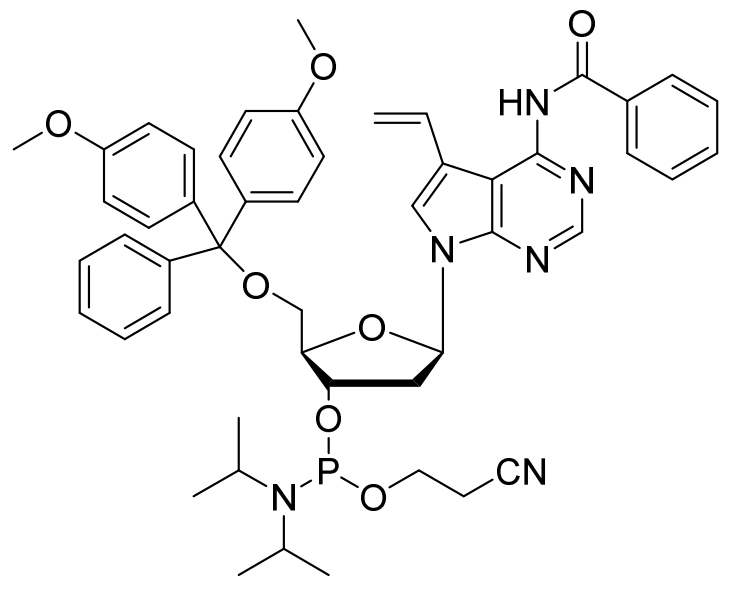

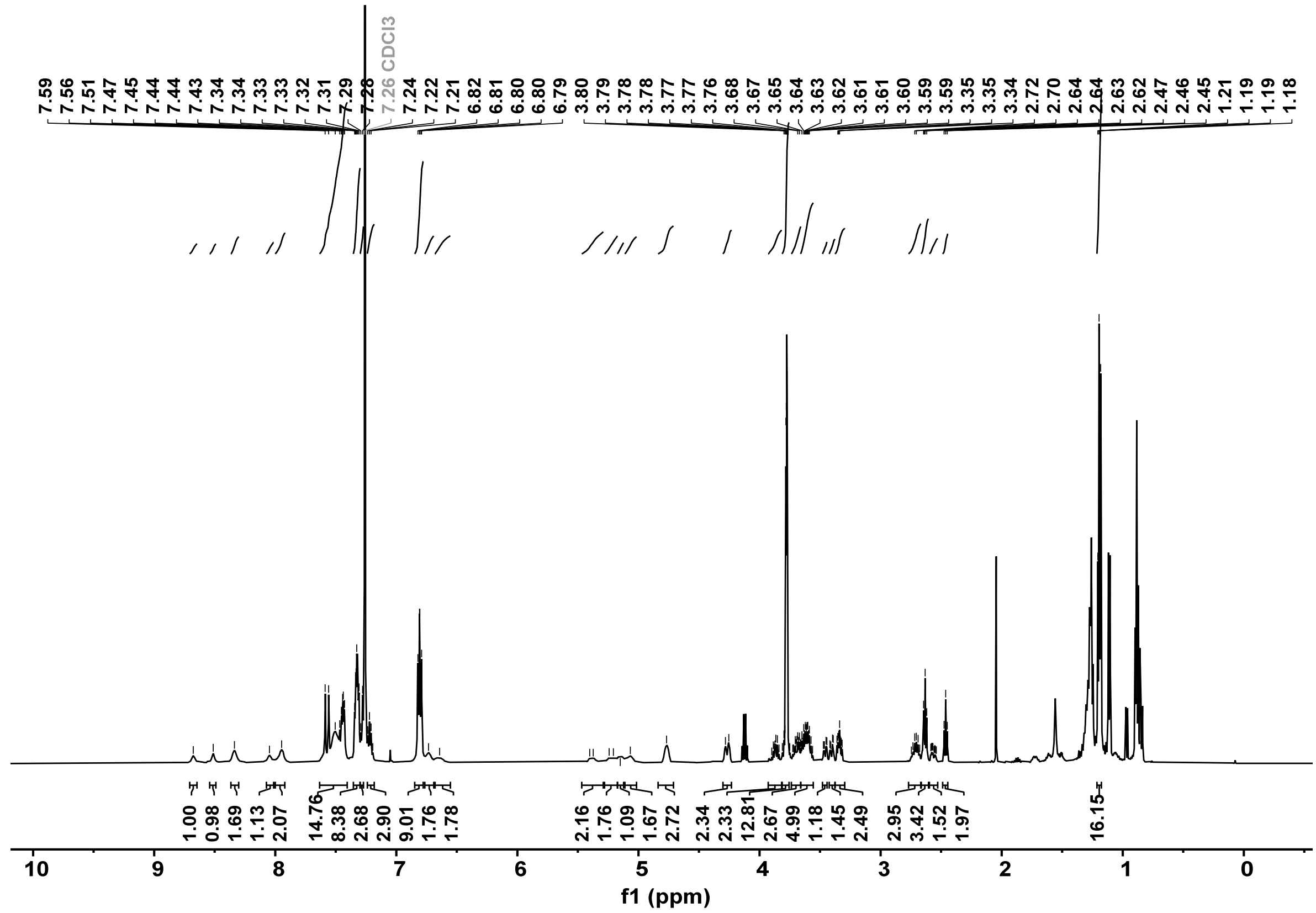

^{1}H ($CDCl_3$)

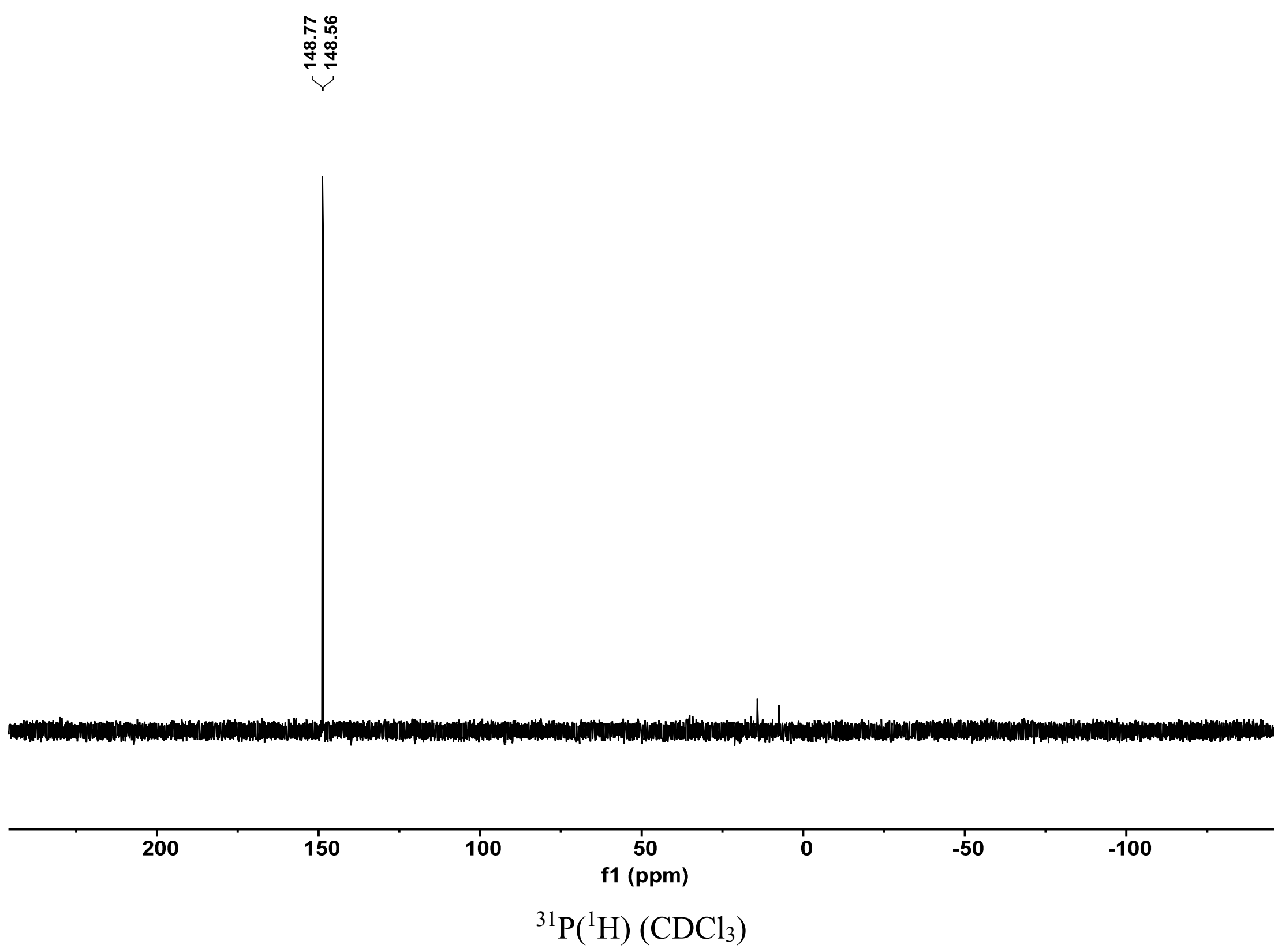

$^{31}P(^{1}H)$ ($CDCl_3$)

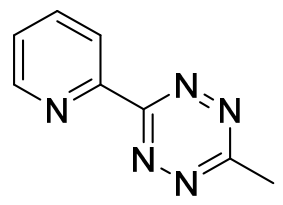

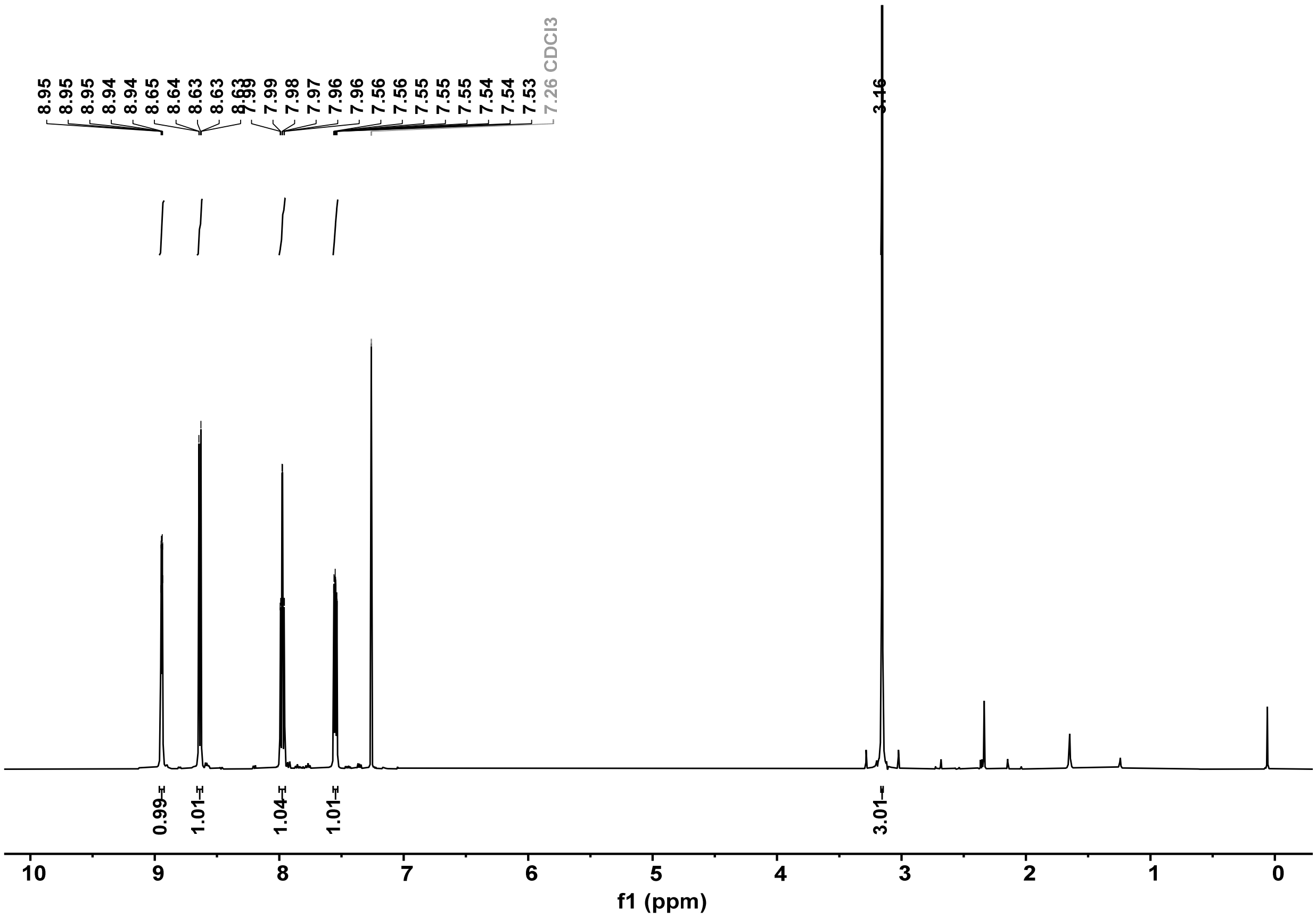

^{1}H (CDCl$_3$)

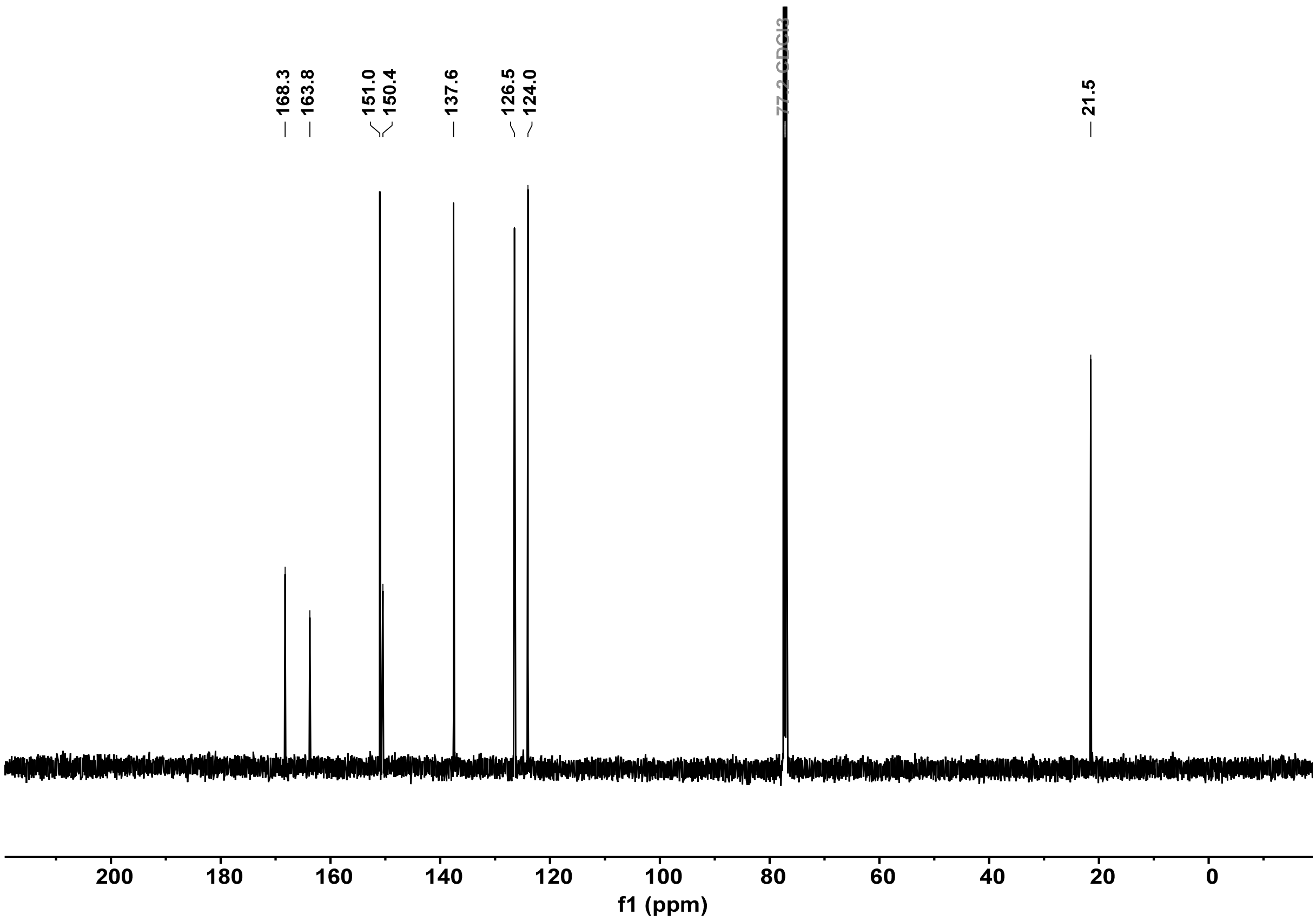

^{13}C (CDCl$_3$)

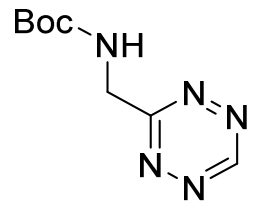

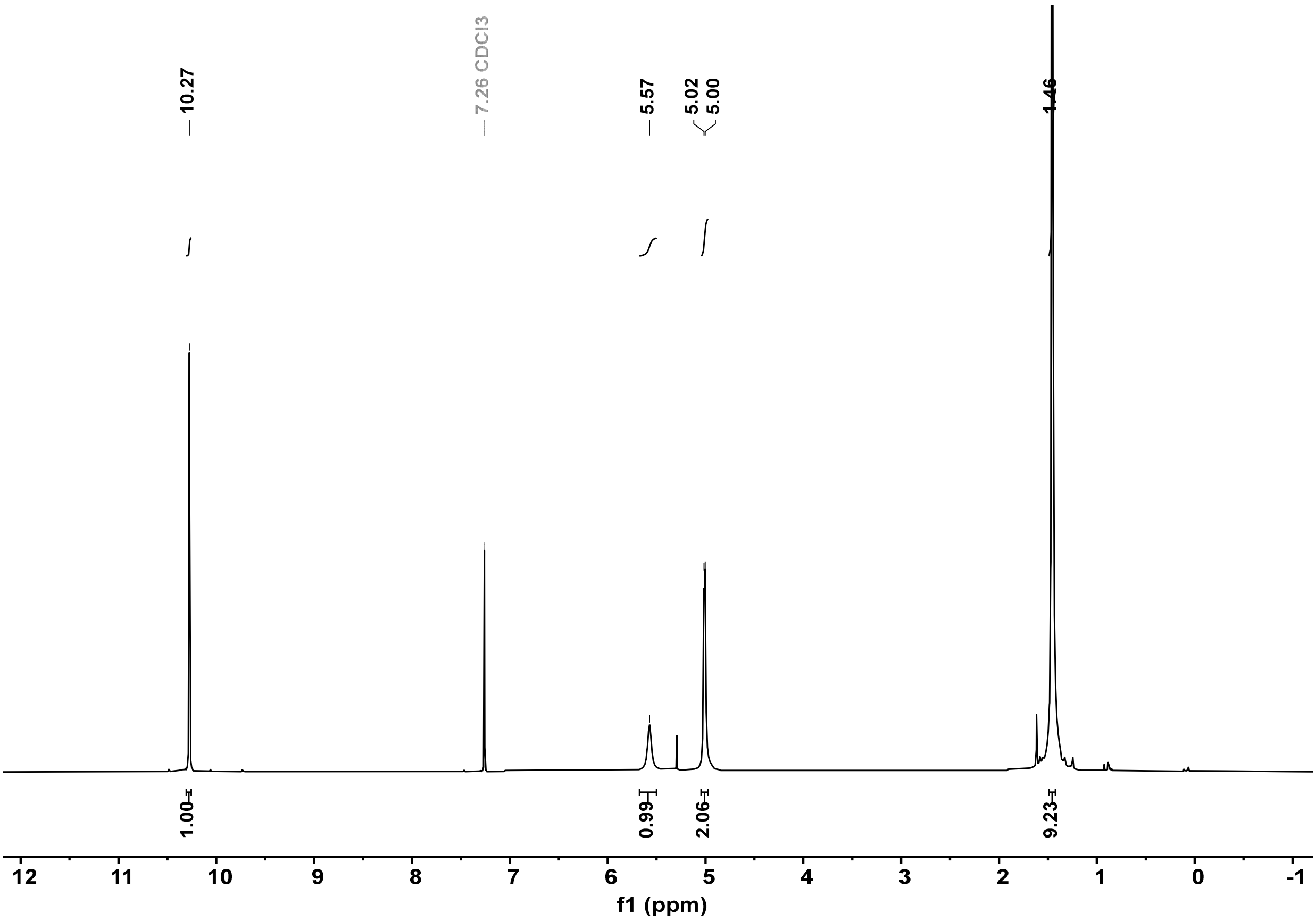

^{1}H ($CDCl_3$)

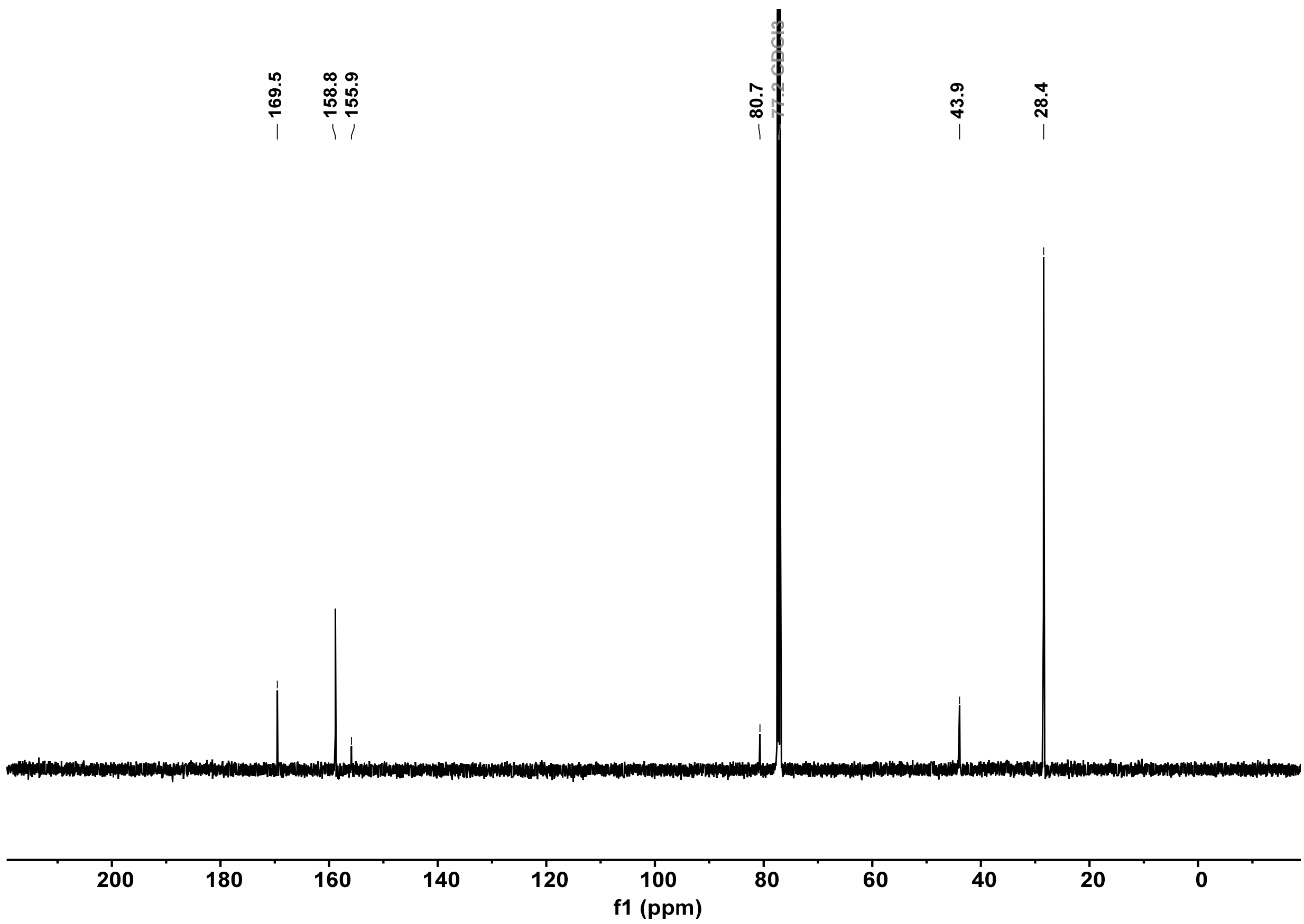

^{13}C ($CDCl_3$)

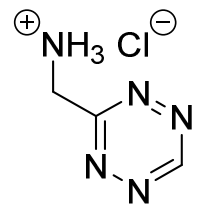

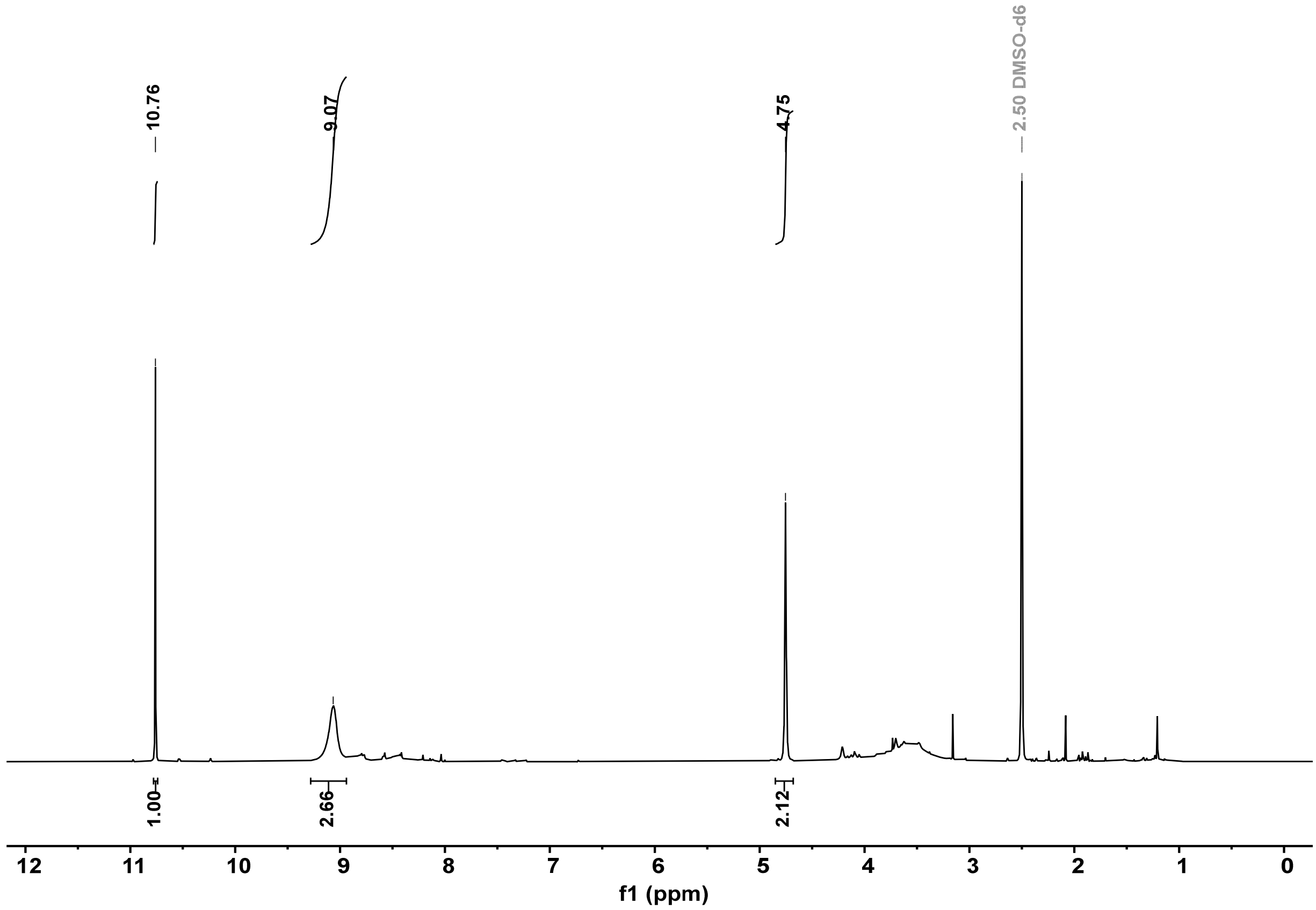

^{1}H (DMSO-d_6)

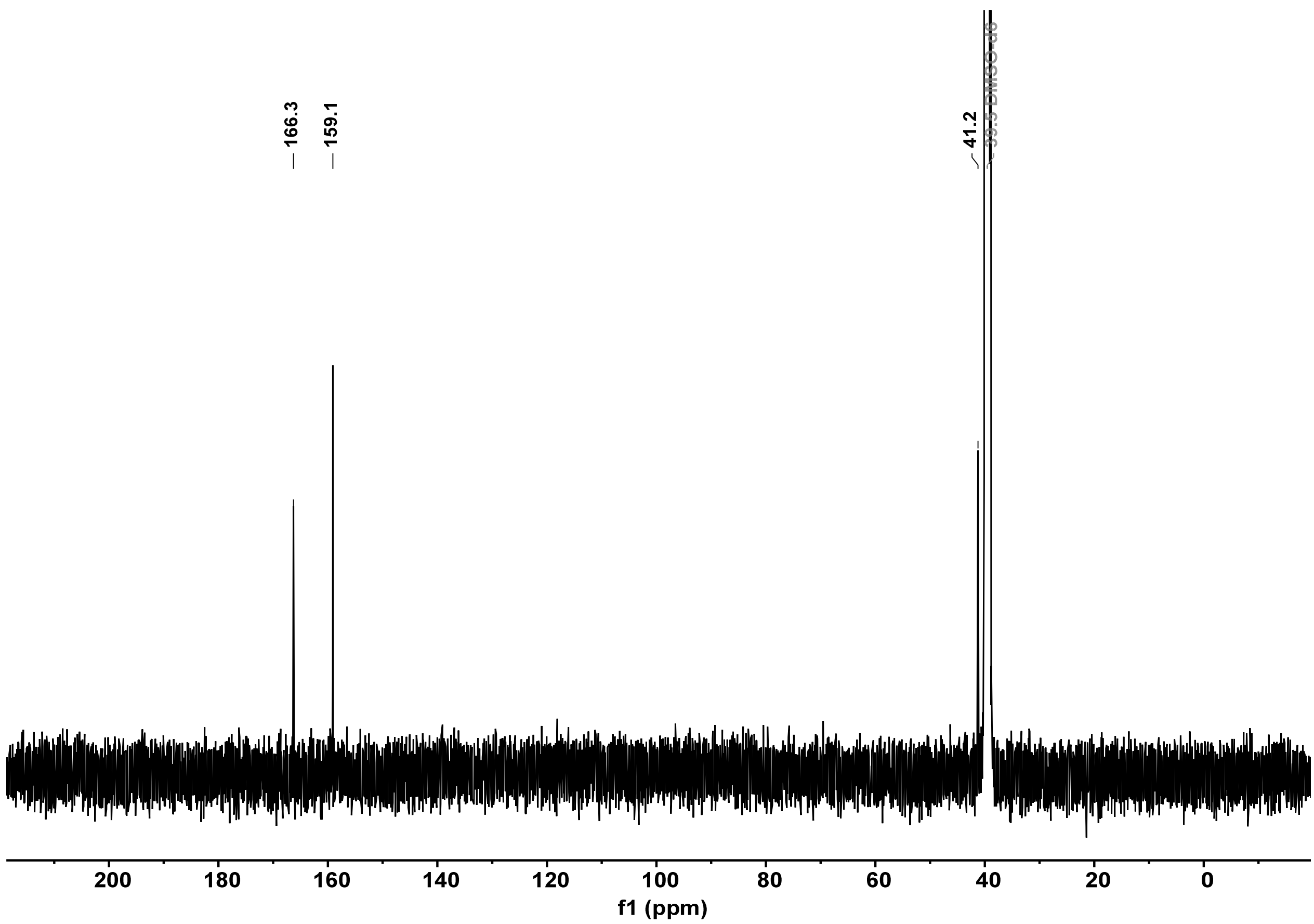

^{13}C (DMSO-d_6)

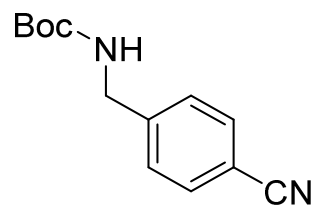

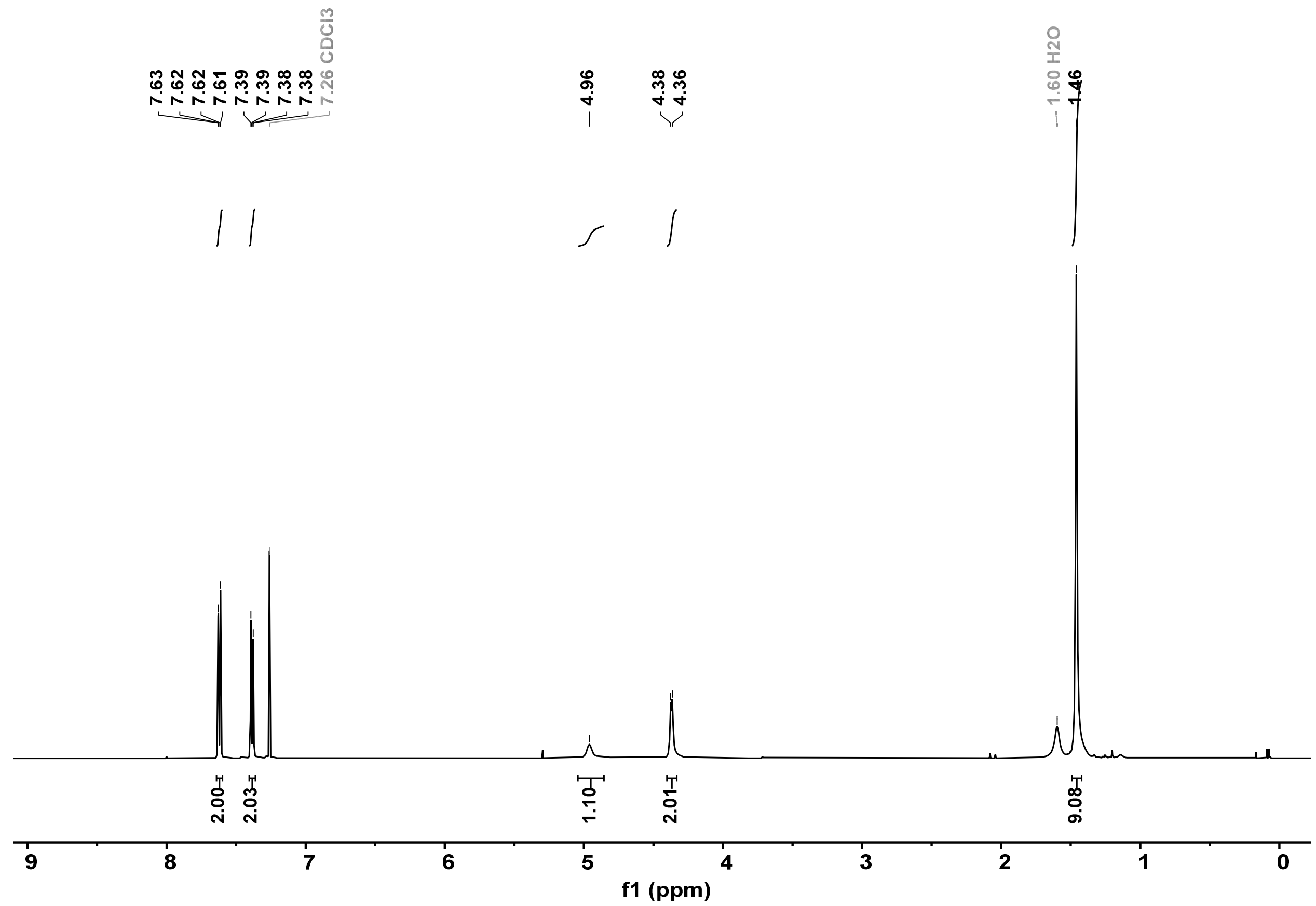

^{1}H ($CDCl_3$)

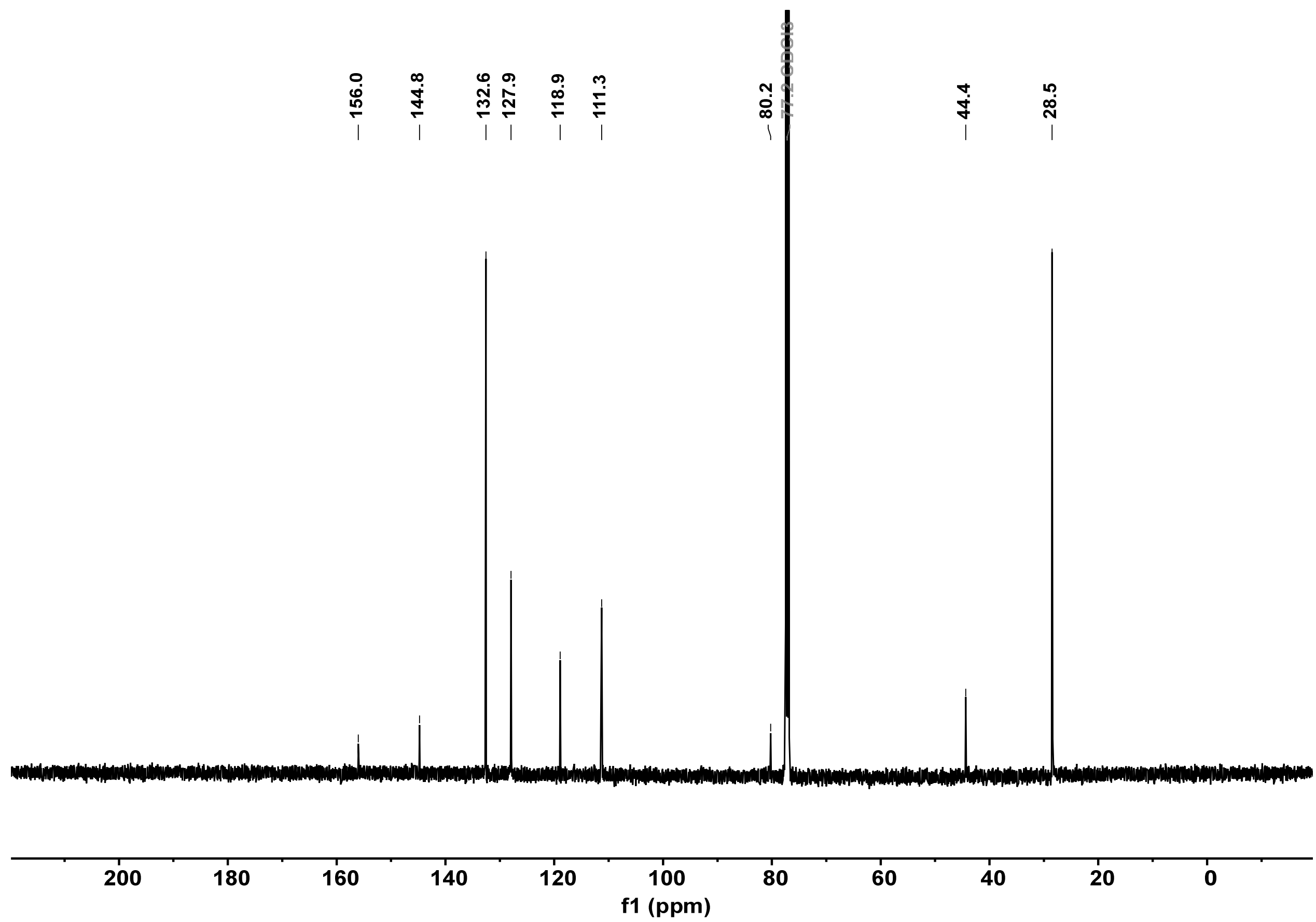

^{13}C ($CDCl_3$)

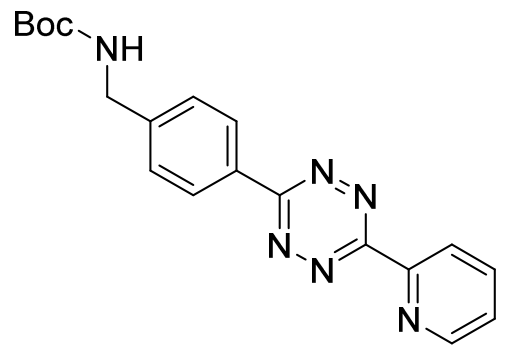

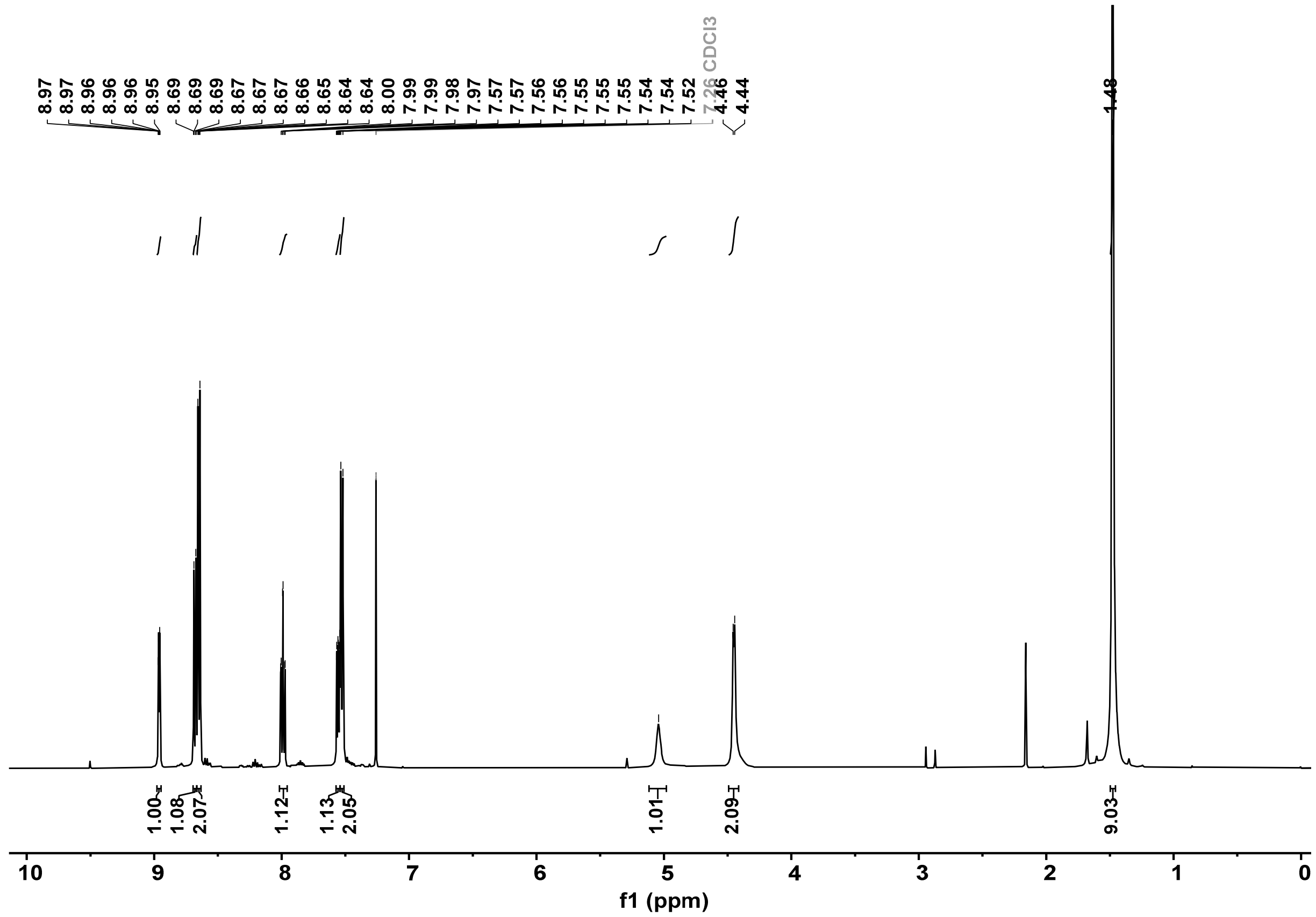

^{1}H ($CDCl_3$)

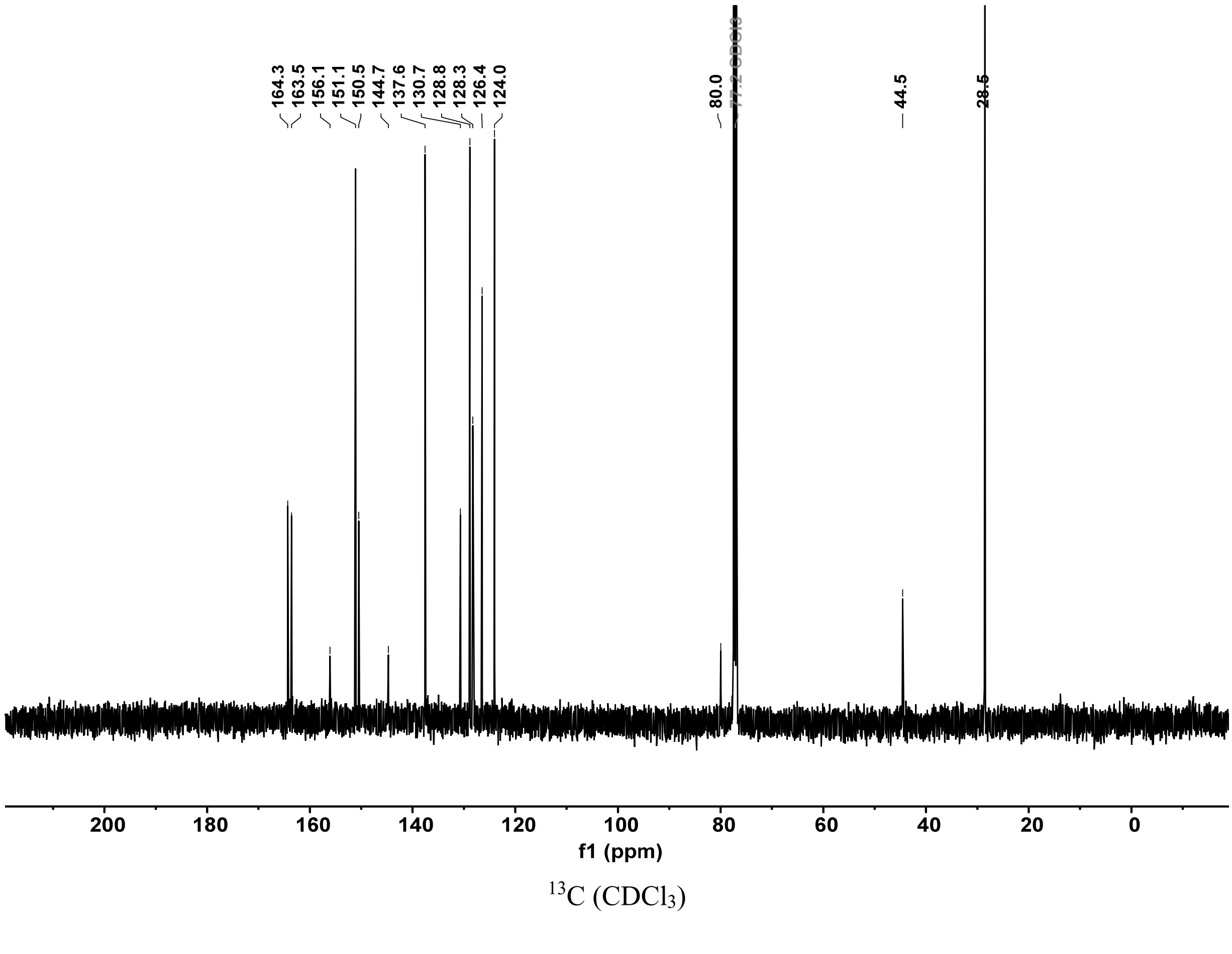

^{13}C ($CDCl_3$)

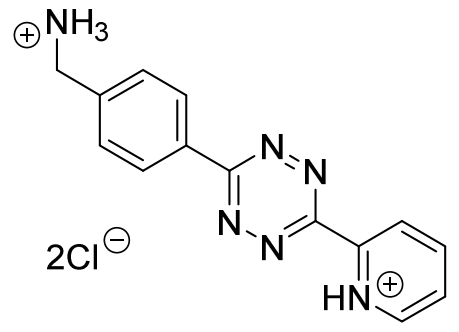

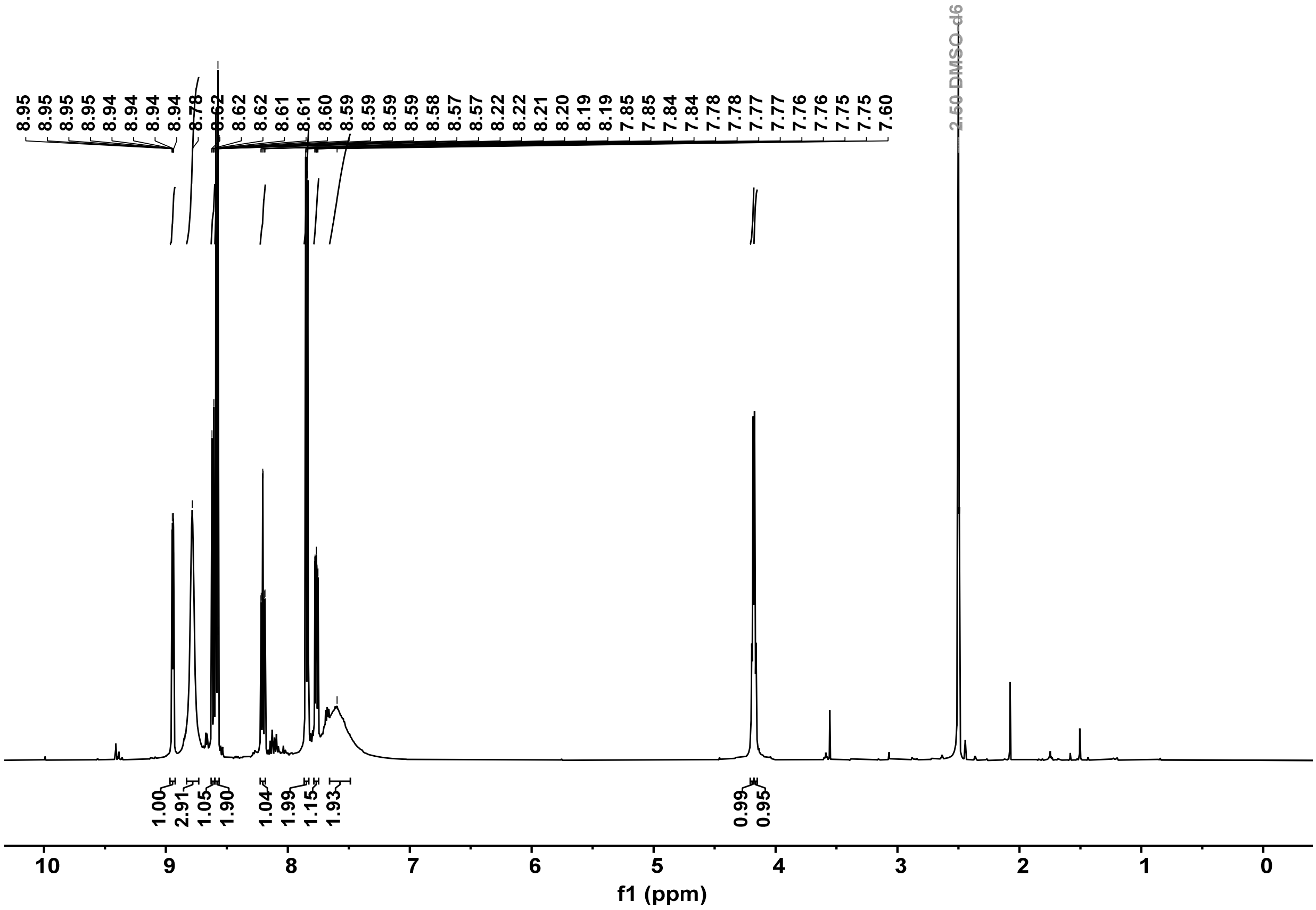

^{1}H (DMSO-d_6)

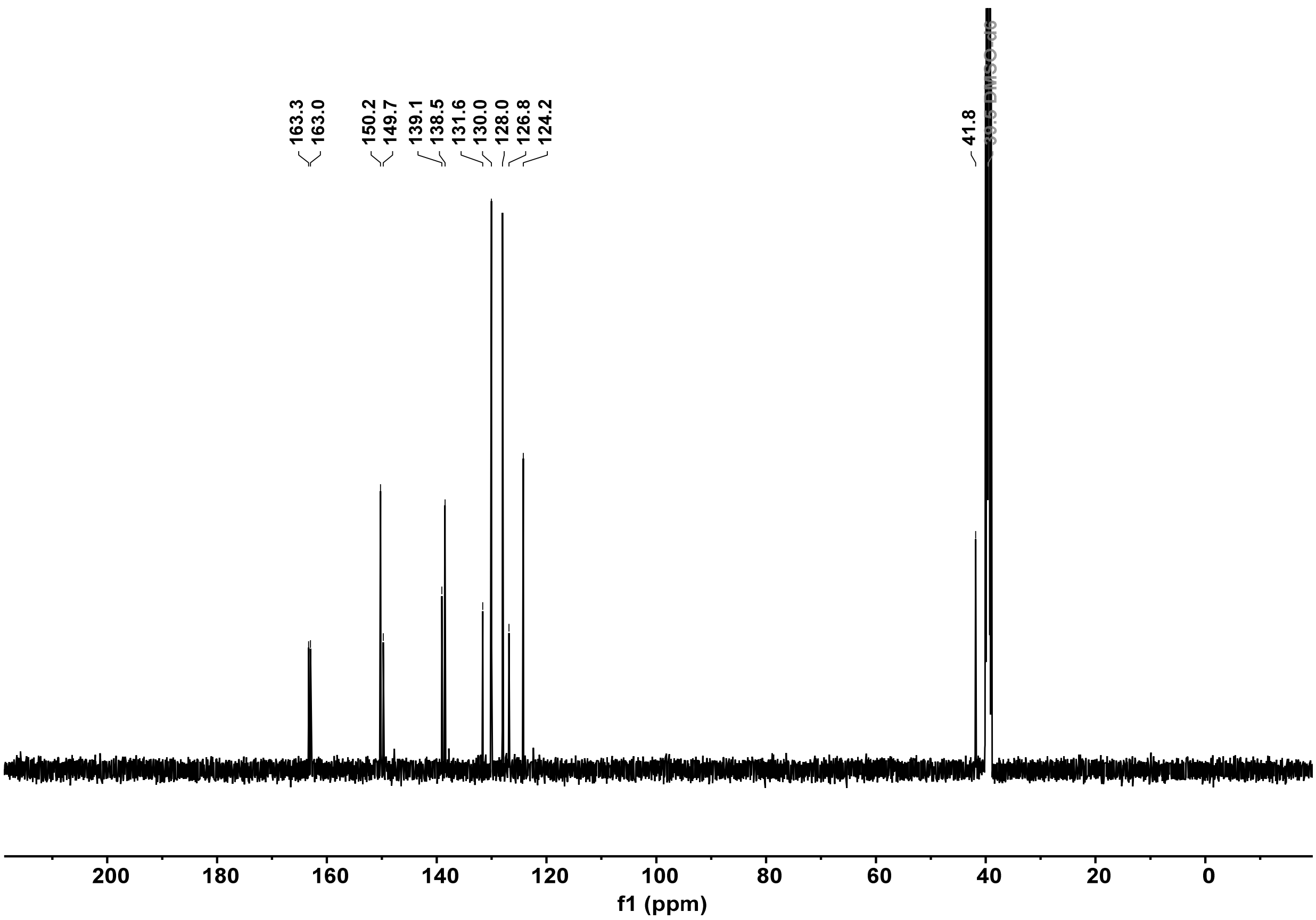

^{13}C (DMSO-d_6)

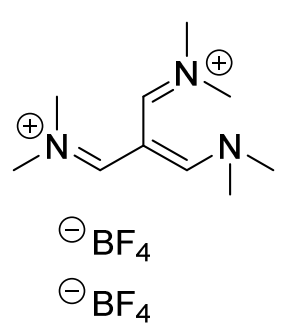

N
N
N
BF4
BF4

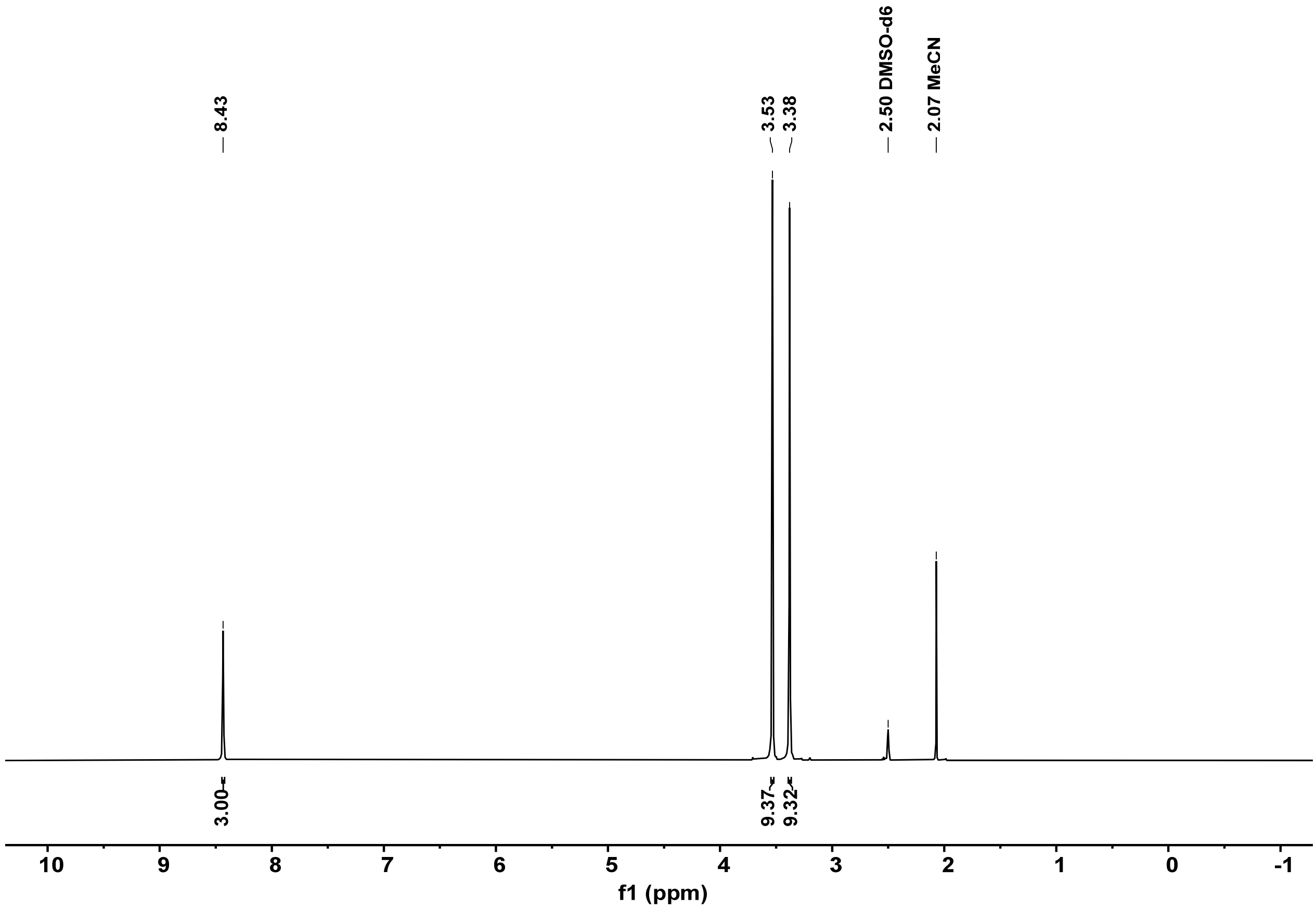

8.43
3.53
3.38
2.50 DMSO-d6
2.07 MeCN
3.00
9.37
9.32
10
9
8
7
6
5
4
3
2
1
0
-1
f1 (ppm)

^{1}H (DMSO-d_6)

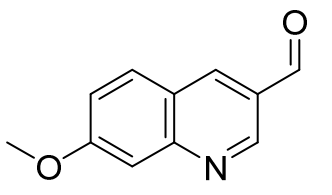

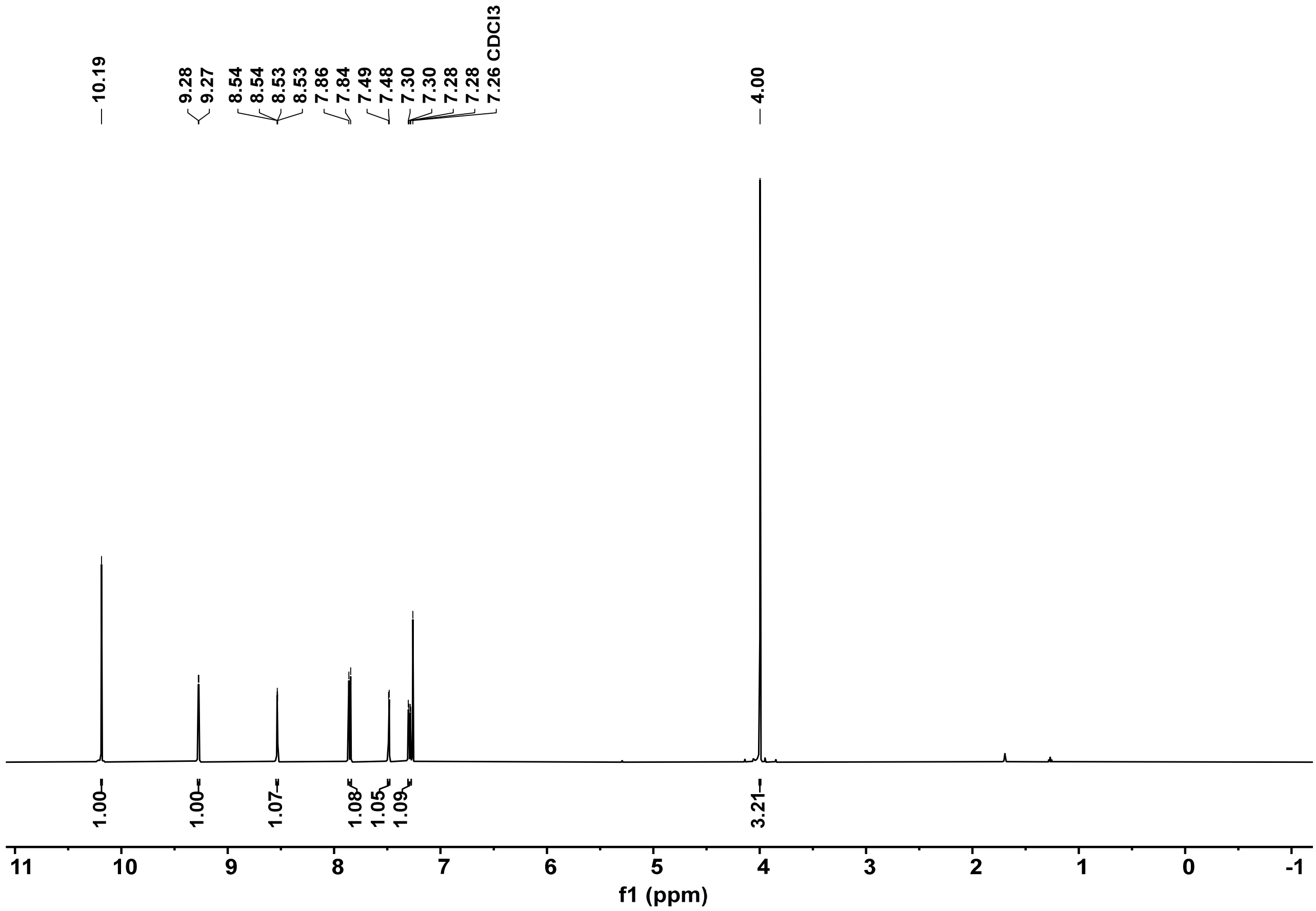

^{1}H ($CDCl_3$)

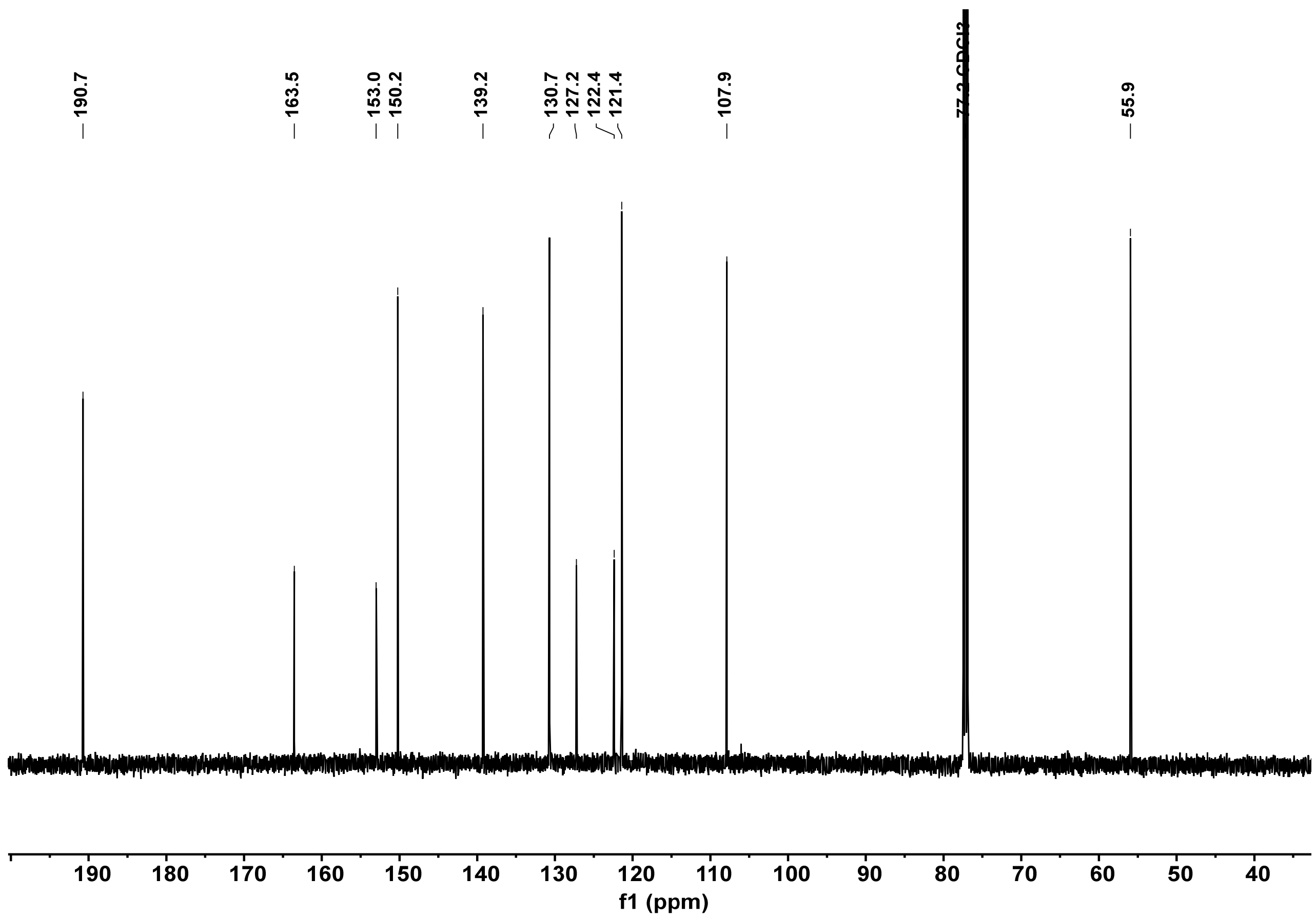

^{13}C ($CDCl_3$)

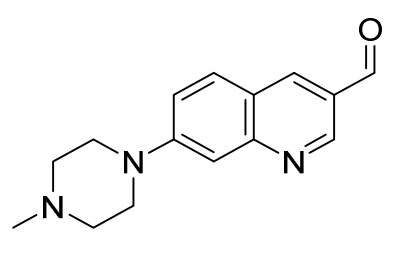

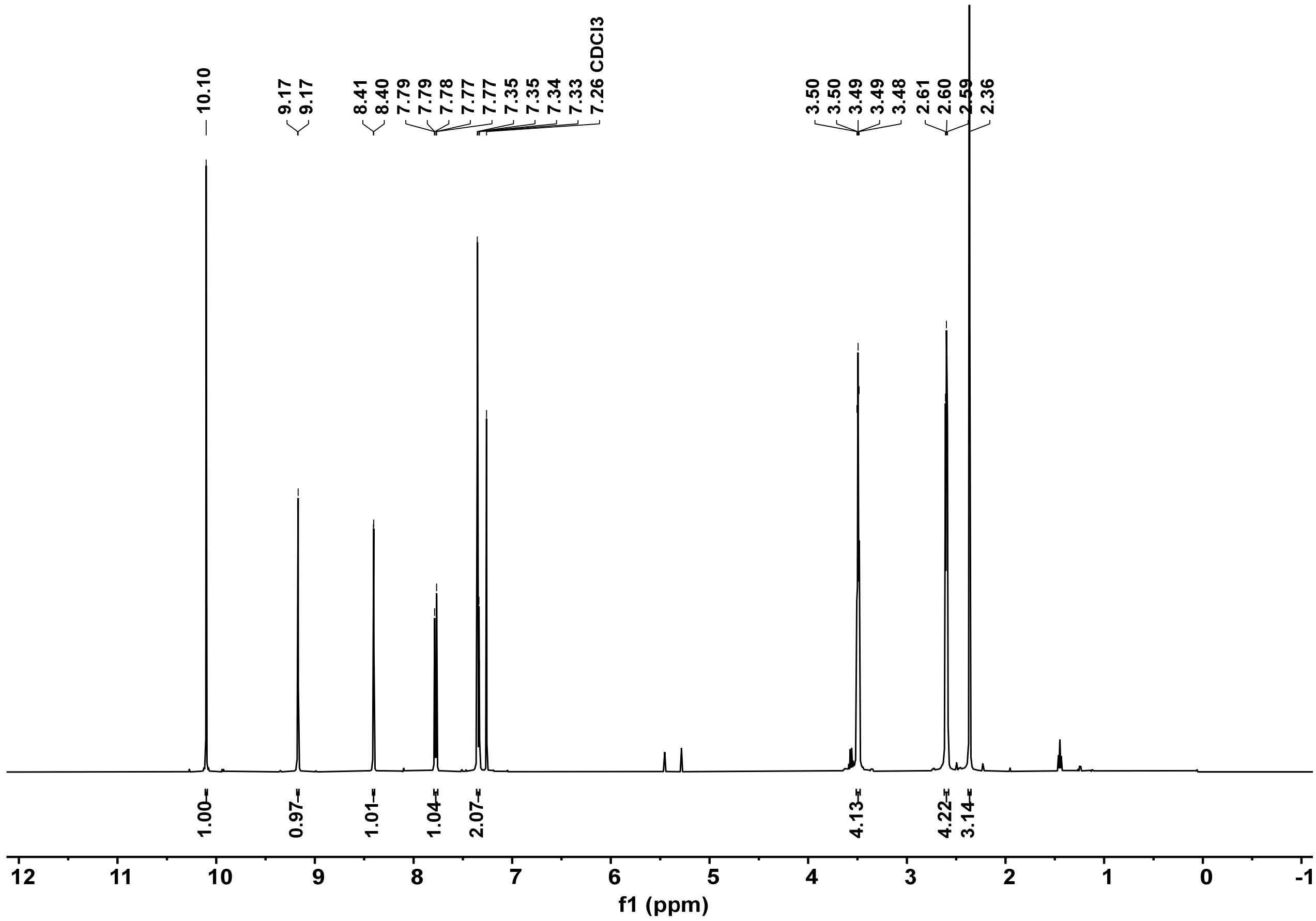

^{1}H ($CDCl_3$)

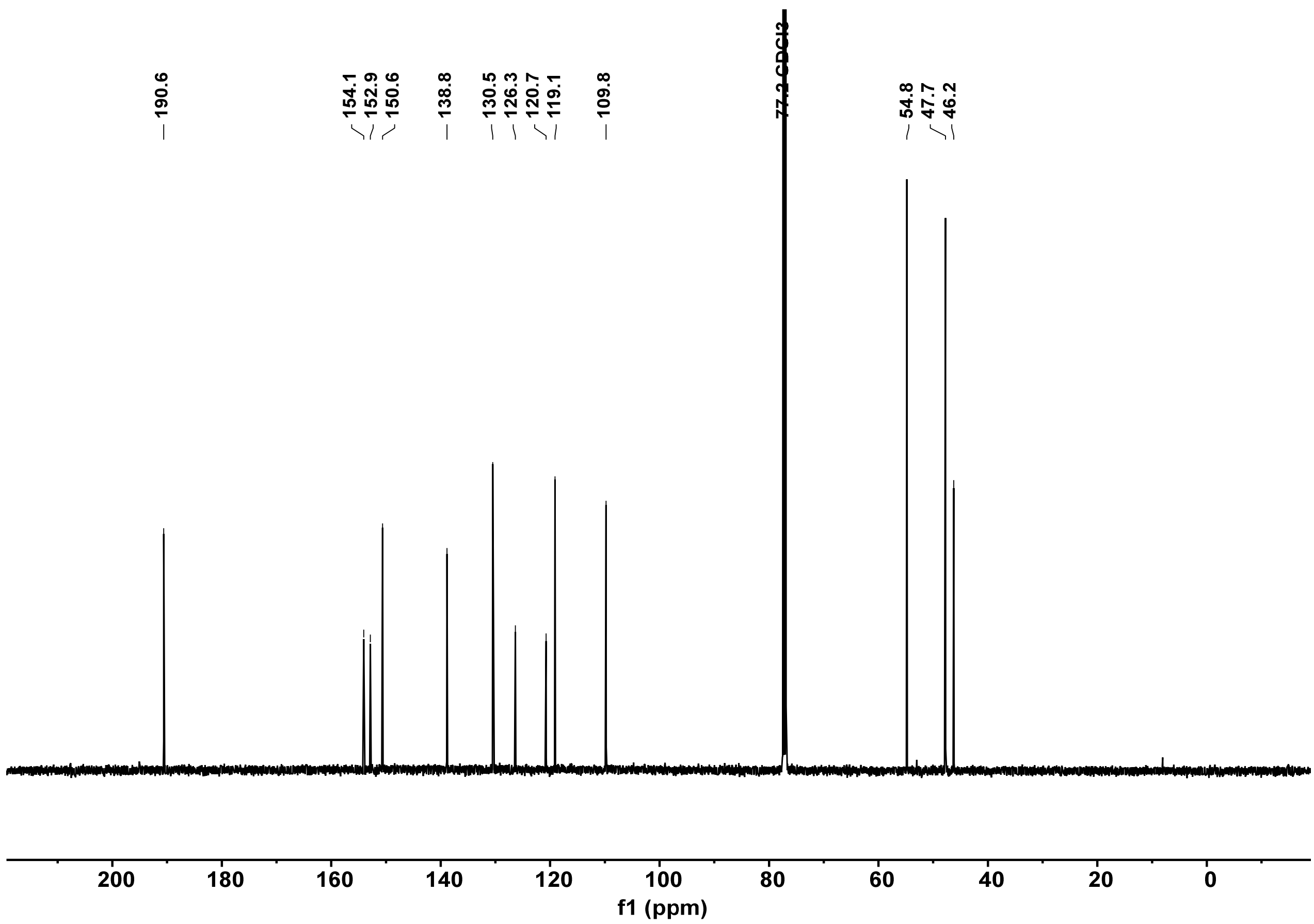

^{13}C ($CDCl_3$)

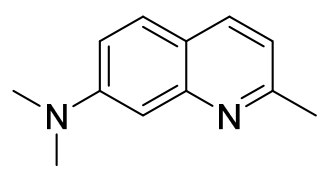

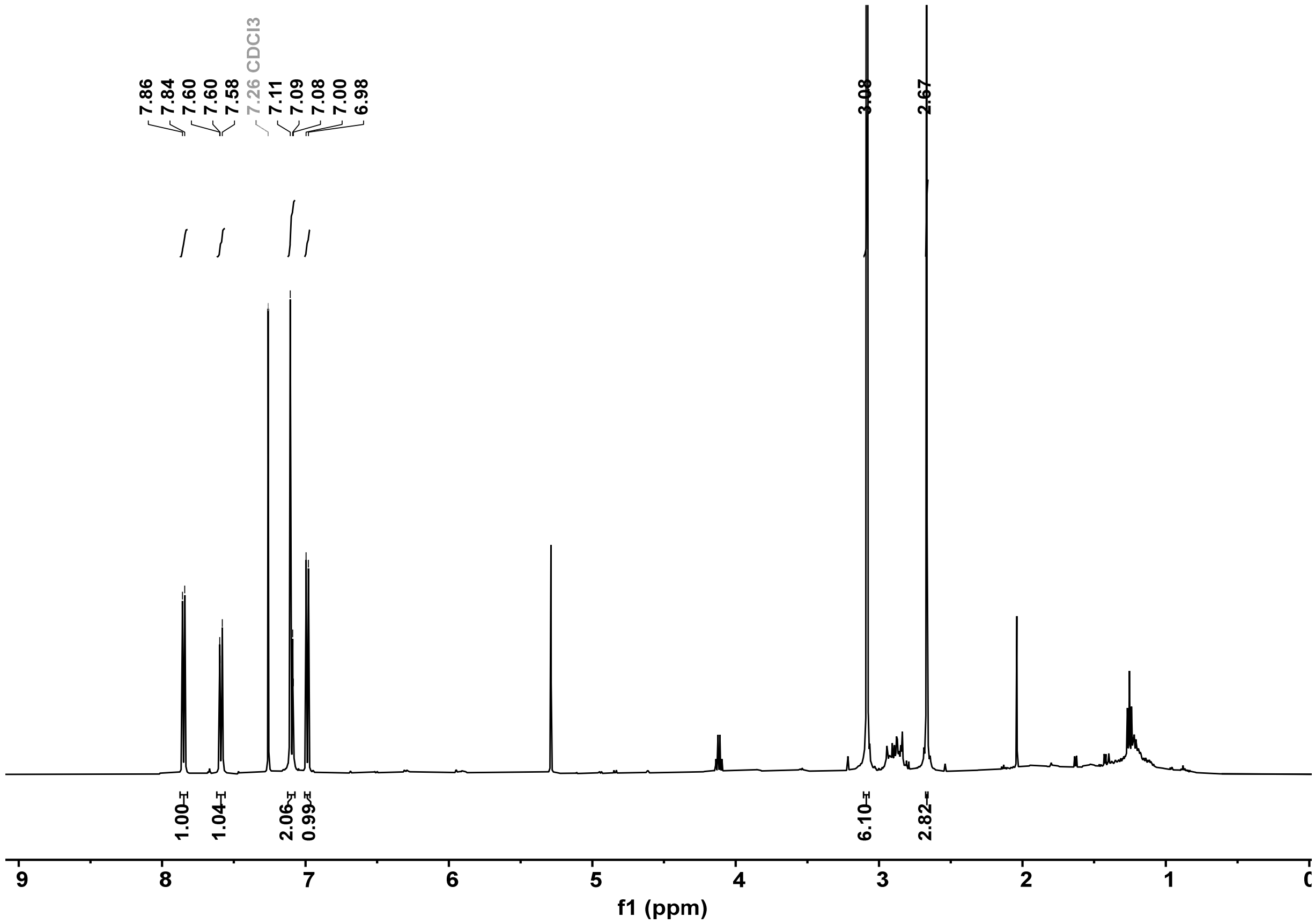

^{1}H ($CDCl_3$)

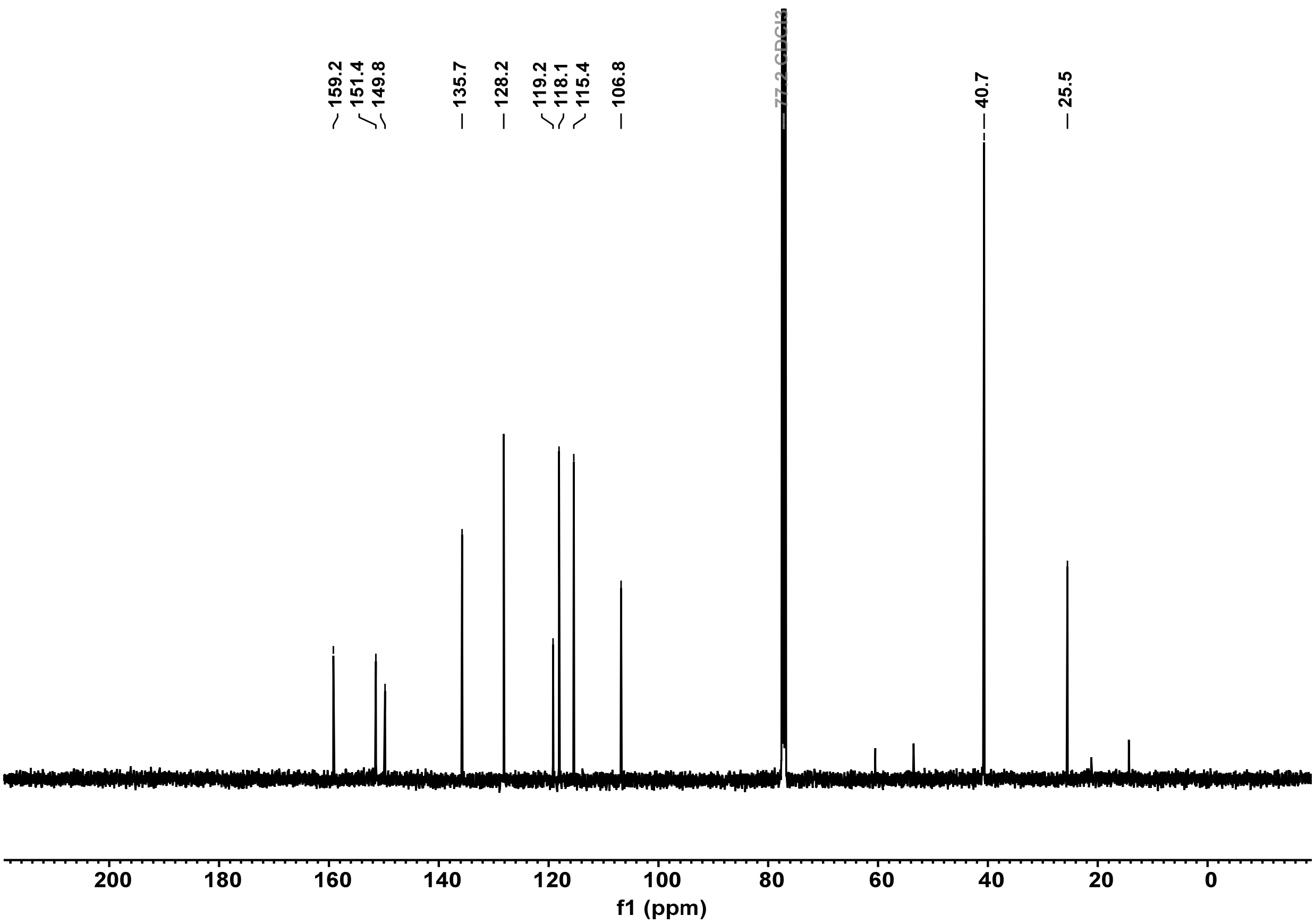

^{13}C ($CDCl_3$)

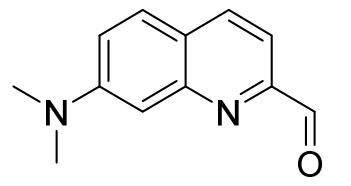

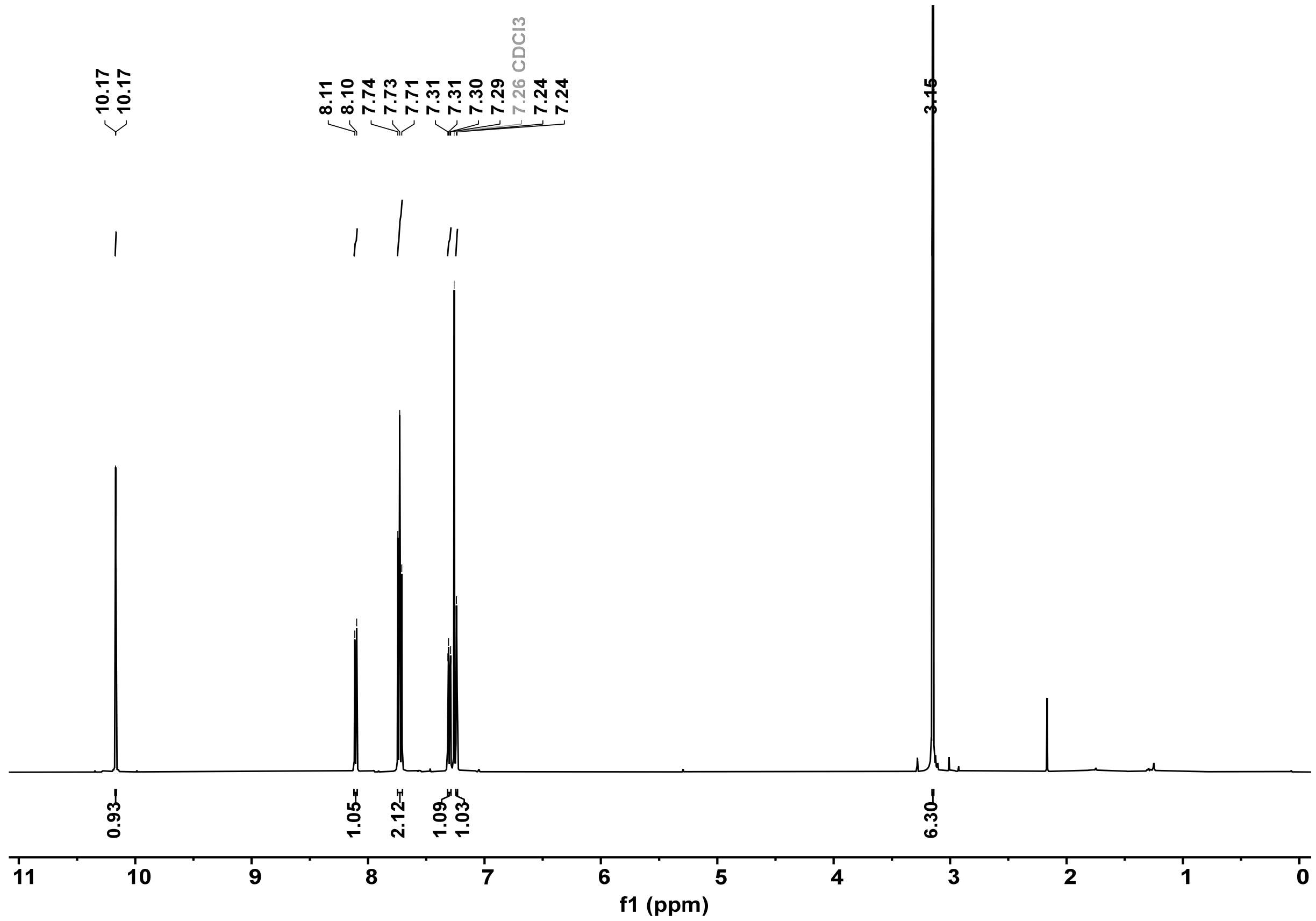

^{1}H ($CDCl_3$)

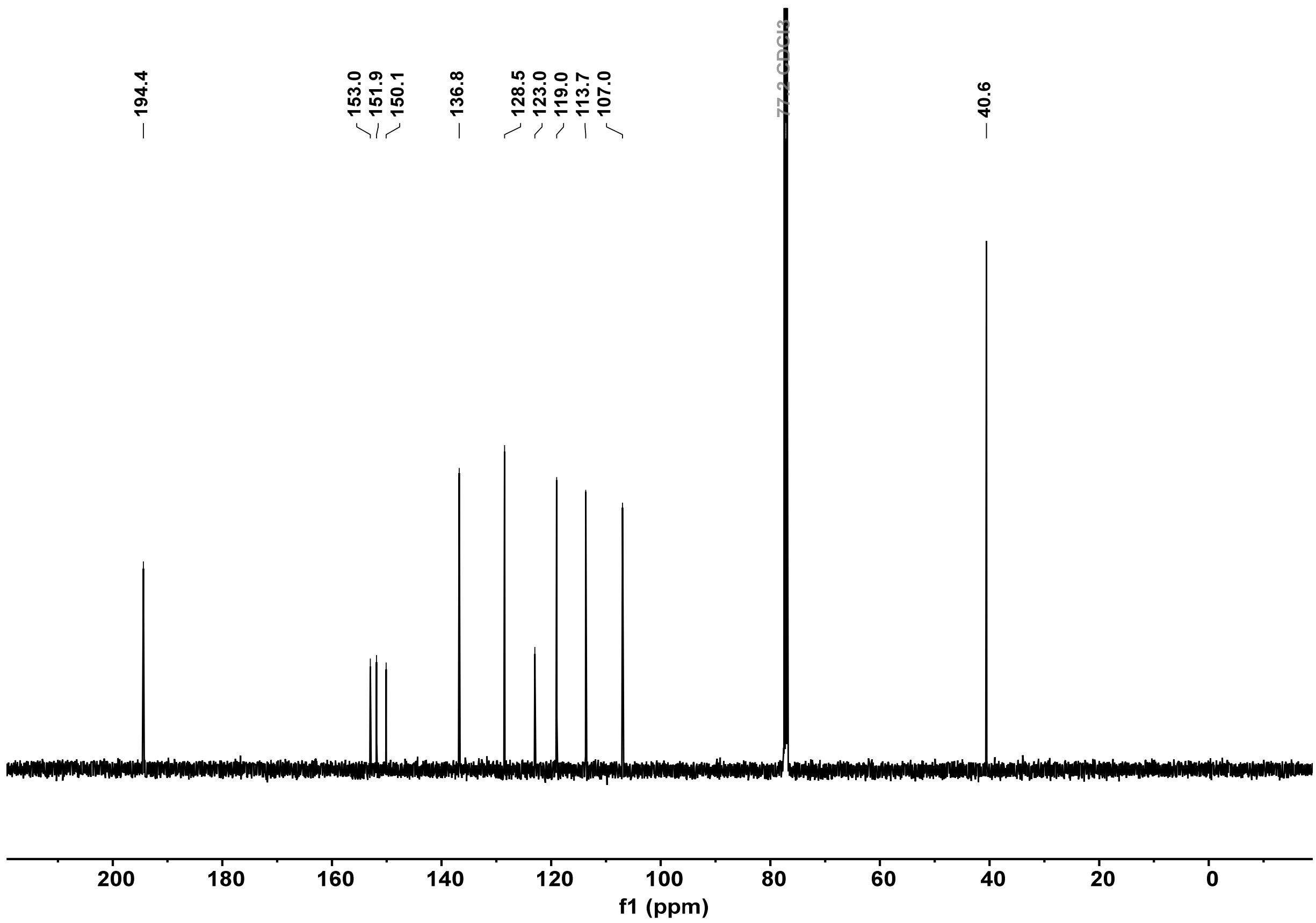

^{13}C ($CDCl_3$)

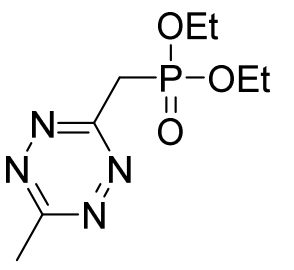

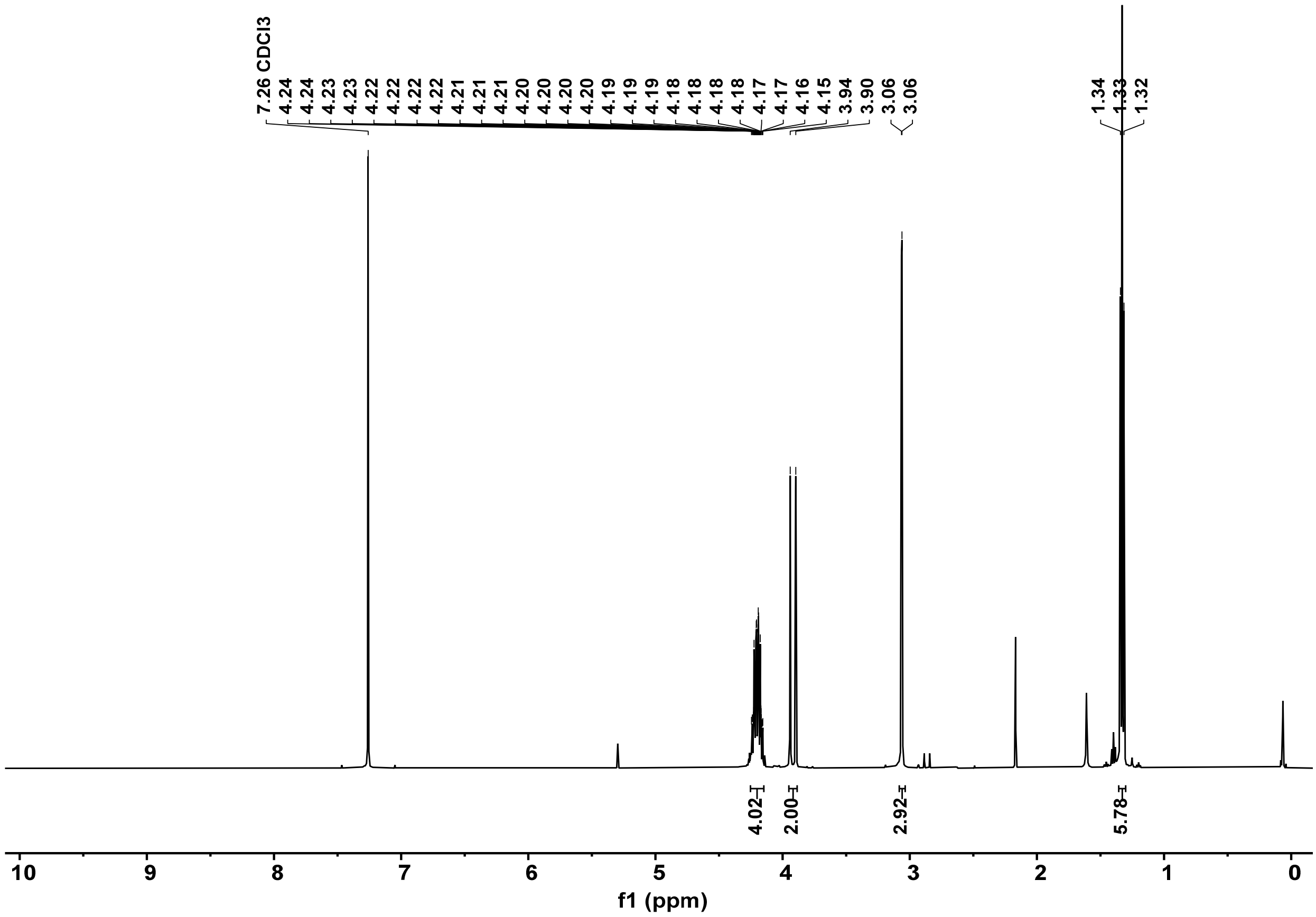

^{1}H ($CDCl_3$)

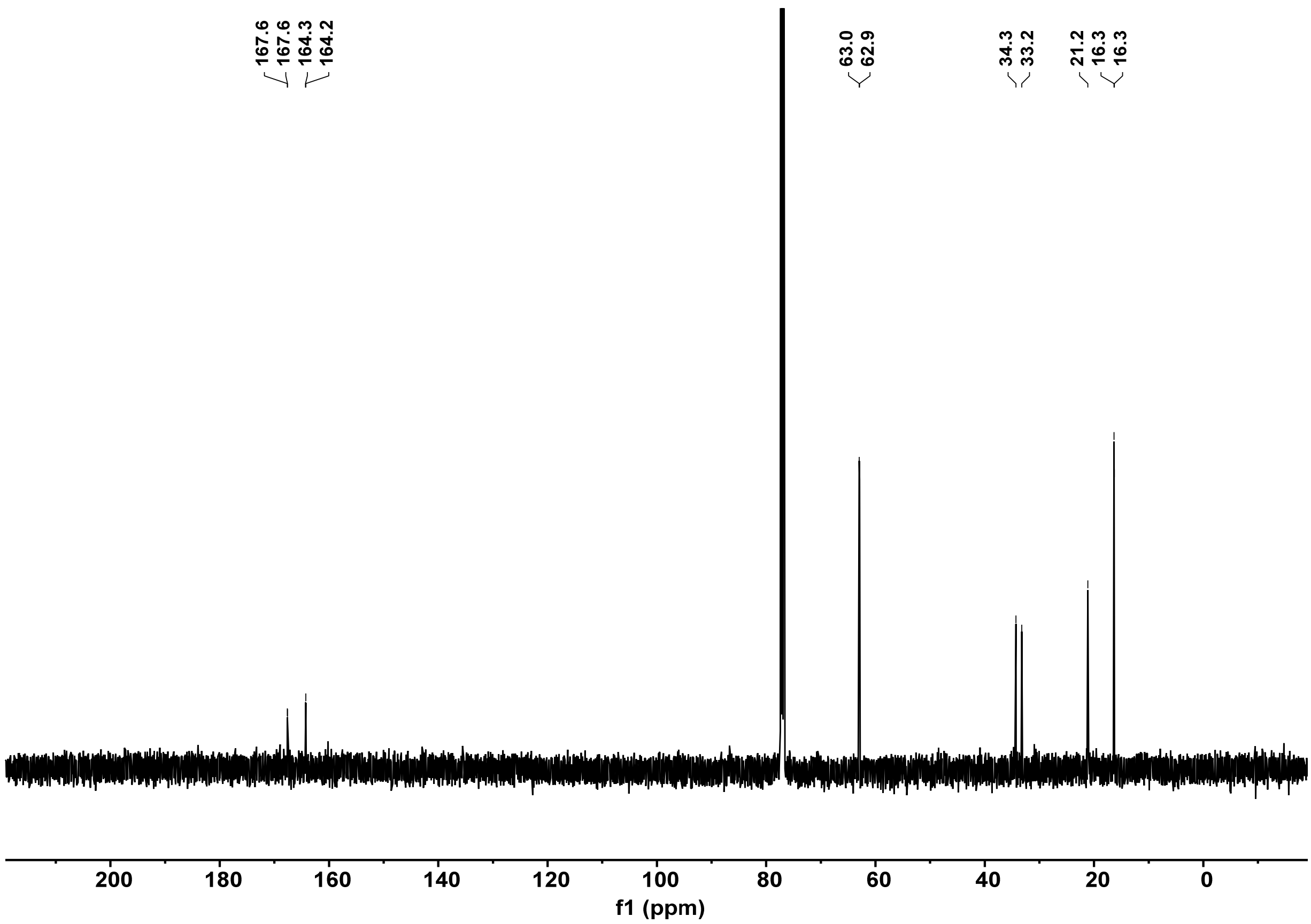

^{13}C ($CDCl_3$)

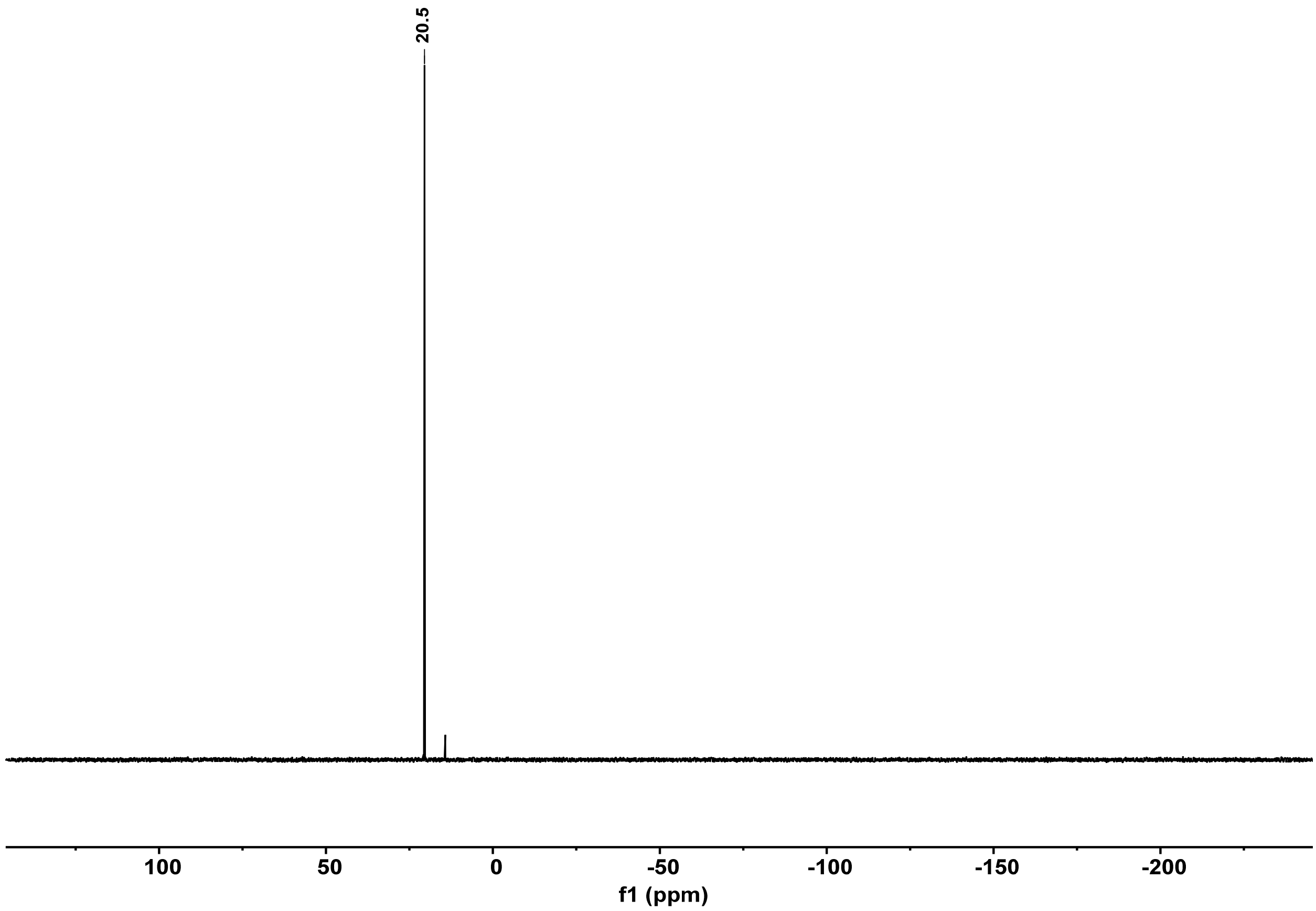

^{31}P ($CDCl_3$)

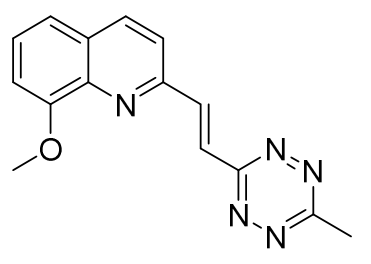

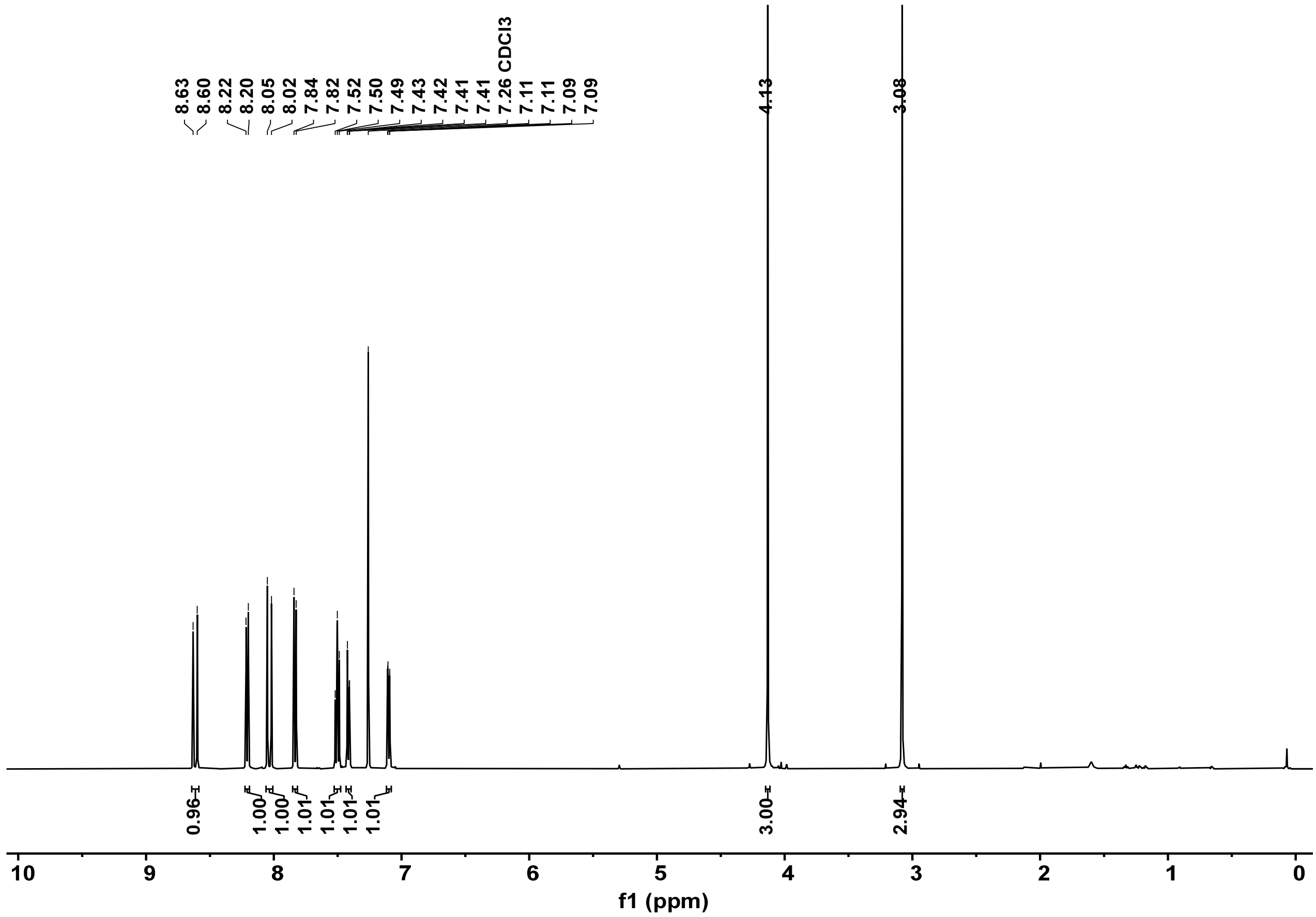

^{1}H ($CDCl_3$)

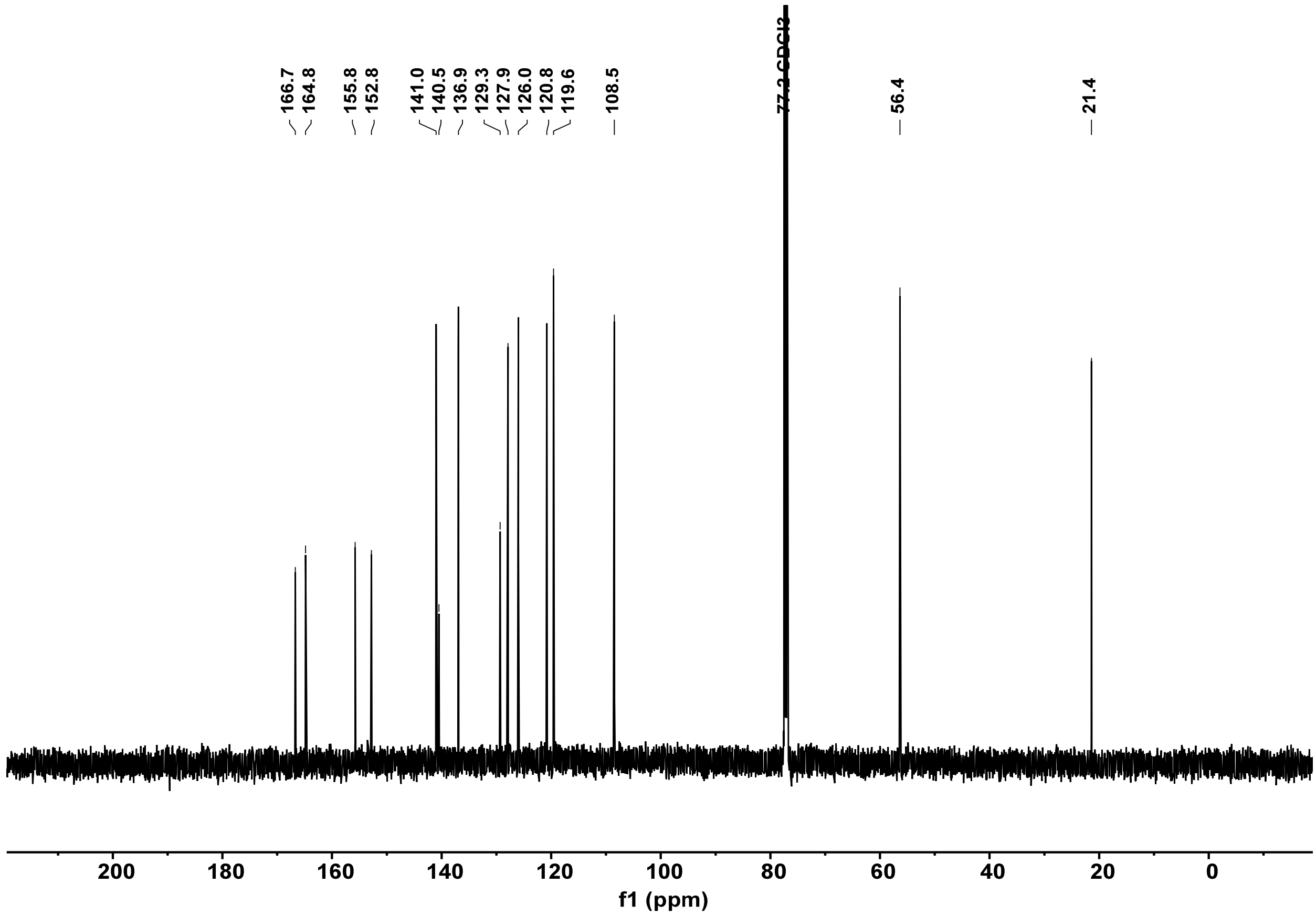

^{13}C ($CDCl_3$)

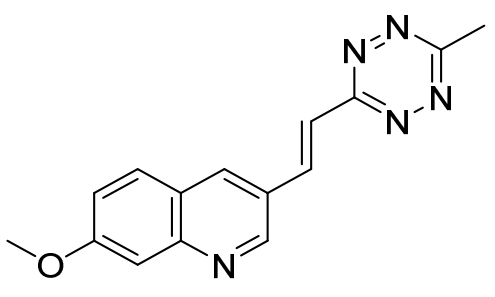

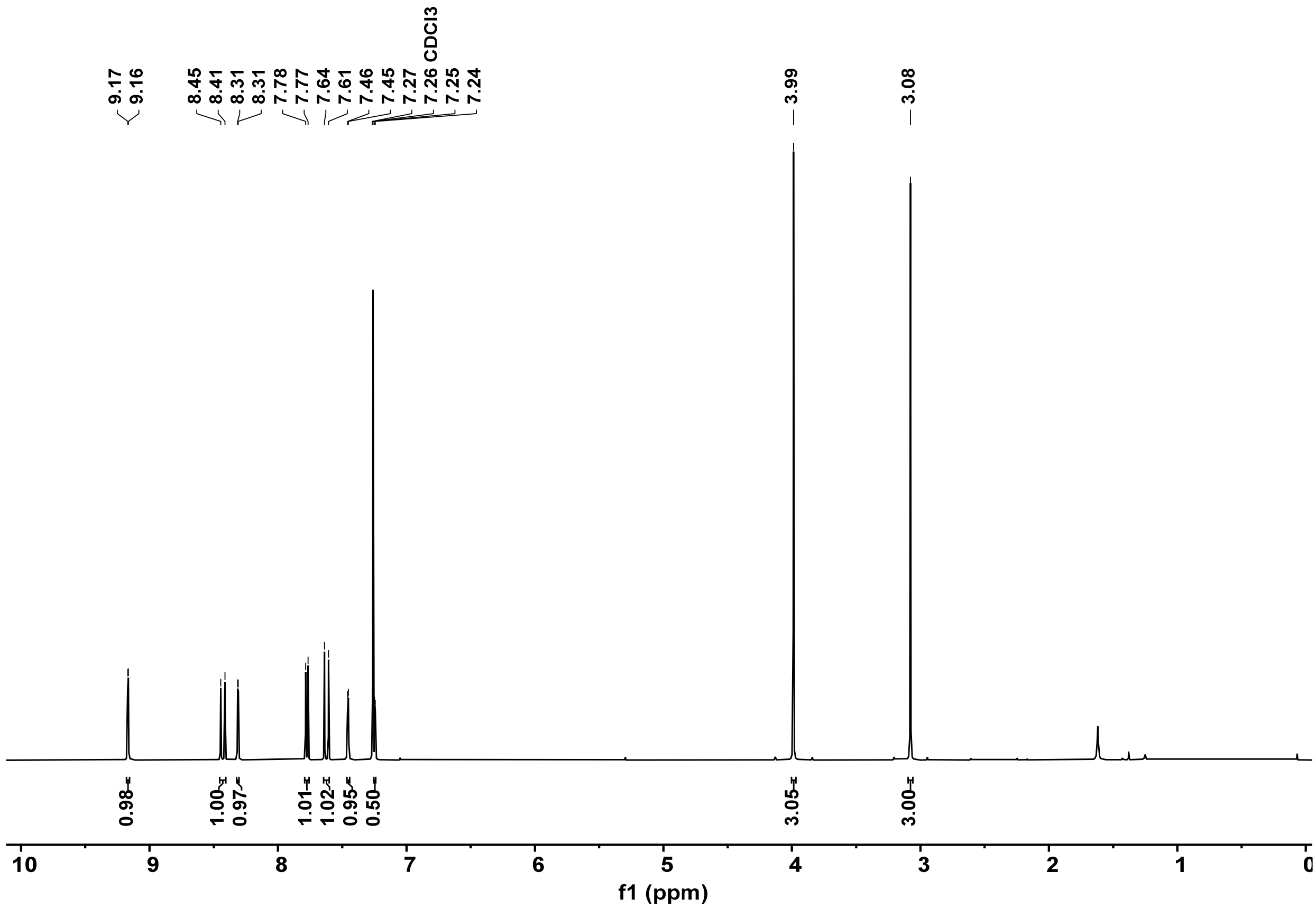

^{1}H ($CDCl_3$)

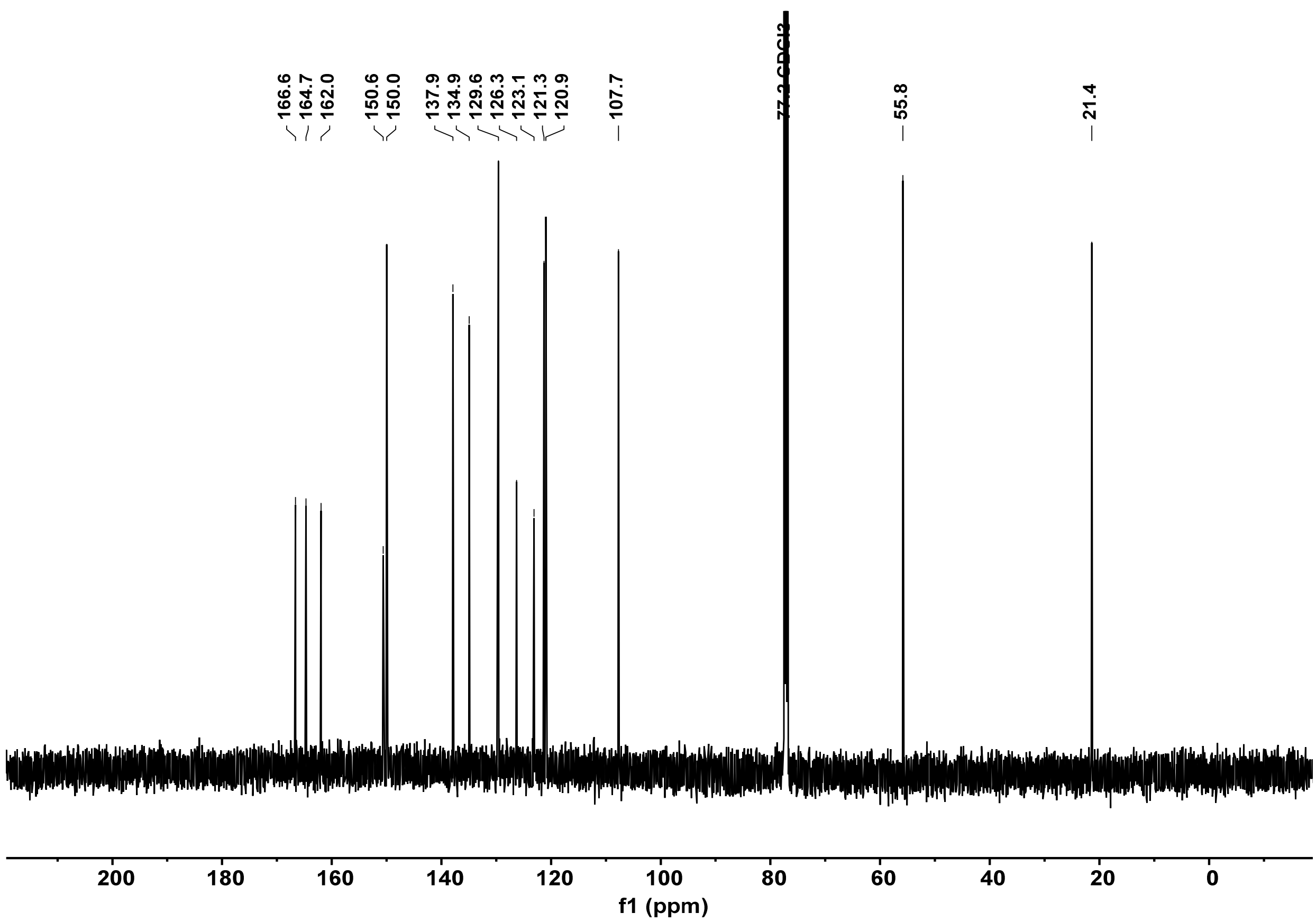

^{13}C ($CDCl_3$)

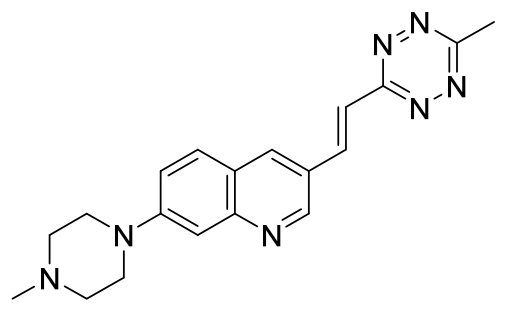

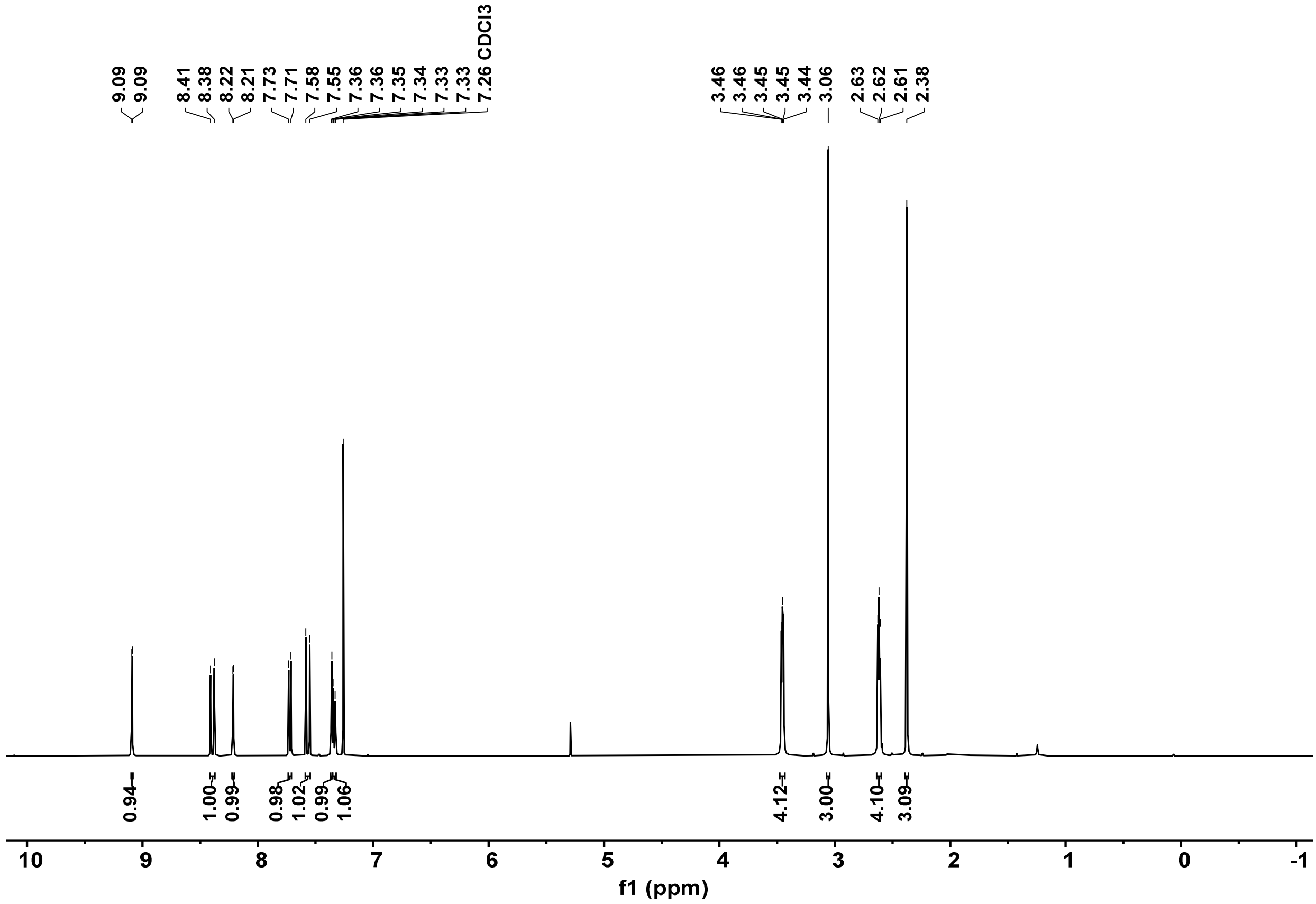

^{1}H ($CDCl_3$)

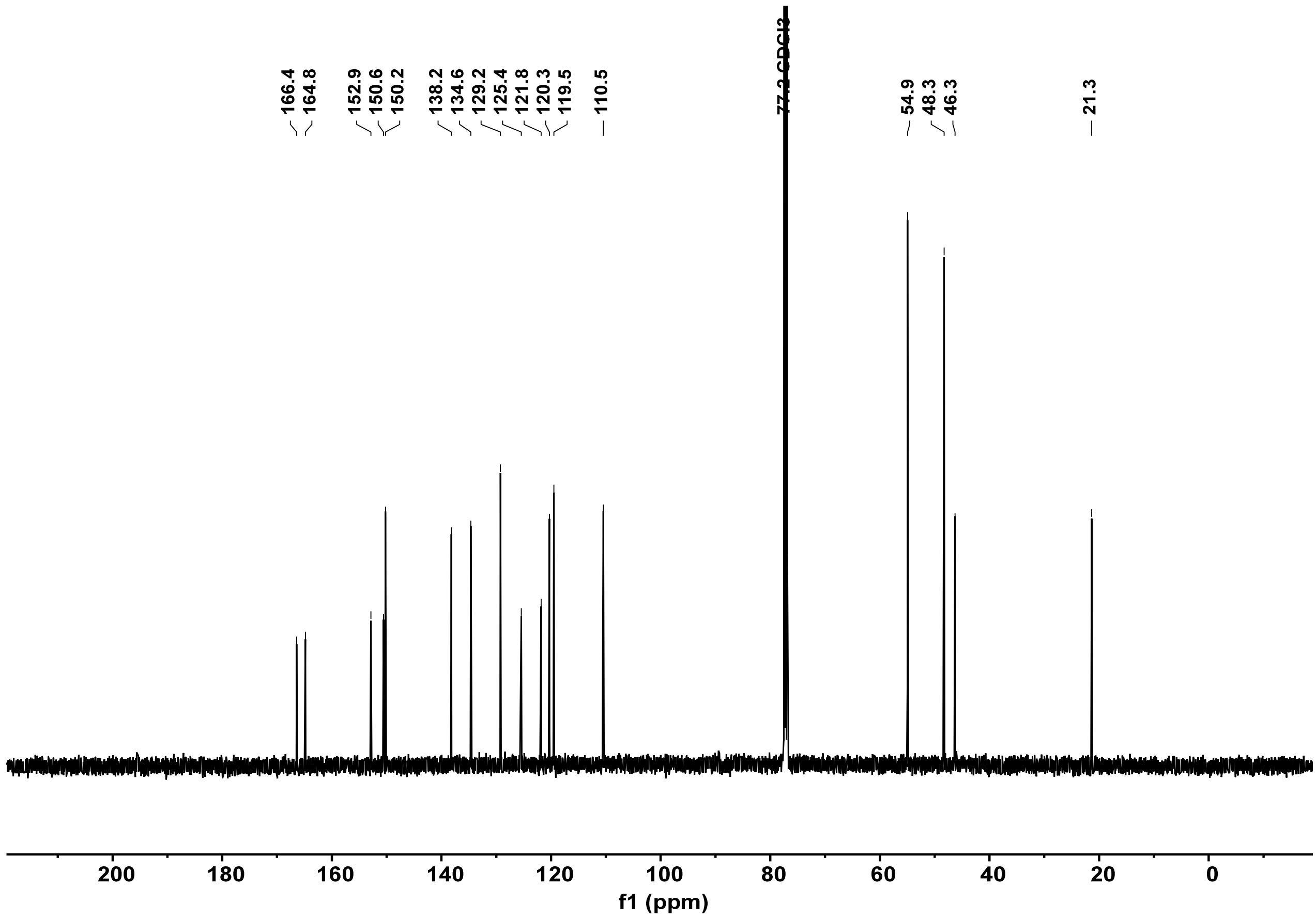

^{13}C ($CDCl_3$)

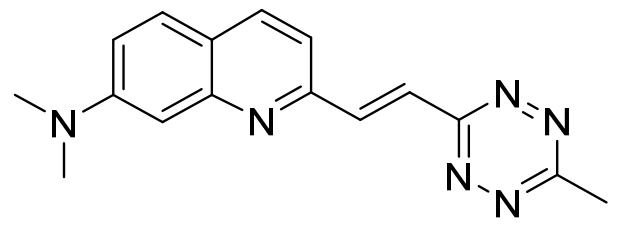

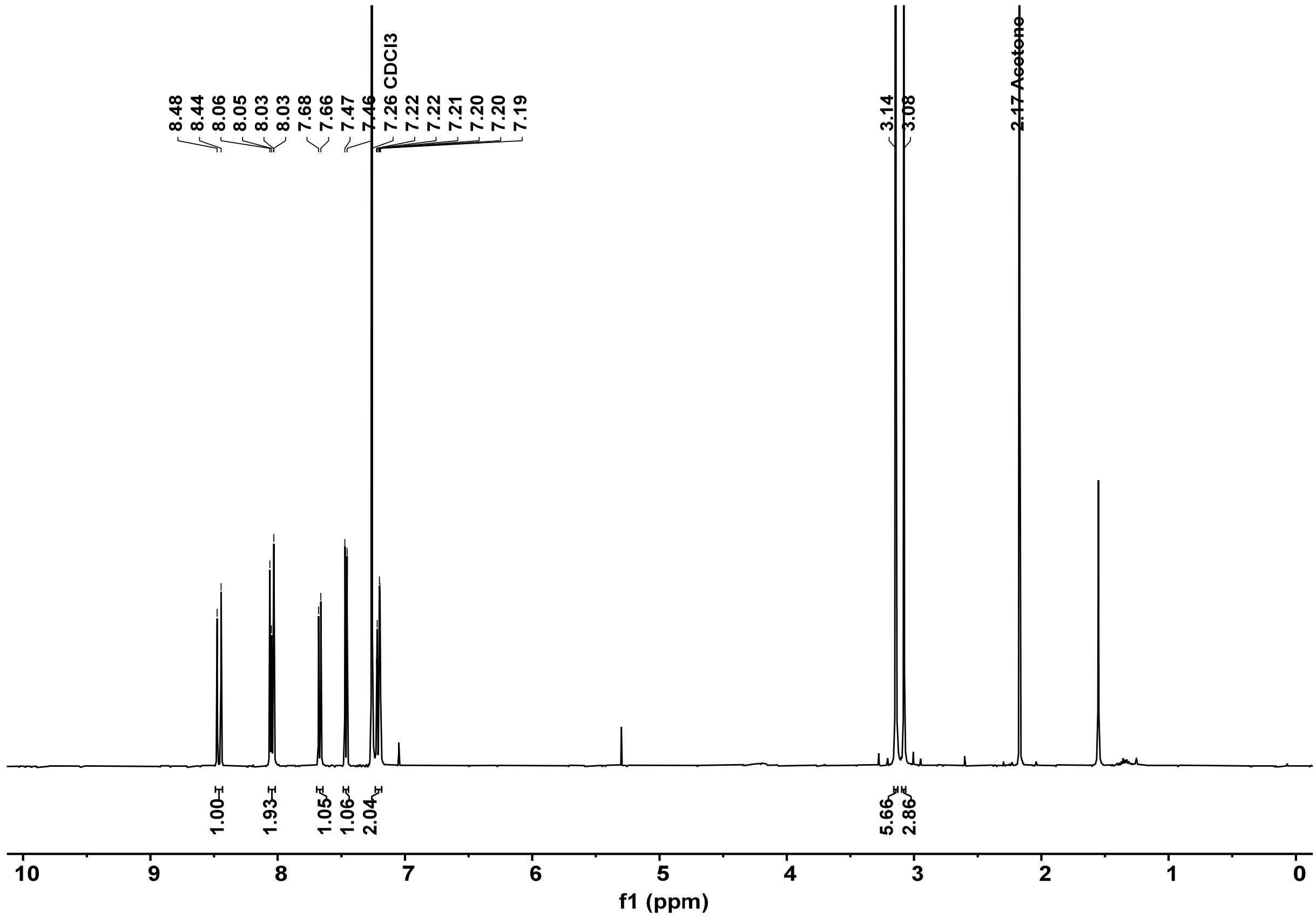

^{1}H ($CDCl_3$)

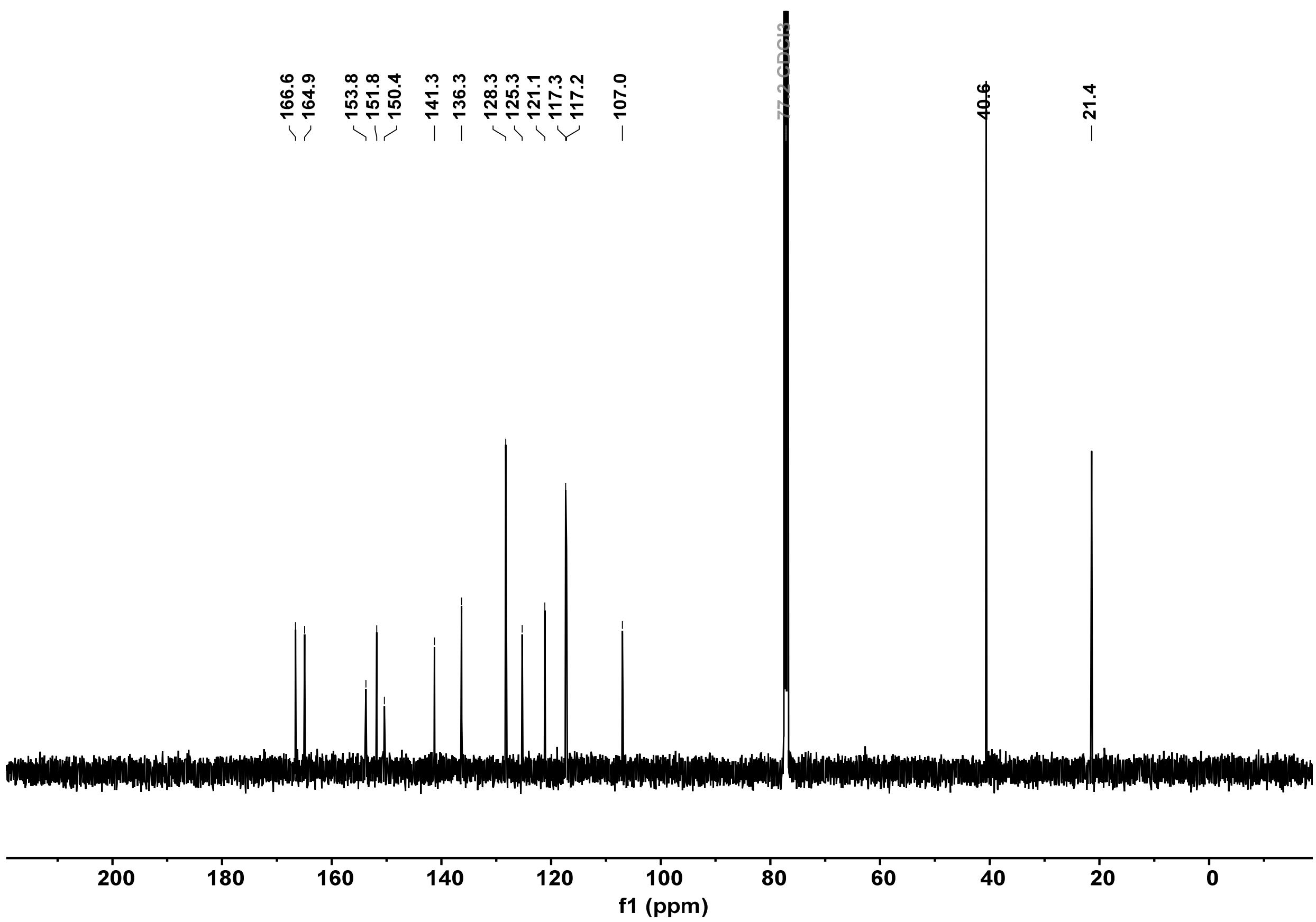

^{13}C ($CDCl_3$)

References

1. U. Rieder and N. W. Luedtke, *Angewandte Chemie International Edition*, 2014, **53**, 9168-9172.
2. M. O. Loehr and N. W. Luedtke, *Angewandte Chemie International Edition*, 2022, **61**, e202112931.
3. G. Cohen and H. Eisenberg, *Biopolymers*, 1969, **8**, 45-55.
4. N. W. Luedtke, P. Carmichael and Y. Tor, *Journal of the American Chemical Society*, 2003, **125**, 12374-12375.
5. M. Slavickova, R. Pohl and M. Hocek, *Journal of Organic Chemistry*, 2016, **81**, 11115-11125.
6. X. Fan, Y. Ge, F. Lin, Y. Yang, G. Zhang, W. S. Ngai, Z. Lin, S. Zheng, J. Wang, J. Zhao, J. Li and P. R. Chen, *Angewandte Chemie International Edition*, 2016, **55**, 14046-14050.
7. J. Yang, M. R. Karver, W. Li, S. Sahu and N. K. Devaraj, *Angewandte Chemie International Edition*, 2012, **51**, 5222-5225.
8. A. J. C. Sarris, T. Hansen, M. A. R. de Geus, E. Maurits, W. Doelman, H. S. Overkleeft, J. D. C. Codee, D. V. Filippov and S. I. van Kasteren, *Chemistry – A European Journal*, 2018, **24**, 18075-18081.
9. A. Heydari, S. Khaksar and M. Tajbakhsh, *Synthesis*, 2008, **2008**, 3126-3130.
10. L. I. Willems, N. Li, B. I. Florea, M. Ruben, G. A. van der Marel and H. S. Overkleeft, *Angewandte Chemie International Edition*, 2012, **51**, 4431-4434.
11. M. Keshavarz-K, S. D. Cox, R. O. Angus Jr and F. Wudl, *Synthesis*, 1988, **1988**, 641-644.
12. N. J. Tom and E. M. Ruel, *Synthesis*, 2001, **2001**, 1351-1355.
13. M. Petit, C. Tran, T. Roger, T. Gallavardin, H. Dhimane, F. Palma-Cerda, M. Blanchard-Desce, F. C. Acher, D. Ogden and P. I. Dalko, *Organic Letters*, 2012, **14**, 6366-6369.
14. W. Mao, W. Shi, J. Li, D. Su, X. Wang, L. Zhang, L. Pan, X. Wu and H. Wu, *Angewandte Chemie International Edition*, 2019, **58**, 1106-1109.
